# Peripheral Blood Gene Expression Profile of Infants with Atopic Dermatitis

**DOI:** 10.1016/j.xjidi.2022.100165

**Published:** 2022-10-07

**Authors:** Janna Nousbeck, Maeve A. McAleer, Alan D. Irvine

**Affiliations:** 1 National Children’s Research Centre, Dublin, Ireland; 2 Clinical Medicine, School of Medicine, Trinity College Dublin, The University of Dublin, Dublin, Ireland; 3 Department of Paediatric Dermatology, Children’s Health Ireland at Crumlin, Dublin, Ireland

**Keywords:** AD, atopic dermatitis, BP, biological process, CC, cellular component, DEG, differentially expressed gene, GO, gene ontology, MF, molecular function, PIP, protein‒protein interaction, RNA-seq, RNA sequencing

## Abstract

To enhance the understanding of molecular mechanisms and mine previously unidentified biomarkers of pediatric atopic dermatitis, PBMC gene expression profiles were generated by RNA sequencing in infants with atopic dermatitis and age-matched controls. A total of 178 significantly differentially expressed genes (DEGs) (115 upregulations and 63 downregulations) were seen, compared with those in healthy controls. The DEGs identified included *IL1β*, *TNF*, *TREM1*, *IL18R1*, and *IL18RAP*. DEGs were validated by real-time RT- qPCR in a larger number of samples from PBMCs of infants with atopic dermatitis aged <12 months. Using the DAVID (Database for Annotation, Visualization and Integrated Discovery) database, functional and pathway enrichment analyses of DEGs were performed. Gene ontology enrichment analysis showed that DEGs were associated with immune responses, inflammatory responses, regulation of immune responses, and platelet activation. Pathway analysis indicated that DEGs were enriched in cytokine‒cytokine receptor interaction, immunoregulatory interactions between lymphoid and nonlymphoid cells, hematopoietic cell lineage, phosphoinositide 3-kinase‒protein kinase B signaling pathway, NK cell‒mediated cytotoxicity, and platelet activation. Furthermore, the protein‒protein interaction network was predicted using the STRING (Search Tool for the Retrieval of Interacting Genes/Proteins) database and visualized with Cytoscape software. Finally, on the basis of the protein‒protein interaction network, 18 hub genes were selected, and two significant modules were obtained. In conclusion, this study sheds light on the molecular mechanisms of pediatric atopic dermatitis and may provide diagnostic biomarkers and therapeutic targets.

## Introduction

Atopic dermatitis (AD) is the most common chronic inflammatory skin disease in early childhood. It affects children with a prevalence of up to 20% and adults with prevalence rates of 7‒10% (Weidinger et al., 2018). AD is a complex multifactorial disease, thought to result from an interplay between environmental factors, an impaired skin barrier, and immune dysfunctions; however, the overall pathologic mechanisms are still not fully understood (Langan et al., 2020). Although much has been learned about the molecular basis of AD, most investigations have focused on adult AD with years of disease activity that is remarkably different from those of early-onset AD in children. Few studies have profiled skin tissue in infants with AD (Brunner et al., 2019, 2018; Cole et al., 2014; Esaki et al., 2016). Over the last decade, RNA sequencing (RNA-seq)-based transcriptome profiles have been implemented in identifying transcripts and pathways in many diseases; however, limited studies using this method were performed on skin tissues in AD, particular in children with AD (Brunner et al., 2019; Cole et al., 2014). Only one study conducted has compared transcriptome profiles of both blood and skin tissue in children with AD at various ages up to age 5 years (Brunner et al., 2019).

Given that AD is an early childhood disease that generates a systemic immunological response and that 60% of all cases of AD begin during the first year of life (Bieber, 2008), we aimed to discover signature biomarkers of AD in infants that might help to identify new diagnostic biomarkers and molecular targets for treatment modalities in pediatric AD.

For this purpose, we performed an integrative study comprising RNA-seq transcriptome profile of peripheral blood cells obtained from infants with AD or healthy infants, quantitative RT-PCR, and systems biology analysis.

## Results

### Analysis of gene expression by RNA-seq

**Figure 1:**
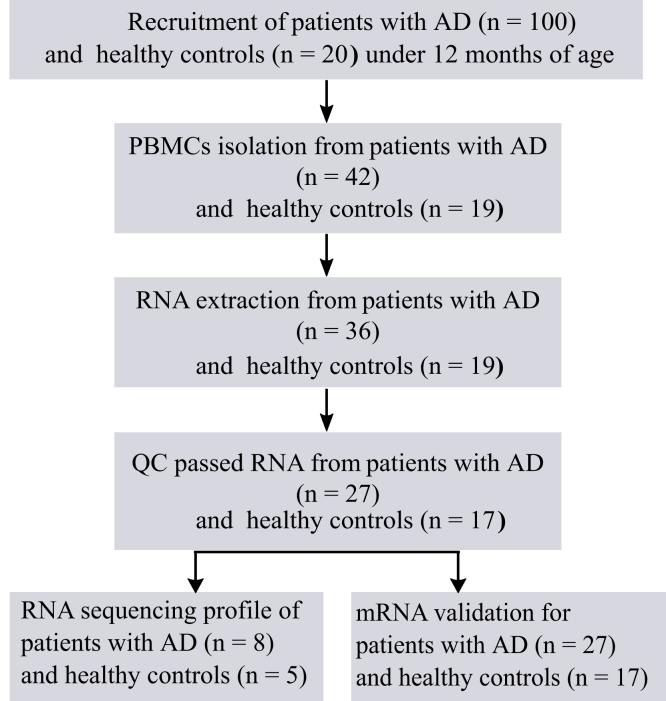
**The overall framework of study design.** AD, atopic dermatitis; QC, quality control.

A total of 100 infants with moderate or severe AD in the first year of life and 20 age-matched healthy control infants were initially recruited (McAleer et al., 2019). PBMCs were isolated from 42 patients and 19 controls. RNA samples were extracted, and only 27 samples from patients with AD and 17 controls passed quality control and were used in this study. The use of samples is presented in a schematic flow chart (Figure 1). We performed RNA-seq profiles on PBMCs from randomly selected infants with AD (n = 8) and controls (n = 5) using the Illumina platform. Differential expression analysis was conducted to identify differentially expressed genes (DEGs) between AD and controls on the basis of the following criteria: false discovery rate <0.05 and fold change ≥1.5. We identified a total of 178 significantly DEGs with 115 upregulations and 63 downregulations in AD PBMCs when compared with those in control PBMCs. Among highly upregulated genes, we identified *IL1β*, previously shown to be upregulated in the serum of adult patients with AD (Thijs et al., 2018); *TNF*, a proinflammatory cytokine whose role in the pathogenesis of AD is well-known (Jacobi et al., 2005; Sumimoto et al., 1992); and early growth response genes *EGR2* and *EGR3*, known to have a crucial role in the regulation of the immune system (Li et al., 2012). Other upregulated genes included *TREM1*, previously shown to be elevated in lesional skin and serum in patients with AD (Suarez-Farinas et al., 2015), and *CXCL5*, an inflammatory chemokine found to be at elevated levels in the blood of patients with AD (Brunner et al., 2017). Among downregulated genes, we identified *IL18R1* and *IL18RAP* found to be associated with AD (Hirota et al., 2012). We summarized the 10 DEGs randomly selected from our study and compared their expression with published data (Table 1). A complete list of DEGs in blood cells is shown in Table 2.

**Table 1:** A List of 10 Differentially Expressed Genes Randomly Selected from Our Study and Their Changes Identified in Older Children and Adults with AD on the Basis of the Literature

DEG	Children Aged 0‒1 y	Tissue, Down/Up	Older Children	Tissue, Down/Up	Adults	Tissue, Down/Up
*IL18RAP*	This study	PBMCs; Down	Brunner et al. (2019) 4 mo, 5 y	AD LS versus HC skin; Up	Ewald et al. (2015) and Hirota et al. (2012)	Susceptibility loci for AD; AD LS versus NS, Up
*IL18R1*	This study	PBMCs; Down	NA		Hirota et al*.* (2012)	Susceptibility loci for AD
*IL1β*	This study	PBMCs; Up	Cole et al. (2014) 6‒16 years	AD NS versus HC skin; Down	McAleer et al. (2019) and Thijs et al. (2018)	AD versus HC Serum, Up; AD versus HC SC, Down
*TNF*	This study	PBMCs; Up	Brunner et al. (2019) Sumimoto et al. (1992) 1‒15 y	AD LS versus HC skin, Up; AD NS versus HC skin, Up; AD versus HC Plasma, Up	Suarez-Farinas et al. (2015) and Thijs et al. (2018)	AD LS versus NS, Up AD versus HC Serum, Up
*TREM1*	This study	PBMCs; Up	NA		Suarez-Farinas et al. (2015) and Thijs et al. (2018)	AD versus HC Serum, Up AD LS versus NS, Up
*EGR3*	This study	PBMCs; Up	NA		Suarez-Farinas et al. (2011)	AD LS versus HC skin, Down; AD NS versus HC skin, Down
*EGR2*	This study	PBMCs; Up	Brunner et al. (2019) 4 mo, 5 y	AD LS versus HC skin, Down; AD NS versus HC skin, Down	Hirota et al*.* (2012)	Susceptibility loci for AD
*EGR1*	This study	PBMCs; Up	Brunner et al. (2019) 4 mo, 5 y Cole et al. (2014) 6‒16 y	AD LS versus HC skin, Down; AD NS versus HC skin, Down; AD NS versus HC skin; Up	Suarez-Farinas et al. (2011)	AD LS versus HC skin, Up; AD NS versus HC skin, Up
*NLRP3*	This study	PBMCs; Up	Brunner et al. (2019)	AD LS versus HC skin, Down; AD NS versus HC skin, Down	Niebuhr et al. (2014)	AD LS versus HC skin, Down
*FOSL1*	This study	PBMCs; Up	Brunner et al. (2019) 6‒16 y Cole et al. (2014) 6‒16 y	AD LS versus HC skin, Up AD NS versus HC skin; Up	Ewald et al. (2015) and Suarez-Farinas et al. (2011)	AD LS versus HC skin, Up; AD LS versus AD NL skin, Up; AD LS versus NS, Up

Abbreviations: AD, atopic dermatitis; DEG, differentially expressed gene; Down, downregulated; HC, healthy control; LS, lesional skin; NA, nonavailable; NS, nonlesional skin; SC, stratum corneum; Up, upregulated.

**Table 2:** Differentially Expressed Genes in PBMCs from Infants with AD

Gene	Log_2_FC	FC	*P*-Value	FDR
*GLDC*	‒2.265	0.208	3.34E**−**11	5.29E**−**07
*HRASLS2*	‒2.086	0.236	2.18E**−**05	0.00576
*SDC1*	‒1.809	0.285	0.00044	0.03940
*CDC20*	‒1.749	0.297	6.14E**−**05	0.01085
*IL18RAP*	‒1.702	0.307	1.56E**−**07	0.00031
*INTU*	‒1.664	0.316	2.70E**−**05	0.00658
*IGKV3-11*	‒1.655	0.317	0.00031	0.03247
*KIR2DL3*	‒1.524	0.348	6.36E**−**05	0.01095
*AL645929.1*	‒1.497	0.354	4.41E**−**05	0.00906
*IGKV5-2*	‒1.487	0.357	1.46E**−**07	0.00031
*PLEKHG7*	‒1.472	0.360	1.29E**−**05	0.00474
*BHLHA15*	‒1.437	0.369	0.00021	0.02593
*IGHV3-21*	‒1.412	0.376	0.00038	0.03721
*GTSE1*	‒1.402	0.378	1.17E**−**05	0.00465
*AC007278.2*	‒1.389	0.382	0.00032	0.03341
*FASLG*	‒1.367	0.388	7.97E**−**05	0.01202
*MCM10*	‒1.357	0.390	4.54E**−**05	0.00923
*NCAPG*	‒1.350	0.392	1.58E**−**05	0.00534
*IGJ*	‒1.349	0.393	0.00015	0.02018
*SPC24*	‒1.328	0.398	2.10E**−**06	0.00210
*UBE2C*	‒1.326	0.399	0.00038	0.03721
*AL365475.1*	‒1.321	0.400	7.85E**−**05	0.01196
*CCDC150*	‒1.276	0.413	0.00016	0.02125
*KLRD1*	‒1.257	0.418	7.68E**−**05	0.01192
*MELK*	‒1.255	0.419	0.00010	0.01551
*SKA3*	‒1.252	0.420	0.00013	0.01832
*AC007278.1*	‒1.226	0.428	0.00063	0.04921
*IGLV1-44*	‒1.225	0.428	3.86E**−**05	0.00849
*NCR1*	‒1.207	0.433	0.00056	0.04636
*IGLV3-19*	‒1.192	0.438	2.91E**−**05	0.00687
*KLRC4*	‒1.176	0.443	0.00020	0.02444
*RNF165*	‒1.134	0.456	0.00046	0.04029
*IGLV8-61*	‒1.111	0.463	7.35E**−**05	0.01176
*MYBL1*	‒1.104	0.465	2.63E**−**08	0.00011
*AC006480.2*	‒1.093	0.469	0.00013	0.01832
*AURKB*	‒1.073	0.475	0.00026	0.02917
*SLCO4A1*	‒1.057	0.481	7.15E**−**05	0.01167
*IGLV2-8*	‒1.057	0.481	0.00022	0.02608
*IGLV2-23*	‒1.037	0.487	2.85E**−**05	0.00684
*IL18R1*	‒1.030	0.490	5.41E**−**08	0.00014
*IGLC2*	‒1.022	0.493	0.00019	0.02387
*AL683813.1*	‒1.020	0.493	0.00058	0.04670
*AC010536.1*	‒1.013	0.496	0.00056	0.04625
*STRIP2*	‒1.002	0.499	0.00030	0.03156
*KLRF1*	‒0.993	0.502	0.00023	0.02686
*XYLB*	‒0.971	0.510	0.00039	0.03721
*SH2D2A*	‒0.964	0.513	0.00018	0.02284
*IGKV1-5*	‒0.958	0.515	1.51E**−**05	0.00519
*IGKV4-1*	‒0.958	0.515	0.00011	0.01580
*IGLV2-11*	‒0.947	0.519	4.07E**−**05	0.00859
*PIWIL2*	‒0.931	0.525	0.00042	0.03910
*SLFN13*	‒0.890	0.540	8.03E**−**06	0.00340
*IGKV1-12*	‒0.870	0.547	0.00055	0.04604
*PDGFD*	‒0.834	0.561	0.00018	0.02275
*ABCB9*	‒0.821	0.566	0.00043	0.03940
*ISG20*	‒0.796	0.576	0.00025	0.02887
*KIF2C*	‒0.782	0.582	0.00037	0.03676
*IGKV3-15*	‒0.777	0.584	0.00057	0.04636
*TTC22*	‒0.762	0.590	0.00044	0.03940
*C5orf56*	‒0.705	0.613	2.15E**−**05	0.00576
*DLG3*	‒0.696	0.617	0.00054	0.04512
*COLQ*	‒0.609	0.656	3.98E**−**05	0.00859
*SPATS2*	‒0.608	0.656	5.20E**−**05	0.00992
*APP*	0.598	1.514	4.61E**−**08	0.00014
*MCL1*	0.661	1.581	4.80E**−**06	0.00256
*MAML3*	0.661	1.581	1.85E**−**05	0.00576
*KCNQ1*	0.732	1.661	0.00040	0.03721
*RFX2*	0.754	1.687	7.45E**−**05	0.01180
*PGRMC1*	0.779	1.716	6.33E**−**05	0.01095
*PRXL2C*	0.786	1.724	0.00061	0.04852
*FRMD4B*	0.842	1.793	0.00036	0.03648
*ANXA1*	0.869	1.827	5.64E**−**05	0.01051
*ESAM*	0.912	1.881	0.00049	0.04315
*HIST1H2AC*	0.916	1.887	0.00045	0.04004
*RAPH1*	0.923	1.896	0.00028	0.03031
*ADAM9*	0.940	1.918	0.00027	0.03031
*TUBA1A*	1.021	2.029	0.00060	0.04766
*LDLR*	1.068	2.097	1.29E**−**05	0.00474
*CPNE2*	1.098	2.140	3.93E**−**06	0.00244
*ZNF185*	1.183	2.270	0.00055	0.04604
*MYADM*	1.187	2.278	6.10E**−**05	0.01085
*F5*	1.301	2.464	2.45E**−**06	0.00210
*AHR*	1.343	2.536	0.00058	0.04670
*LMNA*	1.382	2.606	7.10E**−**05	0.01167
*HIST2H2BE*	1.467	2.765	4.13E**−**06	0.00244
*GNG11*	1.491	2.810	0.00023	0.02700
*HIST1H2BJ*	1.560	2.948	0.00033	0.03358
*FAM129B*	1.570	2.968	0.00034	0.03455
*SH3BGRL2*	1.617	3.067	0.00028	0.03031
*PADI4*	1.625	3.085	0.00040	0.03721
*LTBP1*	1.635	3.106	0.00057	0.04636
*ICAM1*	1.645	3.128	0.00057	0.04636
*FAM20C*	1.662	3.165	0.00049	0.04315
*C2orf88*	1.675	3.194	2.45E**−**05	0.00625
*NAMPT*	1.701	3.252	0.00029	0.03156
*CD14*	1.703	3.256	0.00042	0.03898
*SCN1B*	1.728	3.312	5.16E**−**05	0.00992
*NRGN*	1.740	3.340	1.77E**−**05	0.00560
*LGALS12*	1.748	3.359	0.00011	0.01596
*GGTA1P*	1.751	3.367	0.00026	0.02917
*PPP1R15A*	1.762	3.392	0.00038	0.03721
*SEPT5*	1.765	3.399	3.99E**−**06	0.00244
*TAL1*	1.767	3.402	2.23E**−**06	0.00210
*PDGFC*	1.769	3.408	6.82E**−**05	0.01149
*PRKAR2B*	1.781	3.436	1.96E**−**06	0.00210
*RAB20*	1.807	3.499	0.00039	0.03721
*PLAUR*	1.810	3.505	0.00051	0.04372
*CTTN*	1.813	3.513	1.97E**−**05	0.00576
*GP6*	1.813	3.515	1.72E**−**-05	0.00557
*GAS2L1*	1.834	3.565	0.00016	0.02125
*CLDN5*	1.864	3.641	0.00018	0.02321
*PEAR1*	1.867	3.649	1.71E**−**05	0.00557
*KLF4*	1.871	3.657	0.00039	0.03721
*SOWAHC*	1.872	3.661	0.00051	0.04372
*TSPAN9*	1.879	3.679	4.20E**−**06	0.00244
*MMP25*	1.903	3.740	7.48E**−**06	0.00329
*LGALSL*	1.942	3.843	4.86E**−**06	0.00256
*ANPEP*	1.943	3.846	7.25E**−**05	0.01171
*SPARC*	1.954	3.875	0.00028	0.03031
*FAXDC2*	1.966	3.907	2.52E**−**06	0.00210
*ATP2B1-AS1*	1.969	3.914	0.00014	0.01999
*ITGA2B*	1.979	3.942	0.00015	0.02018
*NLRP3*	1.995	3.987	4.40E**−**05	0.00906
*ITGB3*	1.999	3.998	0.00016	0.02125
*ITGB5*	2.001	4.004	5.57E**−**05	0.01051
*BEND2*	2.012	4.034	0.00051	0.04372
*TRIB1*	2.015	4.042	0.00030	0.03156
*PTX3*	2.039	4.109	0.00024	0.02744
*VWF*	2.054	4.152	1.32E**−**05	0.00474
*DUSP6*	2.066	4.186	0.00014	0.01973
*ELOVL7*	2.109	4.314	2.10E**−**05	0.00576
*ALOX12*	2.134	4.389	2.96E**−**05	0.00690
*HOMER3*	2.140	4.406	3.76E**−**05	0.00839
*ADM*	2.142	4.414	0.00045	0.04004
*PDE5A*	2.151	4.442	0.00022	0.02608
*TMEM40*	2.166	4.489	0.00039	0.03721
*MPIG6B*	2.178	4.526	1.16E**−**06	0.00147
*ENKUR*	2.178	4.527	0.00062	0.04886
*LRP3*	2.194	4.575	5.93E**−**05	0.01085
*AC245128.3*	2.195	4.579	2.51E**−**05	0.00630
*TUBB1*	2.212	4.633	1.31E**−**05	0.00474
*TREML1*	2.258	4.785	3.55E**−**06	0.00244
*CAVIN2*	2.277	4.847	5.78E**−**06	0.00269
*IER3*	2.290	4.891	6.17E**−**05	0.01085
*WLS*	2.302	4.930	2.16E**−**05	0.00576
*NRIP3*	2.330	5.030	0.00021	0.02593
*EMP1*	2.340	5.062	1.21E**−**06	0.00147
*GP1BA*	2.342	5.069	5.15E**−**06	0.00256
*PF4*	2.349	5.095	2.92E**−**06	0.00231
*TNF*	2.352	5.107	2.47E**−**06	0.00210
*PPBP*	2.379	5.204	9.63E**−**07	0.00139
*SGK1*	2.443	5.439	5.32E**−**06	0.00256
*CLU*	2.464	5.517	0.00052	0.04420
*TREM1*	2.464	5.519	9.14E**−**07	0.00139
*FCAR*	2.472	5.549	1.91E**−**05	0.00576
*LUCAT1*	2.560	5.898	0.00050	0.04362
*GP9*	2.577	5.968	4.27E**−**06	0.00244
*CMTM5*	2.580	5.981	2.02E**−**05	0.00576
*AL391903.1*	2.604	6.079	4.05E**−**05	0.00859
*ABLIM3*	2.620	6.146	2.44E**−**05	0.00625
*CALD1*	2.629	6.185	2.17E**−**05	0.00576
*B3GNT5*	2.681	6.415	6.12E**−**06	0.00277
*AC007032.1*	2.695	6.474	0.00062	0.04876
*SPOCD1*	2.713	6.556	5.34E**−**06	0.00256
*ZNF503*	2.843	7.177	8.15E**−**06	0.00340
*CXCL5*	2.848	7.202	0.00016	0.02125
*SEC14L5*	2.856	7.241	3.18E**−**05	0.00730
*AQP10*	2.879	7.356	0.00044	0.03940
*PDZK1IP1*	3.014	8.080	0.00043	0.03921
*IL1B*	3.127	8.736	7.60E**−**05	0.01192
*SPX*	3.184	9.090	3.38E**−**06	0.00244
*HRAT92*	3.192	9.140	1.82E**−**09	1.44E-05
*CLEC1B*	3.356	10.238	8.70E**−**07	0.00139
*ID1*	3.458	10.986	3.66E**−**05	0.00827
*EGR3*	3.496	11.279	0.00030	0.03156
*EGR1*	3.517	11.447	0.00033	0.03344
*FOSL1*	3.557	11.767	1.48E**−**05	0.00519
*EGR2*	3.763	13.572	0.00024	0.02800

Shown is a list of genes differentially expressed in the blood of children with AD children versus in age-matched healthy control, meeting criteria of FC ≥1.5 and FDR <0.05.

Abbreviations: AD, atopic dermatitis; FC, fold change; FDR, false discovery rate; MMP, matrix metalloproteinase; VWF, Von Willebrand factor.

### Validation of RNA-seq data by RT-qPCR

**Figure 2:**
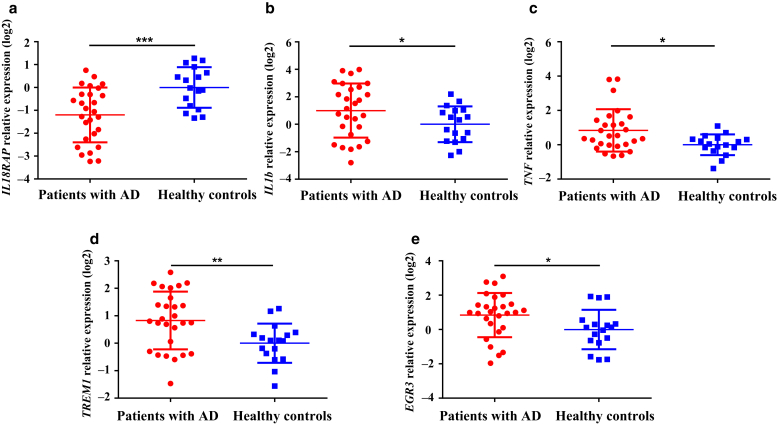
**Validation of RNA-sequencing data by RT-qPCR.** RT-qPCR analyses for five genes from the top 10 differentially expressed genes identified by high-throughput RNA sequencing: (**a**) *IL18RAP*, (**b**) *IL1β*, (**c**) *TNF*, (**d**) *TREM1*, and (**e**) *EGR3* in children with AD (n = 27) and healthy controls (n = 17). Fold change was calculated by 2-ΔΔCT method. The normalized expression data were log_2_ transformed and shown as the means ± SD. Significant difference among groups was calculated by unpaired *t* test with Welch’s correction for normal distribution or with Mann‒Whitney rank-sum test for non-normal distribution data. **∗***P* < 0.05, ∗∗*P* < 0.01, and ∗∗∗*P* < 0.001

To confirm the results of RNA-seq, real-time RT-qPCR was performed to detect the mRNA expression of five randomly selected DEGs in PBMCs from controls (n = 17) and infants with AD (n = 27). As shown in Figure 2a-e, the mRNA levels of all five genes—*IL18RAP*, *IL1β*, *TNF*, *TREM1*, and *EGR3*—had significant differences between the AD and control groups in accordance with RNA-seq results.

### Effect of infant’s age on gene expression in the blood of infants with AD

**Figure 3:**
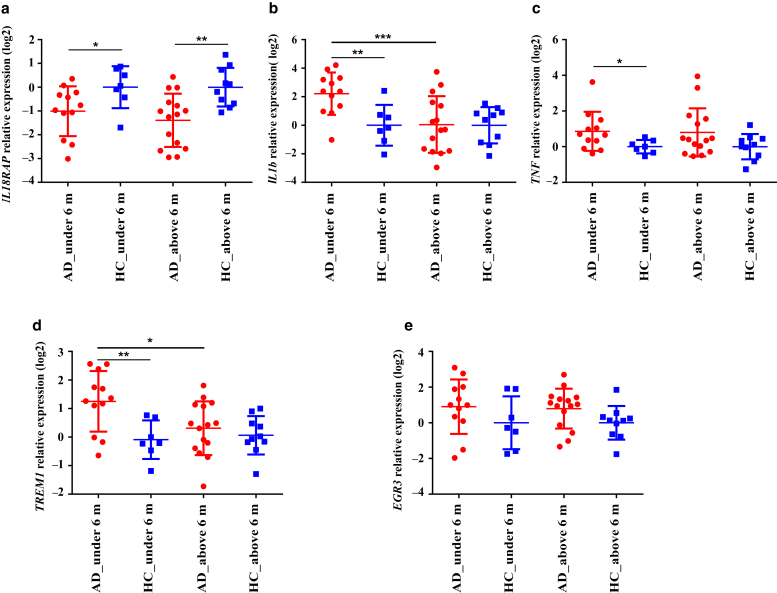
**Effect of infant’s age on the expression of differentially expressed genes in infants with AD.** RT-qPCR analyses for five genes from the top 10 differentially expressed genes identified by high-throughput RNA sequencing: (**a**) *IL18RAP*, (**b**) *IL1β*, (**c**) *TNF*, (**d**) *TREM1*, and (**e**) *EGR3* in children with AD (n = 27) and healthy controls (n = 17) after stratifying the dataset by age (0‒6 months and 7‒12 months). Fold change was calculated by 2-ΔΔCT method. The normalized expression data were log_2_ transformed and shown as the means ± SD. Significant difference among groups was calculated by unpaired *t* test with Welch’s correction for normal distribution or with Mann‒Whitney rank-sum test for non-normal distribution data. **∗***P* < 0.05, ∗∗*P* < 0.01, ∗∗∗*P* < 0.001. AD, atopic dermatitis; HC, healthy control.

We wondered whether the infant’s age has an effect on gene expression in the blood of patients with AD in the first year of life. Previous studies have shown that differences in skin microbiome depend on infant’s age in healthy infants (Capone et al., 2011) and AD infants (Nakamura et al., 2020). To elucidate the effect of age, we stratified patients with AD and healthy controls accordingly (0‒6 months and 7‒12 months) and performed differential expression analysis on RNA-seq data. Table 3 summarizes the DEGs between four groups: patients with AD aged >6 months (n = 5) versus age-matched healthy controls (n = 2), patients with AD aged <6 months (n = 3) versus age-matched healthy controls (n = 3), patients with AD aged >6 months versus patients with AD aged <6 months, and healthy controls aged >6 months versus healthy controls aged <6 months. Interestingly, four DEGs that have been identified between AD infants and healthy controls and validated by RT-qPCR in this study (*IL1B*, *TNF*, *TREM1*, and *EGR3*) were differentially expressed in patients with AD aged <6 months compared with those in age-matched healthy controls, suggesting its unique differential expression in the first 6 months of life in patients with AD. *IL18RAP* has been shown to be differentially expressed in both age groups between patients with AD and healthy controls, suggesting no effect of age stratification on this DEG in patients with AD (Table 4). Data were further validated by RT-qPCR (Figure 3a-e). Four DEGs (*IL1B*, *TNF*, *TREM1*, and *IL18RAP*) showed significant differential expression affected by age in accordance with RNA-seq results. Expression of *EGR3* showed a trend to be affected by age; however, it was not significant as shown by RNA-seq analysis. Altogether, these data indicate that identified DEGs in patients with AD in the first year of life could be affected by age; however, more samples are required to approve this effect.

**Table 3:** Effect of Infant’s Age on Gene Expression in Infants with AD and HCs

Gene	Log2FC	FC	*P*-Value	FDR
AD aged >6 mo (n = 5) versus HCs (n = 2)				
MTCO3P12	‒8.823	0.002	5.674E-09	9.209E-05
IGKV1-16	‒3.584	0.083	4.496E-06	6.483E-03
GLDC	‒2.244	0.211	1.331E-06	3.086E-03
IL18RAP	‒1.968	0.256	1.241E-06	3.086E-03
MIF-AS1	‒1.916	0.265	1.954E-05	1.885E-02
WASHC1	‒1.739	0.300	1.974E-05	1.885E-02
SNHG22	‒1.500	0.354	7.604E-05	4.255E-02
ENSG00000273295	‒1.294	0.408	5.821E-07	2.362E-03
ENSG00000260404	‒1.045	0.485	4.011E-06	6.483E-03
KLRF1	‒1.011	0.496	2.685E-05	2.294E-02
ITGB1	0.883	1.844	8.051E-05	4.355E-02
H2AC6	1.538	2.904	9.485E-05	4.966E-02
PRKAR2B	2.157	4.460	5.766E-05	3.743E-02
GNG11	2.278	4.849	3.485E-05	2.507E-02
PEAR1	2.424	5.366	6.523E-05	4.072E-02
MPIG6B	2.499	5.651	1.643E-05	1.885E-02
PF4	2.520	5.738	7.597E-05	4.255E-02
FAXDC2	2.540	5.816	5.347E-05	3.616E-02
CAVIN2	2.585	6.000	8.349E-06	1.042E-02
PPBP	2.589	6.018	3.512E-06	6.483E-03
ITGB3	2.595	6.043	3.553E-05	2.507E-02
TUBB1	2.612	6.112	3.955E-06	6.483E-03
TREML1	2.710	6.542	2.979E-05	2.417E-02
FPR1	3.011	8.060	2.393E-05	2.158E-02
CLEC4F	3.292	9.798	1.938E-07	1.048E-03
ABLIM3	3.372	10.355	3.510E-05	2.507E-02
FPR2	3.438	10.836	7.061E-05	4.244E-02
LIN7A	4.010	16.113	4.794E-06	6.483E-03
SEC14L5	4.348	20.367	8.718E-07	2.830E-03
DDX11L10	6.851	115.408	1.897E-05	1.885E-02
IGHV5-10-1	9.633	793.981	2.177E-08	1.767E-04
AD aged <6 mo (n = 3) versus HCs (n = 3)				
ENSG00000271993	‒6.451	0.011	1.429E-04	6.675E-03
LOC107987373	‒6.102	0.015	9.330E-04	2.534E-02
ENSG00000254851	‒4.136	0.057	7.154E-04	2.125E-02
ENSG00000266302	‒4.021	0.062	3.886E-04	1.392E-02
GPR82	‒3.736	0.075	3.149E-04	1.211E-02
ENSG00000244167	‒3.326	0.100	1.761E-05	1.365E-03
GAPDHP1	‒3.207	0.108	7.216E-04	2.133E-02
CHL1	‒3.046	0.121	7.483E-04	2.173E-02
SDC1	‒2.950	0.129	5.232E-04	1.712E-02
RAVER2	‒2.943	0.130	4.456E-05	2.817E-03
FCGR3B	‒2.920	0.132	6.584E-05	3.805E-03
SLC4A10	‒2.904	0.134	2.548E-06	2.678E-04
ZBED2	‒2.888	0.135	2.211E-03	4.666E-02
SSPN	‒2.633	0.161	4.028E-04	1.426E-02
LOC730101	‒2.581	0.167	2.129E-03	4.537E-02
GLDC	‒2.464	0.181	2.327E-05	1.709E-03
IGLV2-18	‒2.420	0.187	3.579E-05	2.360E-03
IGHV3-13	‒2.394	0.190	5.888E-04	1.865E-02
IGHV3-21	‒2.390	0.191	1.176E-12	7.064E-10
CHAC2	‒2.389	0.191	2.231E-05	1.660E-03
IGKV3D-20	‒2.308	0.202	3.317E-04	1.251E-02
TNFRSF17	‒2.299	0.203	1.029E-06	1.254E-04
CDC20	‒2.240	0.212	4.534E-09	1.230E-06
IGHV3-15	‒2.202	0.217	3.022E-08	5.775E-06
MIR3142HG	‒2.184	0.220	8.995E-05	4.727E-03
LINC01355	‒2.181	0.220	3.300E-04	1.249E-02
ENSG00000230521	‒2.172	0.222	1.578E-04	7.192E-03
BHLHE41	‒2.121	0.230	1.844E-03	4.116E-02
BHLHA15	‒2.112	0.231	8.746E-04	2.427E-02
PPP1R17	‒2.103	0.233	2.441E-04	1.004E-02
OR2A9P	‒2.084	0.236	2.116E-03	4.522E-02
ENSG00000275481	‒2.066	0.239	1.494E-04	6.923E-03
PARS2	‒2.045	0.242	2.013E-03	4.369E-02
EOMES	‒2.011	0.248	1.357E-04	6.399E-03
IGHGP	‒1.983	0.253	8.716E-04	2.426E-02
JCHAIN	‒1.982	0.253	1.711E-06	1.918E-04
IGHV3-72	‒1.981	0.253	7.772E-05	4.244E-03
CISH	‒1.930	0.262	7.131E-08	1.187E-05
CD180	‒1.920	0.264	1.204E-03	3.039E-02
MCM10	‒1.919	0.264	6.847E-05	3.890E-03
LINC02273	‒1.918	0.265	1.005E-04	5.058E-03
KLRC4	‒1.911	0.266	8.811E-08	1.398E-05
IGKV1-6	‒1.891	0.270	7.272E-04	2.138E-02
TRGV8	‒1.860	0.276	1.861E-03	4.134E-02
IGLV3-19	‒1.857	0.276	8.468E-08	1.356E-05
SLC23A3	‒1.846	0.278	1.713E-03	3.930E-02
C1orf220	‒1.844	0.279	8.631E-04	2.407E-02
IGKV1-17	‒1.841	0.279	9.117E-07	1.136E-04
IGHG1	‒1.835	0.280	9.853E-04	2.625E-02
ENSG00000258810	‒1.826	0.282	1.423E-03	3.409E-02
TRGV10	‒1.785	0.290	1.548E-03	3.615E-02
IGHV3-11	‒1.780	0.291	2.487E-04	1.016E-02
FASLG	‒1.779	0.291	1.466E-05	1.174E-03
NCAPG	‒1.772	0.293	2.036E-04	8.800E-03
LINC01560	‒1.768	0.294	6.509E-04	1.979E-02
ZNF781	‒1.755	0.296	4.745E-04	1.622E-02
IGHV1-18	‒1.754	0.297	3.218E-05	2.164E-03
IGHJ3	‒1.740	0.299	5.973E-04	1.886E-02
TRAV4	‒1.727	0.302	1.912E-03	4.220E-02
IGKV1D-8	‒1.722	0.303	1.633E-04	7.322E-03
TRGC2	‒1.708	0.306	7.025E-09	1.773E-06
CUTALP	‒1.700	0.308	2.636E-04	1.060E-02
IGLV1-44	‒1.699	0.308	8.700E-10	2.703E-07
AKAP6	‒1.688	0.310	9.866E-04	2.625E-02
IGLV1-51	‒1.669	0.314	8.440E-09	1.999E-06
ZNF181	‒1.667	0.315	1.396E-05	1.123E-03
GIMAP4	‒1.663	0.316	1.074E-06	1.281E-04
KLRK1	‒1.654	0.318	1.775E-07	2.608E-05
SAMD9	‒1.637	0.321	1.956E-06	2.156E-04
MMACHC	‒1.624	0.324	7.667E-05	4.227E-03
IGKV5-2	‒1.617	0.326	7.368E-04	2.155E-02
THNSL1	‒1.600	0.330	1.009E-03	2.665E-02
PDE4DIPP6	‒1.599	0.330	3.369E-07	4.644E-05
ZNF658	‒1.595	0.331	7.522E-05	4.176E-03
GIMAP7	‒1.586	0.333	1.304E-03	3.220E-02
ZNF816	‒1.571	0.337	1.156E-07	1.817E-05
ZNF772	‒1.571	0.337	7.636E-09	1.834E-06
IGLV3-25	‒1.563	0.338	1.939E-03	4.269E-02
ZNF501	‒1.561	0.339	3.145E-04	1.211E-02
ENSG00000272913	‒1.554	0.340	3.354E-04	1.260E-02
MOCS2-DT	‒1.552	0.341	2.297E-03	4.756E-02
SAMD9L	‒1.545	0.343	9.236E-04	2.521E-02
ZNF404	‒1.543	0.343	4.938E-04	1.657E-02
ZNF470	‒1.539	0.344	8.973E-05	4.727E-03
TAS2R4	‒1.523	0.348	8.426E-04	2.381E-02
ENSG00000279696	‒1.519	0.349	1.312E-05	1.061E-03
PAQR8	‒1.516	0.350	1.029E-06	1.254E-04
ZNF594	‒1.502	0.353	5.013E-04	1.666E-02
LINC02397	‒1.500	0.354	2.194E-05	1.654E-03
IGLV8-61	‒1.496	0.355	1.777E-07	2.608E-05
TIGD7	‒1.494	0.355	2.569E-06	2.683E-04
IGKV1-39	‒1.490	0.356	8.397E-06	7.511E-04
MOXD1	‒1.490	0.356	8.500E-04	2.386E-02
CEP19	‒1.480	0.358	2.381E-03	4.866E-02
KLRD1	‒1.475	0.360	4.971E-04	1.662E-02
FRMPD3	‒1.465	0.362	1.696E-05	1.326E-03
IGKV1-9	‒1.457	0.364	1.158E-04	5.679E-03
ZNF780A	‒1.443	0.368	1.961E-06	2.156E-04
RTP4	‒1.434	0.370	7.186E-04	2.128E-02
IGLV2-8	‒1.433	0.370	5.396E-05	3.288E-03
BTN3A2	‒1.431	0.371	1.402E-06	1.626E-04
TMEM60	‒1.414	0.375	7.592E-04	2.192E-02
FAM111A-DT	‒1.389	0.382	3.412E-04	1.272E-02
ENSG00000279267	‒1.387	0.382	2.038E-03	4.405E-02
SLAMF7	‒1.384	0.383	2.446E-03	4.961E-02
GEMIN6	‒1.375	0.386	1.182E-06	1.399E-04
IGKV2-30	‒1.374	0.386	1.834E-04	8.071E-03
CASP4LP	‒1.370	0.387	2.150E-03	4.565E-02
JRKL	‒1.370	0.387	4.453E-04	1.535E-02
BTLA	‒1.366	0.388	3.303E-06	3.306E-04
ZNNT1	‒1.364	0.388	1.516E-06	1.734E-04
ENSG00000246596	‒1.363	0.389	2.840E-04	1.121E-02
UBE2T	‒1.361	0.389	1.501E-04	6.935E-03
ZBTB32	‒1.356	0.391	2.469E-03	4.990E-02
EEF1AKNMT	‒1.356	0.391	2.746E-05	1.938E-03
IGHV3-33	‒1.355	0.391	1.341E-04	6.372E-03
ENSG00000232611	‒1.353	0.392	2.330E-03	4.784E-02
CXCR3	‒1.350	0.392	3.158E-04	1.211E-02
CD200	‒1.349	0.393	1.689E-04	7.535E-03
IGKV4-1	‒1.347	0.393	9.343E-04	2.534E-02
DTX3L	‒1.342	0.394	5.186E-04	1.703E-02
IGHV1-46	‒1.339	0.395	6.659E-05	3.835E-03
GCSAM	‒1.337	0.396	3.056E-05	2.096E-03
ZNF66	‒1.325	0.399	5.140E-04	1.691E-02
GIMAP1	‒1.324	0.399	7.225E-09	1.773E-06
AURKA	‒1.324	0.400	6.992E-04	2.096E-02
CENPBD1	‒1.322	0.400	2.913E-04	1.144E-02
IL18RAP	‒1.309	0.404	1.963E-03	4.305E-02
NA	‒1.304	0.405	2.527E-09	7.081E-07
ZNF737	‒1.302	0.406	5.127E-04	1.690E-02
IGLV2-23	‒1.300	0.406	3.615E-04	1.324E-02
IGLV3-27	‒1.298	0.407	1.261E-03	3.136E-02
PGBD2	‒1.296	0.407	8.355E-06	7.511E-04
IGKV1-12	‒1.292	0.409	5.242E-07	6.996E-05
IGKV1-16	‒1.291	0.409	1.932E-05	1.477E-03
DENND2D	‒1.283	0.411	3.314E-05	2.202E-03
IGKV3-11	‒1.281	0.412	3.948E-07	5.354E-05
NA	‒1.279	0.412	9.526E-04	2.571E-02
CMTR2	‒1.273	0.414	7.274E-08	1.199E-05
BBS10	‒1.266	0.416	1.207E-03	3.041E-02
FIGNL1	‒1.265	0.416	1.068E-06	1.281E-04
IGLV3-1	‒1.264	0.416	9.491E-04	2.566E-02
ARSK	‒1.263	0.417	4.091E-05	2.626E-03
IGLV1-47	‒1.258	0.418	8.770E-04	2.430E-02
ZKSCAN7	‒1.242	0.423	8.287E-04	2.354E-02
IGKV3-15	‒1.240	0.423	4.808E-05	2.994E-03
HCP5	‒1.238	0.424	2.807E-05	1.966E-03
TMED2-DT	‒1.231	0.426	1.984E-03	4.328E-02
C14orf119	‒1.227	0.427	6.280E-04	1.941E-02
ZFP3	‒1.218	0.430	5.653E-05	3.383E-03
ZNF583	‒1.216	0.430	5.982E-04	1.886E-02
IGLC2	‒1.212	0.432	1.360E-03	3.330E-02
TMEM140	‒1.207	0.433	1.390E-03	3.373E-02
ZNF613	‒1.202	0.435	1.454E-03	3.459E-02
ACKR3	‒1.202	0.435	1.373E-04	6.451E-03
ZNF780B	‒1.201	0.435	3.300E-04	1.249E-02
IGKV3-20	‒1.198	0.436	1.620E-04	7.318E-03
ZNF626	‒1.191	0.438	1.015E-03	2.674E-02
ZNF607	‒1.184	0.440	1.295E-05	1.052E-03
ZNF175	‒1.181	0.441	2.765E-06	2.853E-04
LRIF1	‒1.178	0.442	2.817E-04	1.117E-02
STAT1	‒1.175	0.443	1.256E-03	3.134E-02
TRGC1	‒1.175	0.443	7.599E-05	4.203E-03
RBM12B	‒1.174	0.443	7.139E-06	6.560E-04
IGLV2-11	‒1.166	0.446	1.560E-03	3.629E-02
GIMAP6	‒1.163	0.447	2.190E-04	9.323E-03
IGKV1-5	‒1.159	0.448	1.131E-05	9.558E-04
MRPL35	‒1.159	0.448	1.822E-05	1.399E-03
ZNF721	‒1.157	0.449	2.130E-07	3.088E-05
ZNF799	‒1.155	0.449	1.154E-03	2.937E-02
CARD8-AS1	‒1.154	0.449	6.959E-04	2.090E-02
ZNF665	‒1.152	0.450	3.676E-04	1.338E-02
ZNF226	‒1.150	0.450	5.285E-09	1.411E-06
ZNF616	‒1.147	0.451	1.652E-03	3.821E-02
LXN	‒1.145	0.452	6.716E-04	2.028E-02
IGLV4-69	‒1.138	0.455	1.009E-03	2.665E-02
C5orf51	‒1.132	0.456	1.835E-03	4.104E-02
FCRL3	‒1.127	0.458	2.841E-04	1.121E-02
MYBL1	‒1.127	0.458	9.251E-04	2.521E-02
TIGD2	‒1.118	0.461	4.773E-04	1.627E-02
IL18R1	‒1.117	0.461	1.725E-03	3.947E-02
LBH	‒1.117	0.461	4.585E-05	2.866E-03
ENSG00000279059	‒1.114	0.462	1.704E-03	3.916E-02
ENSG00000259877	‒1.113	0.462	8.156E-04	2.324E-02
ZNF189	‒1.100	0.467	6.862E-04	2.068E-02
C17orf80	‒1.096	0.468	9.718E-06	8.380E-04
PREPL	‒1.095	0.468	5.271E-04	1.721E-02
ZKSCAN3	‒1.094	0.468	2.583E-04	1.047E-02
OGFOD1	‒1.094	0.468	1.696E-04	7.547E-03
ZBTB38	‒1.090	0.470	1.106E-03	2.849E-02
N6AMT1	‒1.087	0.471	8.993E-04	2.471E-02
NAPEPLD	‒1.084	0.472	1.109E-03	2.853E-02
INTS5	‒1.080	0.473	4.717E-06	4.533E-04
IGKC	‒1.079	0.473	7.877E-04	2.253E-02
GIMAP8	‒1.076	0.474	9.667E-06	8.379E-04
ABCB1	‒1.073	0.475	7.390E-04	2.158E-02
TRDC	‒1.073	0.475	2.020E-03	4.378E-02
ZNF230	‒1.072	0.476	9.037E-05	4.734E-03
UMPS	‒1.071	0.476	2.068E-05	1.574E-03
ZNF681	‒1.067	0.477	2.312E-03	4.767E-02
SLC25A20	‒1.067	0.477	1.269E-05	1.036E-03
RNASEL	‒1.063	0.479	1.762E-03	4.014E-02
MCM8	‒1.062	0.479	9.754E-05	5.016E-03
PCDH9	‒1.059	0.480	5.797E-04	1.839E-02
MRPL50	‒1.055	0.481	2.564E-04	1.042E-02
HIBCH	‒1.048	0.484	6.758E-06	6.245E-04
HERPUD2-AS1	‒1.043	0.485	1.263E-03	3.137E-02
LOC101927151	‒1.040	0.486	1.764E-03	4.014E-02
SLAMF6	‒1.038	0.487	9.397E-06	8.187E-04
L3MBTL3	‒1.023	0.492	1.079E-04	5.384E-03
SP4	‒1.022	0.492	1.445E-03	3.448E-02
ZNF397	‒1.019	0.494	2.382E-07	3.409E-05
WEE1	‒1.017	0.494	3.585E-04	1.319E-02
PURA	‒1.015	0.495	1.806E-05	1.393E-03
ENSG00000259820	‒1.010	0.497	1.386E-03	3.367E-02
TTC9C	‒1.006	0.498	4.920E-05	3.031E-03
IGLV2-14	‒1.003	0.499	4.237E-04	1.478E-02
SIT1	‒1.001	0.500	6.669E-04	2.017E-02
CHURC1	‒1.000	0.500	7.051E-04	2.104E-02
ZNF234	‒0.996	0.501	6.351E-04	1.956E-02
UTP14C	‒0.995	0.502	2.641E-04	1.060E-02
GTF2E1	‒0.994	0.502	4.997E-04	1.666E-02
LPAR6	‒0.983	0.506	6.115E-04	1.909E-02
CEACAM1	‒0.981	0.507	7.600E-04	2.192E-02
GSDMB	‒0.980	0.507	2.299E-04	9.642E-03
PM20D2	‒0.974	0.509	4.898E-05	3.028E-03
ALDH5A1	‒0.969	0.511	1.091E-03	2.826E-02
CPT1A	‒0.965	0.512	4.082E-04	1.439E-02
KCTD11	‒0.963	0.513	2.092E-04	8.998E-03
DHFR2	‒0.962	0.513	4.011E-04	1.423E-02
YIPF4	‒0.961	0.514	5.389E-06	5.063E-04
ENSG00000260719	‒0.957	0.515	1.277E-04	6.170E-03
C21orf91	‒0.954	0.516	3.112E-04	1.211E-02
TRNT1	‒0.948	0.518	2.113E-04	9.062E-03
TMEM186	‒0.946	0.519	9.766E-04	2.615E-02
SPDL1	‒0.939	0.522	9.564E-04	2.577E-02
ZFP82	‒0.919	0.529	8.385E-04	2.374E-02
DARS2	‒0.918	0.529	1.614E-03	3.744E-02
PAXIP1-AS2	‒0.906	0.534	8.944E-04	2.466E-02
MRM1	‒0.895	0.538	5.773E-04	1.839E-02
PYGO2	‒0.891	0.539	3.044E-05	2.096E-03
IRF4	‒0.888	0.540	2.427E-03	4.942E-02
ZNF486	‒0.888	0.540	6.370E-04	1.958E-02
PRAG1	‒0.888	0.540	7.519E-04	2.176E-02
ZNF320	‒0.887	0.541	9.588E-04	2.580E-02
GGPS1	‒0.883	0.542	1.508E-05	1.202E-03
TRMT13	‒0.875	0.545	7.919E-05	4.282E-03
ZNF420	‒0.873	0.546	1.863E-03	4.134E-02
FBXO22	‒0.872	0.546	3.694E-05	2.398E-03
ENSG00000268027	‒0.866	0.549	1.188E-03	3.010E-02
NUP43	‒0.865	0.549	1.418E-03	3.406E-02
TIA1	‒0.862	0.550	1.475E-06	1.699E-04
ZNF785	‒0.862	0.550	2.190E-03	4.638E-02
ZNF480	‒0.861	0.550	4.853E-05	3.011E-03
ZNF260	‒0.856	0.552	2.416E-03	4.924E-02
MAT2B	‒0.853	0.554	1.065E-05	9.093E-04
RAB29	‒0.845	0.557	4.962E-04	1.662E-02
LEO1	‒0.845	0.557	5.540E-05	3.327E-03
GIMAP2	‒0.844	0.557	1.041E-03	2.714E-02
ZNF74	‒0.843	0.557	1.478E-03	3.501E-02
ZNF200	‒0.836	0.560	2.650E-05	1.896E-03
FGFR1OP2	‒0.833	0.561	3.555E-05	2.354E-03
PABIR1	‒0.833	0.561	9.817E-04	2.620E-02
KLHL9	‒0.829	0.563	1.518E-03	3.569E-02
HSPA8	‒0.828	0.563	3.539E-04	1.308E-02
THAP5	‒0.828	0.563	1.208E-03	3.041E-02
PPIL1	‒0.824	0.565	2.268E-03	4.732E-02
FANCF	‒0.817	0.568	1.850E-03	4.121E-02
STT3A	‒0.810	0.570	2.334E-04	9.667E-03
VKORC1L1	‒0.800	0.574	2.362E-03	4.832E-02
CTR9	‒0.800	0.574	8.579E-04	2.400E-02
UBE4A	‒0.799	0.575	1.130E-04	5.605E-03
IRF1-AS1	‒0.797	0.576	2.316E-03	4.767E-02
RBM4B	‒0.795	0.576	5.011E-04	1.666E-02
KCTD21	‒0.795	0.576	2.442E-03	4.961E-02
RPE	‒0.793	0.577	4.809E-04	1.630E-02
UBA6-AS1	‒0.787	0.580	1.551E-04	7.125E-03
POLH	‒0.778	0.583	2.054E-03	4.417E-02
TMEM223	‒0.775	0.584	1.512E-03	3.561E-02
ZNF561	‒0.772	0.586	1.749E-03	3.995E-02
TRIM4	‒0.769	0.587	7.730E-05	4.234E-03
ZNF557	‒0.765	0.589	2.316E-03	4.767E-02
FCMR	‒0.762	0.590	2.407E-06	2.546E-04
ZKSCAN1	‒0.759	0.591	4.671E-04	1.600E-02
ENSG00000239665	‒0.757	0.592	5.955E-05	3.526E-03
EIF2S1	‒0.752	0.594	6.725E-05	3.860E-03
C15orf40	‒0.751	0.594	6.441E-04	1.966E-02
ZNF154	‒0.749	0.595	6.572E-04	1.995E-02
BRD8	‒0.748	0.595	2.083E-05	1.578E-03
ARCN1	‒0.748	0.595	1.313E-04	6.280E-03
ERGIC2	‒0.741	0.598	6.239E-04	1.935E-02
FASTKD1	‒0.737	0.600	7.642E-04	2.200E-02
ZNF146	‒0.736	0.600	1.282E-03	3.172E-02
ZNF841	‒0.736	0.601	2.956E-04	1.159E-02
TRIM27	‒0.736	0.601	1.358E-04	6.399E-03
KRBOX4	‒0.729	0.603	3.577E-04	1.319E-02
HSPH1	‒0.713	0.610	1.531E-03	3.587E-02
NCOR1	‒0.713	0.610	1.901E-03	4.202E-02
HNRNPF	‒0.713	0.610	1.906E-04	8.346E-03
COL19A1	‒0.699	0.616	2.265E-03	4.732E-02
MON2	‒0.686	0.622	2.158E-03	4.576E-02
MITD1	‒0.684	0.622	3.227E-04	1.233E-02
ATAD2B	‒0.679	0.624	1.235E-03	3.092E-02
TUBGCP4	‒0.676	0.626	5.429E-04	1.762E-02
MRPS14	‒0.674	0.627	2.343E-03	4.805E-02
GLS	‒0.673	0.627	1.256E-03	3.134E-02
VEZT	‒0.668	0.629	2.260E-03	4.732E-02
TARS1	‒0.653	0.636	1.282E-04	6.179E-03
PIK3R4	‒0.641	0.641	2.285E-03	4.740E-02
OBI1	‒0.641	0.641	2.286E-03	4.740E-02
ZNF671	‒0.635	0.644	1.380E-03	3.359E-02
LINC00667	‒0.635	0.644	6.179E-04	1.924E-02
MCM3	‒0.626	0.648	3.529E-04	1.307E-02
PIGF	‒0.612	0.654	2.230E-03	4.694E-02
THRAP3	‒0.604	0.658	1.783E-04	7.869E-03
PLRG1	‒0.599	0.660	2.278E-03	4.736E-02
APOL2	‒0.596	0.662	1.351E-03	3.318E-02
GART	‒0.591	0.664	2.004E-03	4.359E-02
NUP42	‒0.588	0.665	1.143E-03	2.922E-02
ZNF559	‒0.587	0.666	8.986E-04	2.471E-02
ZNF362	0.588	1.503	2.457E-03	4.978E-02
WBP11	0.591	1.506	1.572E-03	3.652E-02
MAPRE1	0.593	1.509	1.099E-03	2.834E-02
INSIG1	0.599	1.515	1.037E-03	2.711E-02
CCNY	0.602	1.517	7.865E-04	2.253E-02
JARID2	0.621	1.537	1.219E-03	3.059E-02
FBRS	0.622	1.539	3.948E-04	1.404E-02
MAPK1IP1L	0.625	1.542	1.989E-03	4.332E-02
RBM3	0.629	1.547	1.531E-03	3.587E-02
CSGALNACT2	0.632	1.549	1.552E-03	3.615E-02
INTS1	0.646	1.565	1.496E-03	3.529E-02
HNRNPH2	0.649	1.568	3.683E-04	1.338E-02
TMEM167B	0.655	1.575	3.365E-04	1.260E-02
LAPTM5	0.655	1.575	4.076E-04	1.439E-02
MLF2	0.660	1.581	1.411E-03	3.403E-02
CHCHD2	0.660	1.581	1.233E-03	3.090E-02
RREB1	0.661	1.581	9.899E-05	5.044E-03
TMCC1	0.663	1.584	2.444E-03	4.961E-02
EME2	0.664	1.584	6.241E-04	1.935E-02
TRA2B	0.664	1.584	8.481E-04	2.385E-02
RAB1A	0.669	1.590	4.183E-04	1.469E-02
UBC	0.676	1.597	5.208E-05	3.196E-03
NCOR2	0.677	1.599	1.943E-03	4.272E-02
DAZAP2	0.677	1.599	5.074E-04	1.680E-02
ARF1	0.681	1.603	8.186E-05	4.398E-03
ADIPOR1	0.685	1.608	9.716E-05	5.012E-03
EIF5A	0.685	1.608	1.589E-04	7.222E-03
MKNK2	0.688	1.611	1.371E-03	3.345E-02
RAPGEF2	0.691	1.614	4.612E-04	1.583E-02
HNRNPA0	0.691	1.614	1.675E-03	3.854E-02
TET3	0.692	1.615	1.974E-03	4.317E-02
HNRNPL	0.693	1.617	1.246E-05	1.022E-03
MAP1LC3B	0.694	1.618	8.722E-05	4.627E-03
RPS9	0.707	1.633	2.099E-03	4.490E-02
RHBDD2	0.711	1.637	1.781E-03	4.030E-02
SLC25A3	0.715	1.641	6.243E-05	3.645E-03
SYF2	0.719	1.646	1.787E-03	4.033E-02
OTULINL	0.726	1.654	8.728E-04	2.426E-02
ENSG00000279117	0.734	1.664	1.777E-03	4.028E-02
RNF26	0.746	1.677	9.815E-04	2.620E-02
PAFAH1B2	0.748	1.680	1.165E-04	5.695E-03
HERPUD1	0.750	1.682	1.949E-04	8.511E-03
RSL24D1	0.752	1.684	1.096E-03	2.830E-02
MCL1	0.752	1.685	1.803E-03	4.054E-02
MRFAP1	0.755	1.688	1.623E-04	7.318E-03
PNRC1	0.762	1.696	8.879E-04	2.452E-02
SUMO3	0.765	1.700	2.090E-04	8.998E-03
BUD31	0.776	1.712	1.796E-03	4.049E-02
SLC25A33	0.790	1.729	1.775E-03	4.028E-02
COX4I1	0.792	1.732	3.267E-04	1.246E-02
ANXA1	0.793	1.732	7.262E-04	2.138E-02
AP1G2-AS1	0.796	1.736	1.838E-04	8.071E-03
SRF	0.798	1.739	1.130E-03	2.892E-02
MMP24OS	0.799	1.739	1.058E-04	5.309E-03
CYP4V2	0.799	1.740	1.630E-04	7.322E-03
BCL7B	0.801	1.743	3.883E-04	1.392E-02
PNPLA2	0.803	1.745	6.248E-04	1.935E-02
TGIF1	0.809	1.752	7.705E-05	4.234E-03
SNN	0.811	1.754	2.741E-04	1.095E-02
UAP1L1	0.819	1.764	7.464E-04	2.172E-02
EEPD1	0.831	1.778	3.783E-04	1.368E-02
TRAF4	0.847	1.798	4.790E-04	1.627E-02
CARD19	0.849	1.801	5.571E-04	1.789E-02
TUBB4B	0.850	1.802	1.602E-05	1.264E-03
ADGRL1-AS1	0.863	1.818	1.448E-03	3.448E-02
CLK1	0.869	1.826	2.140E-03	4.555E-02
IRF1	0.870	1.828	9.341E-06	8.187E-04
SLC3A2	0.876	1.835	1.523E-04	7.017E-03
RNF19B	0.876	1.836	1.819E-03	4.085E-02
IDI1	0.876	1.836	4.200E-06	4.154E-04
KLHL26	0.888	1.850	4.394E-04	1.522E-02
VAMP3	0.888	1.850	3.411E-04	1.272E-02
ETF1	0.889	1.852	9.769E-07	1.208E-04
AKT1S1	0.892	1.856	2.312E-05	1.705E-03
MAPK7	0.892	1.856	2.053E-03	4.417E-02
CDKN2D	0.893	1.857	3.615E-05	2.374E-03
EIF1B	0.894	1.859	8.468E-04	2.385E-02
SNIP1	0.895	1.859	1.426E-04	6.675E-03
TMEM201	0.896	1.861	4.790E-04	1.627E-02
DDX21	0.902	1.869	3.152E-05	2.138E-03
NECAP1	0.902	1.869	1.724E-06	1.920E-04
VPS18	0.905	1.873	6.841E-05	3.890E-03
RFX2	0.910	1.879	1.151E-03	2.932E-02
MEF2D	0.911	1.881	8.729E-09	2.039E-06
SIVA1	0.915	1.886	2.287E-05	1.694E-03
ATXN1L	0.915	1.886	6.338E-06	5.888E-04
ANAPC15	0.924	1.897	2.563E-04	1.042E-02
CDK2AP1	0.930	1.905	1.783E-03	4.030E-02
MED30	0.930	1.905	4.214E-04	1.473E-02
PRNP	0.930	1.906	1.267E-04	6.138E-03
CDKN1B	0.932	1.907	1.403E-03	3.389E-02
TENT5C	0.933	1.909	4.399E-04	1.522E-02
PI4K2A	0.937	1.914	3.144E-04	1.211E-02
ENSG00000280138	0.939	1.917	9.366E-06	8.187E-04
TUBA1C	0.941	1.920	1.147E-04	5.658E-03
UPP1	0.945	1.925	2.448E-04	1.004E-02
DENND1A	0.946	1.926	2.786E-04	1.110E-02
GCNT2	0.948	1.929	1.830E-03	4.097E-02
SELENOK	0.953	1.936	5.480E-05	3.305E-03
PTS	0.964	1.951	3.127E-04	1.211E-02
RBM7	0.974	1.964	2.460E-03	4.979E-02
PRXL2C	0.980	1.973	1.258E-03	3.134E-02
TWF1	0.986	1.980	1.977E-04	8.612E-03
ATF4	0.990	1.987	3.687E-05	2.398E-03
ENSG00000269688	0.993	1.991	3.432E-04	1.277E-02
WDR74	1.000	2.000	5.483E-05	3.305E-03
GPR35	1.001	2.001	1.053E-06	1.274E-04
RNF103	1.004	2.006	5.484E-04	1.770E-02
EMD	1.005	2.008	2.369E-05	1.725E-03
MGST3	1.014	2.020	5.682E-04	1.816E-02
JMJD6	1.015	2.021	7.918E-05	4.282E-03
GRPEL1	1.016	2.023	2.361E-06	2.513E-04
CCRL2	1.018	2.025	1.023E-03	2.693E-02
CTIF	1.019	2.026	8.366E-04	2.372E-02
NFKBIB	1.024	2.034	3.801E-04	1.372E-02
MNT	1.033	2.046	7.009E-05	3.955E-03
TOM1	1.042	2.059	1.475E-03	3.497E-02
TLE3	1.044	2.062	3.236E-06	3.258E-04
JOSD1	1.044	2.062	3.044E-04	1.190E-02
FCGRT	1.046	2.065	1.830E-03	4.097E-02
EIF5	1.048	2.068	2.865E-08	5.537E-06
NXT1	1.050	2.071	1.717E-05	1.336E-03
ARHGDIA	1.052	2.073	3.600E-04	1.322E-02
BRI3	1.054	2.076	2.099E-03	4.490E-02
NDUFV2	1.060	2.085	9.459E-04	2.561E-02
CLN8-AS1	1.063	2.090	2.094E-03	4.490E-02
FEM1C	1.065	2.092	2.259E-04	9.522E-03
NR1H2	1.067	2.095	3.494E-04	1.297E-02
FASTKD5	1.092	2.131	1.315E-04	6.280E-03
YOD1	1.093	2.134	1.716E-03	3.931E-02
PTGER4	1.096	2.138	2.812E-06	2.884E-04
SMURF1	1.097	2.139	4.288E-06	4.168E-04
CCNL1	1.102	2.147	5.947E-09	1.562E-06
LDLR	1.106	2.152	3.636E-05	2.379E-03
SLC22A18	1.107	2.154	2.273E-03	4.736E-02
IRGQ	1.115	2.166	1.200E-03	3.035E-02
CHASERR	1.116	2.167	1.599E-04	7.247E-03
HMGB3	1.118	2.170	2.043E-03	4.409E-02
IER5	1.119	2.172	1.609E-06	1.816E-04
EIF2AK3-DT	1.120	2.174	2.237E-03	4.703E-02
TUBB2A	1.122	2.177	7.424E-04	2.164E-02
PDE4B	1.123	2.178	1.433E-04	6.675E-03
ADAM9	1.127	2.184	5.438E-04	1.762E-02
GPR183	1.127	2.184	1.572E-06	1.786E-04
CKAP4	1.128	2.185	1.157E-04	5.679E-03
KLF11	1.128	2.186	3.067E-05	2.096E-03
ZMIZ1	1.129	2.187	9.055E-04	2.480E-02
RBM34	1.132	2.192	1.973E-03	4.317E-02
KCNQ1	1.132	2.192	5.875E-07	7.599E-05
GDPD5	1.136	2.197	6.395E-04	1.959E-02
C6orf226	1.136	2.198	7.171E-04	2.127E-02
MFSD2A	1.137	2.199	1.225E-04	5.954E-03
BACH1	1.140	2.204	2.817E-04	1.117E-02
MYLIP	1.143	2.209	9.289E-06	8.187E-04
RELL1	1.151	2.220	5.583E-04	1.789E-02
EIF1	1.159	2.234	1.296E-07	2.017E-05
SLC7A5	1.160	2.234	1.919E-03	4.230E-02
ATP6V0C	1.168	2.247	3.262E-05	2.177E-03
YRDC	1.169	2.249	6.586E-08	1.119E-05
DDX47	1.173	2.254	1.445E-03	3.448E-02
CPNE2	1.173	2.255	2.342E-05	1.712E-03
CCDC59	1.176	2.260	2.328E-04	9.667E-03
ENSG00000258944	1.181	2.267	2.202E-03	4.652E-02
H3-3B	1.182	2.269	2.639E-04	1.060E-02
GPX1	1.182	2.270	1.148E-03	2.930E-02
ZFAND2A	1.187	2.277	2.136E-06	2.333E-04
RAPH1	1.198	2.294	1.846E-03	4.116E-02
CBX4	1.204	2.304	2.524E-10	9.430E-08
PRR7	1.219	2.328	2.186E-04	9.323E-03
MAFK	1.221	2.332	6.495E-08	1.119E-05
KIAA1522	1.227	2.340	7.294E-05	4.075E-03
FTH1	1.227	2.341	1.395E-03	3.380E-02
GABARAPL1	1.229	2.344	6.533E-08	1.119E-05
PELI1	1.232	2.349	4.744E-08	8.398E-06
IRF2BP2	1.236	2.356	2.639E-11	1.082E-08
ENSG00000255847	1.243	2.367	2.218E-05	1.658E-03
SULT1A1	1.245	2.370	2.045E-03	4.409E-02
KLF6	1.245	2.371	2.311E-04	9.642E-03
DUS3L	1.255	2.387	1.237E-05	1.020E-03
PIGA	1.265	2.404	1.580E-05	1.253E-03
TUBA1A	1.269	2.410	4.847E-04	1.640E-02
RNF139	1.270	2.412	9.661E-04	2.595E-02
OAF	1.272	2.415	1.378E-03	3.358E-02
ADCY9	1.284	2.435	3.922E-04	1.397E-02
TIPARP	1.293	2.451	7.424E-06	6.748E-04
CEBPB	1.296	2.456	2.245E-03	4.711E-02
ENSG00000262766	1.307	2.474	2.312E-03	4.767E-02
PTPRE	1.316	2.490	6.628E-04	2.008E-02
PMAIP1	1.317	2.492	6.762E-07	8.680E-05
NRROS	1.319	2.495	9.700E-05	5.012E-03
CBX6	1.319	2.495	1.784E-07	2.608E-05
BCL10	1.322	2.499	1.566E-08	3.292E-06
RIPK2	1.323	2.502	1.029E-03	2.698E-02
YPEL5	1.324	2.504	2.780E-07	3.895E-05
SIAH1	1.328	2.511	2.318E-04	9.648E-03
RELT	1.329	2.513	1.116E-03	2.863E-02
NA	1.330	2.513	2.400E-03	4.897E-02
MAFG	1.331	2.515	1.962E-08	3.966E-06
POLR1F	1.337	2.527	5.113E-06	4.858E-04
ZBTB21	1.342	2.535	5.950E-05	3.526E-03
GZF1	1.342	2.535	4.131E-10	1.478E-07
MCEMP1	1.347	2.543	1.265E-03	3.137E-02
SNHG15	1.357	2.561	3.176E-10	1.161E-07
EIF4A1	1.357	2.561	9.866E-09	2.242E-06
C5AR1	1.374	2.592	1.495E-03	3.529E-02
FBXO33	1.377	2.598	1.552E-03	3.615E-02
PLPPR2	1.384	2.611	5.988E-04	1.886E-02
ZNF628	1.389	2.619	6.461E-05	3.759E-03
OSER1	1.392	2.625	1.015E-08	2.277E-06
SIRPA	1.395	2.630	1.520E-03	3.571E-02
KDM6B	1.404	2.646	3.228E-08	6.099E-06
SRGN	1.408	2.653	7.075E-05	3.979E-03
DGCR11	1.413	2.663	2.472E-03	4.990E-02
HNRNPD-DT	1.414	2.665	8.446E-04	2.383E-02
SPATA2	1.416	2.668	7.274E-09	1.773E-06
CD69	1.416	2.669	4.169E-04	1.466E-02
RBM38	1.425	2.685	1.195E-05	9.982E-04
ZNF394	1.426	2.688	3.509E-08	6.485E-06
ANKRD9	1.427	2.690	5.212E-04	1.708E-02
RBM47	1.431	2.696	3.906E-04	1.394E-02
B3GNT7	1.433	2.700	2.744E-05	1.938E-03
EAF1	1.438	2.709	3.380E-14	2.842E-11
PTP4A1	1.442	2.717	4.180E-07	5.624E-05
MXD1	1.447	2.727	3.820E-04	1.376E-02
BNIP1	1.448	2.729	5.154E-06	4.869E-04
ETS2	1.449	2.729	5.042E-04	1.672E-02
HIF1A	1.457	2.745	9.755E-09	2.242E-06
TOE1	1.468	2.766	4.288E-05	2.731E-03
CLP1	1.468	2.766	8.330E-08	1.347E-05
ENSG00000260708	1.477	2.783	6.458E-04	1.967E-02
DUSP5	1.477	2.783	5.683E-11	2.209E-08
C17orf49	1.479	2.787	5.141E-10	1.729E-07
MYADM	1.491	2.810	3.961E-06	3.941E-04
SERTAD1	1.491	2.811	5.429E-06	5.072E-04
SERTAD3	1.498	2.825	7.674E-08	1.253E-05
TTC34	1.499	2.826	3.359E-04	1.260E-02
NFKBID	1.499	2.826	1.296E-04	6.227E-03
ENSG00000279602	1.499	2.827	4.282E-06	4.168E-04
LDLRAD3	1.516	2.861	2.247E-03	4.711E-02
CRABP2	1.524	2.877	1.358E-03	3.330E-02
ZP3	1.525	2.878	2.169E-04	9.279E-03
SMARCD3	1.525	2.878	1.459E-03	3.466E-02
MMP14	1.527	2.882	2.038E-03	4.405E-02
SDE2	1.533	2.893	4.053E-08	7.328E-06
GLT1D1	1.536	2.900	1.483E-03	3.506E-02
F5	1.539	2.907	1.117E-03	2.863E-02
H2BC21	1.547	2.921	4.282E-04	1.491E-02
CEACAM19	1.547	2.923	2.489E-04	1.016E-02
ENSG00000277879	1.551	2.930	1.364E-03	3.334E-02
DUSP1	1.554	2.936	3.121E-04	1.211E-02
ARRDC4	1.555	2.938	1.240E-08	2.707E-06
OTUD1	1.561	2.950	9.380E-05	4.868E-03
STX11	1.567	2.963	9.889E-05	5.044E-03
RNVU1-30	1.568	2.966	8.028E-04	2.292E-02
VENTX	1.576	2.981	2.028E-04	8.796E-03
JDP2	1.578	2.986	5.716E-07	7.451E-05
SOX4	1.591	3.013	4.929E-04	1.657E-02
RBM38-AS1	1.594	3.018	1.567E-04	7.159E-03
HIC1	1.595	3.020	4.478E-06	4.328E-04
BTG3	1.596	3.024	7.075E-04	2.106E-02
LINC00963	1.597	3.025	1.758E-03	4.012E-02
NRGN	1.599	3.029	1.192E-04	5.809E-03
CXCR4	1.600	3.032	5.429E-08	9.511E-06
UBE2FP1	1.607	3.047	8.372E-05	4.469E-03
DUSP10	1.612	3.056	5.420E-05	3.290E-03
MTSS2	1.615	3.064	8.435E-10	2.676E-07
STAB1	1.617	3.068	1.627E-03	3.768E-02
LONRF3	1.619	3.071	1.771E-04	7.835E-03
IER5L	1.625	3.084	4.887E-06	4.669E-04
SH3RF1	1.630	3.096	3.304E-04	1.249E-02
NIBAN2	1.653	3.144	1.078E-04	5.384E-03
SBDS	1.653	3.146	5.381E-07	7.069E-05
ZC3H12A	1.654	3.148	1.620E-13	1.048E-10
LOC102723996	1.667	3.175	1.768E-03	4.018E-02
TNRC18P1	1.675	3.194	7.768E-04	2.229E-02
FAM20C	1.692	3.232	3.151E-04	1.211E-02
JUND	1.705	3.261	4.856E-10	1.701E-07
CSF3R	1.718	3.291	1.421E-03	3.409E-02
BTG2	1.727	3.310	1.556E-04	7.131E-03
AHR	1.735	3.328	5.842E-05	3.483E-03
GGTA1	1.740	3.341	2.319E-03	4.767E-02
SKOR1	1.742	3.346	1.901E-03	4.202E-02
GADD45A	1.743	3.347	1.157E-05	9.729E-04
MIR222HG	1.747	3.356	9.930E-05	5.045E-03
MIDN	1.749	3.361	4.288E-05	2.731E-03
ZBTB10	1.750	3.363	1.332E-04	6.346E-03
KLF10	1.753	3.371	2.696E-04	1.079E-02
RAB3A	1.758	3.383	7.505E-04	2.176E-02
HES1	1.759	3.384	4.456E-04	1.535E-02
SNORA75	1.760	3.388	1.416E-03	3.406E-02
FSCN1	1.760	3.388	4.913E-04	1.656E-02
ENSG00000275056	1.761	3.390	2.029E-04	8.796E-03
JAG1	1.768	3.405	8.961E-05	4.727E-03
GNA15	1.785	3.446	2.928E-06	2.966E-04
ADM	1.791	3.461	6.120E-04	1.909E-02
MGP	1.792	3.463	2.357E-03	4.828E-02
JUNB	1.800	3.482	1.334E-08	2.841E-06
NINJ1	1.804	3.492	3.508E-08	6.485E-06
CCR1	1.810	3.507	3.162E-04	1.211E-02
COL9A3	1.812	3.510	1.799E-03	4.050E-02
ENSG00000280067	1.814	3.516	9.222E-04	2.521E-02
RPL32P1	1.820	3.532	8.565E-04	2.400E-02
GAS2L1	1.832	3.560	3.706E-04	1.343E-02
NFKBIE	1.850	3.604	1.032E-08	2.283E-06
NOCT	1.851	3.607	2.854E-05	1.991E-03
SMOX	1.852	3.610	5.454E-04	1.764E-02
SLC6A8	1.859	3.628	1.896E-03	4.200E-02
RGS2	1.860	3.630	1.027E-05	8.808E-04
ENSG00000272256	1.876	3.670	8.199E-04	2.333E-02
S100A8	1.880	3.681	3.006E-05	2.080E-03
LMNA	1.891	3.710	4.578E-08	8.190E-06
KLF4	1.906	3.748	3.816E-05	2.459E-03
JUN	1.909	3.754	1.266E-08	2.729E-06
PDGFC	1.912	3.764	1.164E-03	2.952E-02
EREG	1.914	3.769	3.635E-04	1.328E-02
SLC22A4	1.916	3.775	1.061E-03	2.763E-02
ITPRIP	1.918	3.778	4.358E-14	3.490E-11
BHLHE40	1.918	3.780	1.442E-07	2.204E-05
CDKN1A	1.920	3.784	2.221E-06	2.395E-04
GADD45B	1.925	3.797	1.663E-08	3.410E-06
IER2	1.926	3.801	1.071E-05	9.093E-04
RETN	1.937	3.828	4.254E-06	4.168E-04
P2RY2	1.943	3.846	1.003E-03	2.657E-02
CD14	1.953	3.872	2.367E-04	9.780E-03
TNFAIP3	1.956	3.881	4.405E-05	2.795E-03
RASGEF1B	1.958	3.885	6.554E-05	3.801E-03
VEGFA	1.967	3.909	2.005E-08	3.966E-06
DDIT3	1.973	3.925	3.717E-15	4.167E-12
ENSG00000232811	1.978	3.940	2.881E-06	2.937E-04
LRG1	1.979	3.943	1.737E-04	7.707E-03
ARHGAP22	1.979	3.943	6.293E-04	1.942E-02
IGHEP2	1.988	3.966	5.389E-04	1.756E-02
ENSG00000279520	1.994	3.984	4.254E-16	5.503E-13
RHBDL1	2.002	4.005	7.004E-04	2.096E-02
BTBD19	2.014	4.040	2.229E-04	9.444E-03
HOMER3	2.017	4.047	9.268E-05	4.825E-03
ENC1	2.022	4.063	2.181E-06	2.366E-04
ENSG00000273972	2.030	4.084	1.666E-03	3.837E-02
RGS17P1	2.048	4.135	1.199E-05	9.982E-04
S100A9	2.049	4.137	8.698E-06	7.739E-04
FOS	2.056	4.159	7.651E-07	9.674E-05
ENSG00000270640	2.067	4.190	1.267E-03	3.137E-02
SNAI1	2.070	4.200	1.332E-03	3.280E-02
LRP3	2.087	4.249	6.814E-05	3.890E-03
KCNK7	2.089	4.253	3.744E-05	2.421E-03
IFITM10	2.095	4.273	3.236E-05	2.168E-03
NECTIN2	2.118	4.342	3.293E-04	1.249E-02
SCN1B	2.129	4.374	7.524E-05	4.176E-03
S100A12	2.131	4.380	7.126E-05	3.994E-03
NRARP	2.152	4.443	2.014E-03	4.369E-02
IRS2	2.154	4.451	6.444E-10	2.125E-07
DUSP2	2.158	4.462	9.808E-15	9.461E-12
MAFB	2.167	4.491	3.263E-07	4.535E-05
ZFP36	2.168	4.493	2.525E-09	7.081E-07
NFKBIA	2.170	4.500	1.211E-11	5.817E-09
NR6A1	2.176	4.518	8.261E-05	4.424E-03
ENSG00000207525	2.182	4.539	6.065E-04	1.906E-02
FOSL2	2.187	4.552	1.416E-11	6.582E-09
AREG	2.189	4.561	5.583E-04	1.789E-02
PPP1R15A	2.196	4.583	8.779E-14	5.905E-11
ICAM1	2.198	4.587	3.686E-12	2.066E-09
SOCS3	2.198	4.587	2.672E-06	2.774E-04
MIR616	2.208	4.619	1.664E-03	3.837E-02
SLC2A3	2.209	4.623	1.570E-18	2.934E-15
PLAU	2.209	4.624	2.676E-05	1.907E-03
DIP2A-IT1	2.209	4.625	4.566E-05	2.865E-03
ENSG00000266993	2.215	4.642	2.278E-03	4.736E-02
PPIF	2.227	4.683	1.967E-12	1.141E-09
NA	2.248	4.749	2.364E-08	4.622E-06
NAMPT	2.259	4.786	4.009E-08	7.328E-06
DUSP6	2.259	4.788	1.487E-07	2.253E-05
ENSG00000256913	2.261	4.793	1.039E-03	2.712E-02
ENSG00000275210	2.272	4.830	9.994E-04	2.651E-02
IFI30	2.282	4.863	9.844E-05	5.044E-03
PIM3	2.285	4.874	1.380E-25	5.800E-22
LGALS12	2.286	4.876	3.171E-05	2.141E-03
ASGR2	2.290	4.890	2.306E-04	9.642E-03
ANPEP	2.291	4.892	1.736E-07	2.606E-05
FFAR1	2.292	4.896	1.448E-11	6.582E-09
CSRNP1	2.299	4.923	5.836E-14	4.267E-11
NFIL3	2.304	4.939	7.050E-08	1.185E-05
TCF3P1	2.307	4.948	9.014E-04	2.473E-02
HLX	2.344	5.077	1.339E-03	3.291E-02
PLK3	2.346	5.085	2.505E-25	8.425E-22
PDE2A	2.351	5.103	2.590E-04	1.047E-02
PFKFB3	2.356	5.121	1.178E-17	1.981E-14
SOWAHC	2.363	5.143	6.411E-04	1.960E-02
TNFRSF12A	2.374	5.183	6.012E-05	3.547E-03
MIR22HG	2.378	5.198	2.038E-09	5.908E-07
XK	2.387	5.230	1.413E-03	3.403E-02
BCL3	2.393	5.254	6.803E-12	3.690E-09
ENSG00000268734	2.402	5.284	3.668E-07	5.015E-05
GADD45G	2.415	5.332	1.857E-03	4.131E-02
ENSG00000274008	2.423	5.363	6.394E-04	1.959E-02
ASTL	2.435	5.409	1.479E-04	6.872E-03
ELOVL7	2.469	5.537	2.200E-03	4.652E-02
MATN1	2.469	5.537	4.546E-04	1.563E-02
TRIB1	2.476	5.562	2.206E-05	1.656E-03
CHRM4	2.507	5.683	7.352E-04	2.154E-02
ENSG00000250138	2.514	5.711	8.650E-05	4.603E-03
ATP2B1-AS1	2.520	5.735	2.634E-05	1.896E-03
PLAUR	2.555	5.875	5.065E-10	1.729E-07
TSPEAR-AS1	2.582	5.989	1.314E-03	3.239E-02
GP9	2.589	6.018	2.218E-03	4.674E-02
AVPI1	2.591	6.023	5.787E-04	1.839E-02
RND1	2.601	6.068	8.633E-04	2.407E-02
PTX3	2.618	6.140	7.139E-12	3.752E-09
NLRP3	2.625	6.168	1.345E-07	2.075E-05
MMP25	2.630	6.191	2.027E-14	1.794E-11
IER3	2.659	6.315	8.094E-13	5.041E-10
FAM238A	2.669	6.359	1.596E-11	6.884E-09
RBKS	2.677	6.395	2.392E-07	3.409E-05
EMP1	2.694	6.471	5.779E-11	2.209E-08
DUSP8	2.700	6.500	2.755E-05	1.938E-03
RRAD	2.718	6.581	5.245E-05	3.207E-03
MIR23AHG	2.733	6.646	6.241E-15	6.559E-12
RAB20	2.739	6.675	8.601E-14	5.905E-11
NA	2.741	6.684	7.517E-17	1.149E-13
MAFF	2.742	6.690	1.013E-14	9.461E-12
TMEM88	2.744	6.700	1.217E-05	1.008E-03
MMP9	2.766	6.802	7.058E-04	2.104E-02
PHLDA1	2.802	6.973	2.759E-07	3.895E-05
CHMP4BP1	2.806	6.992	2.993E-05	2.080E-03
NR4A1	2.843	7.176	2.237E-06	2.396E-04
IGFBP2	2.853	7.226	1.002E-04	5.058E-03
ENSG00000234436	2.893	7.426	5.720E-04	1.825E-02
ENSG00000274677	2.893	7.426	1.663E-04	7.437E-03
PER1	2.898	7.452	1.029E-25	5.765E-22
ENSG00000278022	2.940	7.677	9.969E-05	5.050E-03
ATF3	2.943	7.691	4.281E-11	1.714E-08
NTSR1	2.954	7.747	7.847E-06	7.094E-04
SLC22A16	2.984	7.913	1.026E-03	2.696E-02
TP53INP2	2.990	7.943	1.099E-26	9.240E-23
TMEM119	3.002	8.008	1.083E-03	2.809E-02
CCL2	3.020	8.110	2.218E-04	9.420E-03
THNSL2	3.021	8.116	2.647E-05	1.896E-03
RNVU1-3	3.027	8.148	7.264E-04	2.138E-02
ENSG00000270681	3.038	8.212	7.719E-04	2.219E-02
PANX2	3.042	8.235	6.241E-05	3.645E-03
TAMALIN	3.044	8.249	1.567E-16	2.196E-13
TNFSF9	3.078	8.446	1.045E-11	5.287E-09
NRIP3	3.082	8.467	4.518E-05	2.845E-03
TNF	3.092	8.526	8.842E-10	2.703E-07
MT1XP1	3.114	8.655	1.089E-04	5.419E-03
FCAR	3.125	8.726	8.222E-07	1.032E-04
ENSG00000274051	3.136	8.791	6.113E-04	1.909E-02
PDGFA-DT	3.137	8.797	1.983E-03	4.328E-02
KRT86	3.148	8.866	1.644E-05	1.291E-03
LOC399900	3.149	8.873	3.641E-04	1.328E-02
TREM1	3.164	8.963	7.190E-10	2.325E-07
MIR4420	3.198	9.176	5.616E-14	4.267E-11
ENSG00000224356	3.202	9.202	7.259E-06	6.634E-04
FOSB	3.211	9.260	1.335E-09	4.009E-07
OSM	3.260	9.582	1.623E-08	3.370E-06
C17orf107	3.312	9.931	8.022E-05	4.324E-03
HP	3.333	10.078	9.749E-04	2.615E-02
RNU5D-1	3.342	10.140	1.076E-03	2.797E-02
CD83	3.385	10.448	1.852E-33	3.114E-29
CXCL2	3.402	10.573	1.213E-06	1.427E-04
HBEGF	3.505	11.350	1.069E-11	5.287E-09
SGK1	3.516	11.443	1.492E-11	6.602E-09
B3GNT5	3.536	11.600	7.350E-07	9.363E-05
NR4A3	3.586	12.011	1.649E-11	6.932E-09
NR4A2	3.622	12.313	6.717E-23	1.883E-19
FAM71A	3.657	12.618	5.086E-04	1.680E-02
ZNF503	3.707	13.055	6.415E-09	1.659E-06
LRRC32	3.721	13.190	7.182E-09	1.773E-06
ENSG00000280407	3.756	13.514	2.566E-05	1.860E-03
GJB6	3.764	13.585	2.250E-04	9.505E-03
PHLDA2	3.834	14.263	5.363E-07	7.069E-05
THBS1	3.897	14.896	2.312E-03	4.767E-02
IL1B	3.901	14.942	2.202E-15	2.645E-12
SEMA6B	3.922	15.161	1.147E-04	5.658E-03
RNVU1-6	3.981	15.794	1.348E-04	6.387E-03
RNF152	4.010	16.115	2.091E-03	4.490E-02
SPX	4.028	16.315	1.538E-03	3.597E-02
SNORD3B-2	4.092	17.048	2.855E-04	1.124E-02
ENSG00000218809	4.104	17.192	1.987E-08	3.966E-06
RN7SL368P	4.609	24.404	7.879E-05	4.282E-03
TEX45	4.751	26.919	6.112E-04	1.909E-02
CCL3L3	4.768	27.241	5.399E-04	1.756E-02
EGR1	4.840	28.632	2.278E-04	9.579E-03
G0S2	4.981	31.578	5.628E-20	1.352E-16
CXCL8	4.995	31.887	9.194E-05	4.801E-03
EGR3	5.076	33.732	4.196E-04	1.470E-02
ADRA2B	5.110	34.527	2.412E-04	9.943E-03
LINC01220	5.216	37.168	6.991E-05	3.955E-03
SLED1	5.246	37.958	1.372E-06	1.603E-04
ID1	5.485	44.786	4.129E-09	1.138E-06
ENSG00000224029	5.501	45.302	4.341E-04	1.508E-02
EGR2	5.788	55.266	3.139E-05	2.137E-03
MMP2-AS1	6.120	69.545	1.158E-03	2.943E-02
ENSG00000258413	6.192	73.113	1.445E-03	3.448E-02
FOSL1	6.357	81.941	7.520E-19	1.581E-15
CXCL1	6.439	86.742	7.316E-04	2.147E-02
LERFS	6.597	96.823	4.893E-04	1.652E-02
ENSG00000261026	6.791	110.769	6.213E-05	3.645E-03
CLLU1-AS1	8.387	334.831	1.475E-09	4.350E-07
AD aged >6 mo (n = 5) versus AD aged <6 mo (n = 3)				
WASHC1	‒2.328	0.199	3.255E-10	5.279E-06
MYO18B	‒4.947	0.032	2.603E-06	1.477E-02
IFI27	‒3.795	0.072	2.732E-06	1.477E-02
NRIR	‒2.206	0.217	8.777E-06	3.558E-02
HCs aged >6 mo (n = 2) versus HCs aged <6 mo (n = 3)				
ENSG00000213058	‒7.336	0.006	8.210E-06	3.863E-04
RPS14P1	‒6.971	0.008	4.300E-05	1.468E-03
RPL23P3	‒6.413	0.012	5.931E-04	1.061E-02
CLEC4F	‒5.654	0.020	2.039E-03	2.629E-02
FCGR3B	‒4.753	0.037	4.584E-03	4.626E-02
IFIT3	‒4.526	0.043	6.902E-04	1.182E-02
AGAP14P	‒4.423	0.047	2.570E-05	9.886E-04
NAALAD2	‒4.269	0.052	3.906E-03	4.164E-02
IFIT1	‒4.029	0.061	3.432E-03	3.804E-02
P2RY12	‒4.015	0.062	4.380E-04	8.525E-03
ENSG00000254851	‒3.978	0.063	2.595E-03	3.152E-02
EFHB	‒3.961	0.064	1.762E-03	2.363E-02
PFN1P1	‒3.950	0.065	3.605E-03	3.958E-02
CXCL10	‒3.923	0.066	3.740E-07	2.960E-05
L1TD1	‒3.846	0.070	3.650E-08	3.750E-06
FPR2	‒3.678	0.078	4.660E-13	1.850E-10
GPR20	‒3.606	0.082	5.070E-03	4.963E-02
LINC02432	‒3.506	0.088	8.400E-07	5.800E-05
ENSG00000276758	‒3.502	0.088	2.538E-03	3.100E-02
ANKRD22	‒3.458	0.091	6.360E-06	3.128E-04
ZBED2	‒3.352	0.098	2.091E-04	5.014E-03
P2RY13	‒3.293	0.102	2.920E-10	5.790E-08
PPP1R17	‒3.272	0.104	1.290E-08	1.500E-06
NAGS	‒3.192	0.109	2.293E-03	2.863E-02
FAM20A	‒3.164	0.112	2.420E-07	2.090E-05
FILIP1L	‒3.149	0.113	5.340E-04	9.756E-03
ASH2LP1	‒3.135	0.114	4.320E-08	4.420E-06
LINC01506	‒3.090	0.117	1.024E-03	1.591E-02
CCDC121	‒3.043	0.121	1.007E-03	1.572E-02
NDN	‒2.991	0.126	1.018E-03	1.585E-02
CX3CR1	‒2.971	0.128	1.589E-04	4.006E-03
SCARA5	‒2.957	0.129	9.880E-04	1.550E-02
GPR82	‒2.940	0.130	3.116E-03	3.563E-02
RAVER2	‒2.931	0.131	4.369E-03	4.490E-02
C3AR1	‒2.920	0.132	7.920E-12	2.400E-09
SFTPD	‒2.851	0.139	1.863E-03	2.459E-02
GATA2	‒2.842	0.139	4.630E-04	8.797E-03
CYP4F22	‒2.805	0.143	1.490E-06	9.340E-05
PTGDR2	‒2.782	0.145	3.070E-14	1.600E-11
NA	‒2.742	0.149	2.330E-07	2.020E-05
OLFML2B	‒2.707	0.153	2.643E-03	3.192E-02
OAS1	‒2.704	0.153	9.279E-04	1.478E-02
CXCR1	‒2.653	0.159	2.508E-04	5.733E-03
TLR8	‒2.651	0.159	1.510E-09	2.310E-07
NLRC4	‒2.642	0.160	3.610E-06	2.014E-04
CCR2	‒2.640	0.160	9.070E-13	3.360E-10
S1PR3	‒2.621	0.163	2.945E-03	3.436E-02
CD180	‒2.618	0.163	5.740E-18	5.030E-15
ENSG00000261655	‒2.597	0.165	1.793E-04	4.432E-03
RET	‒2.594	0.166	6.200E-05	1.931E-03
TMEM51	‒2.583	0.167	3.675E-03	4.002E-02
HGF	‒2.582	0.167	6.970E-05	2.100E-03
IGSF6	‒2.544	0.171	3.770E-07	2.960E-05
XAF1	‒2.542	0.172	4.601E-03	4.632E-02
ENSG00000244167	‒2.540	0.172	5.150E-05	1.696E-03
CISH	‒2.536	0.172	7.870E-16	4.860E-13
SIGLEC5	‒2.509	0.176	2.174E-04	5.166E-03
C3	‒2.503	0.176	5.271E-04	9.661E-03
MOCS2-DT	‒2.497	0.177	2.500E-06	1.454E-04
TNFSF10	‒2.480	0.179	3.380E-20	4.340E-17
CEACAM3	‒2.413	0.188	5.356E-04	9.773E-03
AATBC	‒2.413	0.188	2.713E-03	3.243E-02
KCNE3	‒2.388	0.191	1.140E-05	5.160E-04
PRICKLE1	‒2.382	0.192	8.130E-07	5.640E-05
HSPA1A	‒2.329	0.199	2.020E-11	5.360E-09
HECW2	‒2.328	0.199	3.273E-03	3.677E-02
PDK4	‒2.317	0.201	2.060E-10	4.460E-08
DAZL	‒2.287	0.205	3.266E-03	3.675E-02
CDKN1C	‒2.230	0.213	2.010E-19	2.230E-16
CARD6	‒2.187	0.220	2.910E-11	7.450E-09
ICAM4	‒2.168	0.222	2.367E-04	5.508E-03
MYOF	‒2.148	0.226	3.050E-06	1.735E-04
FPR1	‒2.123	0.230	2.210E-26	5.830E-23
FGL2	‒2.122	0.230	3.672E-03	4.001E-02
LILRA6	‒2.119	0.230	1.780E-05	7.353E-04
TLR10	‒2.117	0.231	2.050E-11	5.360E-09
CCR1	‒2.092	0.235	2.990E-08	3.170E-06
RGS18	‒2.089	0.235	4.505E-04	8.648E-03
CXCR2	‒2.082	0.236	3.500E-05	1.250E-03
GTF2H2	‒2.073	0.238	1.280E-07	1.210E-05
FZD1	‒2.061	0.240	4.600E-05	1.547E-03
BATF2	‒2.057	0.240	3.069E-03	3.531E-02
SOWAHD	‒2.056	0.241	1.090E-06	7.290E-05
IRAG1	‒2.052	0.241	4.760E-07	3.590E-05
MIR5195	‒2.035	0.244	6.010E-06	2.991E-04
CDC42EP2	‒2.033	0.244	5.500E-05	1.775E-03
ENSG00000227615	‒2.030	0.245	6.680E-10	1.140E-07
GPBAR1	‒2.010	0.248	4.630E-09	6.270E-07
OAS2	‒2.009	0.248	4.692E-03	4.699E-02
LINC01504	‒1.998	0.250	6.430E-05	1.983E-03
LRRC25	‒1.991	0.252	1.420E-08	1.630E-06
SLC31A2	‒1.987	0.252	3.510E-07	2.810E-05
SENCR	‒1.986	0.252	3.747E-03	4.041E-02
PELATON	‒1.984	0.253	1.100E-11	3.110E-09
GAPT	‒1.978	0.254	2.060E-11	5.360E-09
IGHV5-78	‒1.977	0.254	1.280E-08	1.500E-06
TMEM60	‒1.973	0.255	1.690E-09	2.550E-07
ROR1	‒1.962	0.257	3.031E-03	3.500E-02
TLR7	‒1.961	0.257	2.450E-08	2.670E-06
FFAR2	‒1.938	0.261	9.490E-05	2.687E-03
HSPA6	‒1.938	0.261	3.690E-04	7.515E-03
FCGR3A	‒1.937	0.261	1.270E-13	5.880E-11
NFE2	‒1.908	0.266	1.430E-08	1.640E-06
FNDC5	‒1.900	0.268	1.033E-04	2.882E-03
SAMD9L	‒1.876	0.273	1.471E-03	2.076E-02
GEMIN6	‒1.873	0.273	5.100E-07	3.780E-05
FMNL2	‒1.871	0.273	1.701E-04	4.248E-03
IGLV3-27	‒1.864	0.275	1.210E-05	5.409E-04
PRSS30P	‒1.864	0.275	1.497E-03	2.106E-02
MS4A7	‒1.862	0.275	3.310E-08	3.460E-06
GIMAP4	‒1.853	0.277	1.070E-26	3.560E-23
SAMD9	‒1.848	0.278	4.260E-10	7.880E-08
FZD2	‒1.845	0.278	3.042E-03	3.507E-02
ISL2	‒1.841	0.279	4.605E-03	4.633E-02
LINC02576	‒1.838	0.280	3.872E-03	4.140E-02
ENSG00000257275	‒1.817	0.284	4.550E-07	3.460E-05
ERAP2	‒1.808	0.286	7.980E-32	6.650E-28
CCDC126	‒1.803	0.287	3.840E-05	1.343E-03
TASL	‒1.801	0.287	3.530E-14	1.780E-11
SOS1-IT1	‒1.799	0.287	5.103E-04	9.447E-03
AQP9	‒1.797	0.288	3.120E-05	1.147E-03
LOC105377623	‒1.790	0.289	2.519E-03	3.082E-02
UGGT2	‒1.754	0.296	1.282E-03	1.885E-02
CD24	‒1.748	0.298	8.600E-09	1.090E-06
FKBPL	‒1.747	0.298	1.998E-04	4.838E-03
PLA2G2D	‒1.745	0.298	1.614E-03	2.228E-02
OLIG1	‒1.737	0.300	9.447E-04	1.503E-02
KLK1	‒1.735	0.300	4.544E-03	4.608E-02
TCL1A	‒1.731	0.301	3.480E-10	6.590E-08
PARS2	‒1.729	0.302	7.571E-04	1.266E-02
ENSG00000261222	‒1.720	0.303	5.548E-04	1.010E-02
MTMR11	‒1.714	0.305	2.341E-04	5.470E-03
CALHM2	‒1.713	0.305	1.930E-09	2.870E-07
MIR223HG	‒1.712	0.305	8.440E-14	4.020E-11
MSR1	‒1.710	0.306	4.511E-03	4.591E-02
ENSG00000279696	‒1.702	0.307	4.450E-06	2.370E-04
MGC16275	‒1.696	0.309	1.449E-03	2.056E-02
SMCO4	‒1.677	0.313	1.040E-06	6.980E-05
GCNT1	‒1.664	0.315	1.510E-13	6.640E-11
GUCY1B1	‒1.657	0.317	2.921E-03	3.417E-02
SPTA1	‒1.648	0.319	5.100E-03	4.983E-02
CEBPA	‒1.638	0.321	1.121E-04	3.076E-03
LINC01355	‒1.635	0.322	2.380E-10	4.970E-08
CKB	‒1.632	0.323	5.940E-05	1.885E-03
SLAMF8	‒1.618	0.326	1.039E-03	1.606E-02
LPCAT2	‒1.614	0.327	2.918E-03	3.417E-02
HS3ST1	‒1.594	0.331	1.160E-07	1.110E-05
C9orf64	‒1.583	0.334	5.090E-06	2.633E-04
C1orf220	‒1.583	0.334	8.730E-06	4.051E-04
TRIM69	‒1.581	0.334	6.100E-07	4.400E-05
B3GNT8	‒1.580	0.334	2.682E-03	3.222E-02
KCNQ1OT1	‒1.575	0.336	1.056E-04	2.932E-03
DTX3L	‒1.568	0.337	1.530E-05	6.538E-04
FCRLB	‒1.566	0.338	1.747E-03	2.346E-02
SECTM1	‒1.563	0.339	6.220E-05	1.935E-03
LMO2	‒1.553	0.341	6.500E-05	1.997E-03
C14orf119	‒1.553	0.341	1.010E-15	6.010E-13
ENSG00000279611	‒1.546	0.343	4.701E-03	4.702E-02
HMOX1	‒1.543	0.343	1.680E-05	7.022E-04
ADPRH	‒1.538	0.344	8.100E-06	3.835E-04
MIR4645	‒1.537	0.345	2.188E-03	2.774E-02
ATP9A	‒1.537	0.345	9.535E-04	1.514E-02
TNFAIP8L2	‒1.533	0.346	1.380E-13	6.210E-11
TNFRSF8	‒1.532	0.346	5.600E-07	4.090E-05
CD101	‒1.532	0.346	1.404E-03	2.012E-02
LOC100287896	‒1.532	0.346	3.405E-03	3.785E-02
MAFB	‒1.528	0.347	2.330E-07	2.020E-05
IGHJ3	‒1.527	0.347	4.816E-03	4.776E-02
DUSP6	‒1.524	0.348	2.180E-09	3.180E-07
ZNF404	‒1.523	0.348	6.190E-05	1.930E-03
HECW2-AS1	‒1.521	0.348	4.760E-05	1.584E-03
CCR5	‒1.521	0.348	4.240E-07	3.270E-05
RTP4	‒1.518	0.349	6.874E-04	1.179E-02
ZNF594	‒1.514	0.350	1.640E-08	1.850E-06
LINC00324	‒1.511	0.351	9.090E-07	6.230E-05
ZNF2	‒1.507	0.352	3.583E-04	7.393E-03
CPM	‒1.504	0.353	2.150E-04	5.116E-03
LILRB3	‒1.502	0.353	7.820E-06	3.756E-04
LACTB2	‒1.501	0.353	1.611E-04	4.048E-03
TREML2	‒1.488	0.357	3.180E-07	2.580E-05
HYAL2	‒1.475	0.360	8.192E-04	1.342E-02
ABCC3	‒1.473	0.360	1.356E-03	1.967E-02
APOBEC3A	‒1.471	0.361	9.350E-05	2.667E-03
SLC37A2	‒1.456	0.364	4.837E-04	9.086E-03
FASLG	‒1.456	0.365	5.238E-04	9.622E-03
C2orf74-DT	‒1.454	0.365	7.110E-04	1.209E-02
SIGLEC9	‒1.452	0.365	6.010E-04	1.072E-02
METTL7A	‒1.451	0.366	4.190E-09	5.770E-07
MERTK	‒1.440	0.368	5.084E-04	9.422E-03
MPEG1	‒1.436	0.370	2.350E-06	1.372E-04
ZNF772	‒1.432	0.371	2.930E-08	3.130E-06
ENSG00000213976	‒1.432	0.371	2.061E-04	4.963E-03
EEF1AKNMT	‒1.431	0.371	1.190E-12	4.200E-10
FANCL	‒1.431	0.371	1.368E-03	1.974E-02
PTPN13	‒1.429	0.371	1.868E-03	2.465E-02
CARD8-AS1	‒1.423	0.373	9.660E-08	9.580E-06
EOMES	‒1.417	0.374	2.810E-05	1.047E-03
ARHGEF2-AS2	‒1.417	0.375	8.446E-04	1.370E-02
RIN2	‒1.410	0.376	6.321E-04	1.113E-02
DOK1	‒1.409	0.376	7.160E-10	1.200E-07
RPP40	‒1.408	0.377	9.037E-04	1.444E-02
IKBIP	‒1.406	0.377	9.800E-05	2.758E-03
PPIL1	‒1.403	0.378	1.960E-06	1.186E-04
IL12RB2	‒1.401	0.379	3.335E-03	3.732E-02
ZNF28	‒1.395	0.380	2.710E-10	5.540E-08
CALHM6	‒1.394	0.381	2.730E-06	1.585E-04
BATF3	‒1.392	0.381	9.785E-04	1.544E-02
PLEKHO2	‒1.387	0.382	3.450E-05	1.240E-03
CFD	‒1.386	0.383	1.290E-05	5.688E-04
PAQR8	‒1.378	0.385	1.370E-15	7.600E-13
LYL1	‒1.377	0.385	3.210E-09	4.490E-07
MME	‒1.375	0.385	1.296E-04	3.434E-03
ACKR3	‒1.374	0.386	2.048E-03	2.637E-02
ERI2	‒1.373	0.386	3.010E-03	3.486E-02
MIR3142HG	‒1.372	0.386	1.712E-03	2.314E-02
LOC100507642	‒1.370	0.387	7.370E-04	1.244E-02
PHF23	‒1.358	0.390	9.300E-13	3.370E-10
SLC1A5	‒1.354	0.391	1.930E-07	1.740E-05
FCGR2A	‒1.350	0.392	2.890E-03	3.394E-02
TOR4A	‒1.347	0.393	5.020E-07	3.740E-05
GBP1	‒1.347	0.393	7.940E-07	5.560E-05
TRMT5	‒1.336	0.396	3.285E-04	6.876E-03
ZNF583	‒1.334	0.397	7.960E-06	3.809E-04
HSD17B7P2	‒1.331	0.397	1.180E-06	7.710E-05
NEURL1	‒1.331	0.397	2.470E-07	2.110E-05
TMEM187	‒1.331	0.398	4.428E-04	8.558E-03
IRAK3	‒1.329	0.398	1.075E-03	1.652E-02
FCRLA	‒1.323	0.400	1.210E-05	5.393E-04
UBE2T	‒1.321	0.400	1.458E-03	2.065E-02
PCTP	‒1.315	0.402	5.240E-05	1.724E-03
PLP2	‒1.314	0.402	9.830E-04	1.544E-02
LILRA2	‒1.314	0.402	2.914E-03	3.416E-02
FAM111A-DT	‒1.309	0.404	2.550E-05	9.866E-04
SCIMP	‒1.304	0.405	4.207E-04	8.286E-03
HOMEZ	‒1.295	0.408	1.380E-07	1.290E-05
CEP19	‒1.295	0.408	2.026E-03	2.618E-02
ZNF613	‒1.289	0.409	3.699E-04	7.515E-03
LPAR6	‒1.288	0.410	1.960E-09	2.890E-07
SRD5A3	‒1.288	0.410	7.341E-04	1.240E-02
SLAMF7	‒1.286	0.410	1.300E-05	5.718E-04
ZNF626	‒1.284	0.411	1.163E-03	1.753E-02
DNASE1L3	‒1.283	0.411	3.735E-03	4.040E-02
STAT2	‒1.263	0.417	2.690E-05	1.015E-03
TBC1D8	‒1.261	0.417	4.760E-05	1.584E-03
CPPED1	‒1.260	0.418	2.761E-03	3.279E-02
ASCL2	‒1.254	0.419	1.172E-04	3.191E-03
LBH	‒1.253	0.420	3.350E-11	8.220E-09
CENPBD1	‒1.251	0.420	1.053E-03	1.624E-02
ANKRD50	‒1.250	0.420	2.015E-03	2.610E-02
SLFN12	‒1.243	0.423	2.490E-07	2.110E-05
ZNF181	‒1.238	0.424	3.000E-05	1.115E-03
STAT1	‒1.237	0.424	2.053E-04	4.963E-03
CLEC4C	‒1.236	0.424	8.864E-04	1.424E-02
S100A11	‒1.235	0.425	2.330E-05	9.237E-04
UGDH	‒1.232	0.426	2.366E-03	2.935E-02
TMEM102	‒1.231	0.426	1.510E-05	6.490E-04
TNFSF13B	‒1.229	0.426	4.534E-03	4.603E-02
NA	‒1.229	0.427	3.618E-04	7.403E-03
KIAA0040	‒1.223	0.428	3.210E-07	2.600E-05
MKKS	‒1.221	0.429	1.460E-07	1.350E-05
IL18	‒1.219	0.430	4.469E-03	4.562E-02
C5AR2	‒1.218	0.430	1.241E-03	1.833E-02
IGHV1-46	‒1.215	0.431	3.240E-05	1.176E-03
ARL11	‒1.214	0.431	4.450E-04	8.582E-03
INTS5	‒1.211	0.432	1.080E-07	1.050E-05
FCF1P2	‒1.210	0.432	1.403E-03	2.012E-02
APOBEC3G	‒1.207	0.433	2.488E-04	5.708E-03
ZNNT1	‒1.206	0.433	5.280E-05	1.725E-03
RMI2	‒1.202	0.435	1.260E-03	1.855E-02
WDCP	‒1.201	0.435	6.150E-06	3.038E-04
TNFSF13	‒1.201	0.435	2.655E-03	3.196E-02
DUSP18	‒1.200	0.435	5.990E-05	1.895E-03
HCK	‒1.199	0.435	4.173E-04	8.248E-03
PILRA	‒1.198	0.436	2.697E-03	3.232E-02
BBS10	‒1.198	0.436	1.015E-04	2.848E-03
ATP6AP1L	‒1.198	0.436	1.217E-03	1.813E-02
HSD17B1-AS1	‒1.196	0.436	1.510E-07	1.390E-05
POLR1G	‒1.196	0.436	1.774E-03	2.370E-02
LRRN3	‒1.195	0.437	3.750E-05	1.317E-03
USP27X	‒1.192	0.438	2.640E-04	5.947E-03
LOC101927151	‒1.191	0.438	1.292E-04	3.433E-03
TNFRSF1A	‒1.188	0.439	5.630E-07	4.090E-05
DDX60	‒1.185	0.440	3.927E-03	4.173E-02
TOB2	‒1.183	0.440	3.710E-07	2.950E-05
PECAM1	‒1.183	0.440	5.970E-10	1.030E-07
PNP	‒1.183	0.441	5.320E-06	2.736E-04
RNASEL	‒1.182	0.441	2.120E-12	6.910E-10
C1orf162	‒1.180	0.441	4.700E-10	8.410E-08
ADAMTSL4	‒1.180	0.441	7.991E-04	1.318E-02
BTN3A2	‒1.180	0.441	2.363E-04	5.506E-03
GIMAP1	‒1.178	0.442	1.960E-10	4.290E-08
JRKL	‒1.176	0.443	4.600E-05	1.547E-03
IL1RN	‒1.175	0.443	9.194E-04	1.466E-02
ZNF285	‒1.174	0.443	2.190E-05	8.742E-04
TPPP3	‒1.174	0.443	9.537E-04	1.514E-02
RNASE6	‒1.173	0.443	2.160E-14	1.160E-11
ENSG00000272335	‒1.173	0.443	2.948E-03	3.437E-02
LAP3	‒1.173	0.444	8.283E-04	1.349E-02
CD86	‒1.172	0.444	5.029E-04	9.348E-03
CASP1	‒1.172	0.444	3.550E-06	1.983E-04
LTBR	‒1.170	0.445	2.660E-03	3.200E-02
ADAP2	‒1.168	0.445	3.579E-04	7.393E-03
KLRD1	‒1.162	0.447	1.602E-03	2.224E-02
VMP1	‒1.160	0.447	7.660E-05	2.259E-03
PLEK	‒1.149	0.451	1.570E-06	9.790E-05
TRAFD1	‒1.144	0.453	6.480E-07	4.630E-05
ABI3	‒1.144	0.453	1.180E-06	7.710E-05
HAL	‒1.141	0.453	2.249E-03	2.830E-02
KCTD21	‒1.137	0.455	1.600E-05	6.781E-04
ENSG00000256448	‒1.137	0.455	1.332E-03	1.940E-02
CYBB	‒1.135	0.455	3.195E-04	6.754E-03
ENSG00000260285	‒1.133	0.456	1.768E-03	2.368E-02
FAM241A	‒1.132	0.456	5.080E-04	9.422E-03
NMI	‒1.131	0.457	2.150E-06	1.273E-04
SLC45A3	‒1.129	0.457	3.666E-03	3.999E-02
PPP1R18	‒1.127	0.458	5.600E-06	2.835E-04
HCG11	‒1.127	0.458	2.639E-04	5.947E-03
GIMAP8	‒1.124	0.459	3.390E-10	6.580E-08
FCRL5	‒1.124	0.459	6.368E-04	1.118E-02
FGD2	‒1.123	0.459	5.260E-05	1.724E-03
PTPRO	‒1.123	0.459	1.100E-08	1.330E-06
PGBD2	‒1.122	0.460	1.100E-07	1.060E-05
TPTEP1	‒1.120	0.460	4.080E-05	1.408E-03
UTP14C	‒1.116	0.461	4.080E-06	2.210E-04
INIP	‒1.115	0.462	2.610E-05	1.000E-03
UBE2L6	‒1.113	0.462	2.381E-03	2.943E-02
BTLA	‒1.111	0.463	9.090E-06	4.206E-04
APOBEC3C	‒1.109	0.464	3.420E-10	6.580E-08
ZKSCAN7	‒1.108	0.464	4.324E-04	8.457E-03
RCBTB2	‒1.108	0.464	1.027E-03	1.591E-02
ZNF470	‒1.106	0.465	2.219E-04	5.237E-03
ZNF681	‒1.105	0.465	7.397E-04	1.247E-02
CD79B	‒1.105	0.465	5.150E-06	2.657E-04
ENSG00000224376	‒1.103	0.465	3.860E-03	4.136E-02
FLVCR1-DT	‒1.103	0.466	2.981E-03	3.461E-02
KLRK1	‒1.102	0.466	2.644E-03	3.192E-02
RASSF4	‒1.102	0.466	1.567E-04	3.985E-03
CTSS	‒1.102	0.466	4.441E-03	4.547E-02
DHFR2	‒1.100	0.467	8.841E-04	1.422E-02
SPN	‒1.100	0.467	1.170E-06	7.680E-05
RALB	‒1.099	0.467	1.720E-05	7.147E-04
UICLM	‒1.098	0.467	7.470E-05	2.216E-03
LRIF1	‒1.096	0.468	4.036E-04	8.043E-03
GLRX	‒1.095	0.468	1.990E-07	1.780E-05
TUBB6	‒1.095	0.468	1.079E-04	2.991E-03
SLC29A1	‒1.095	0.468	6.040E-05	1.898E-03
CD200	‒1.094	0.469	1.800E-06	1.105E-04
FCER1G	‒1.091	0.469	1.248E-03	1.840E-02
IRF5	‒1.089	0.470	8.010E-04	1.320E-02
GNS	‒1.087	0.471	2.959E-03	3.441E-02
TCF7L2	‒1.084	0.472	6.690E-06	3.258E-04
ZNF189	‒1.084	0.472	1.020E-07	1.010E-05
CMTR2	‒1.084	0.472	1.730E-11	4.720E-09
FAM214B	‒1.083	0.472	3.420E-08	3.560E-06
MARCKS	‒1.082	0.472	4.835E-03	4.787E-02
GIMAP7	‒1.082	0.473	6.790E-05	2.063E-03
LINC01013	‒1.081	0.473	2.989E-04	6.441E-03
PUS3	‒1.078	0.474	2.080E-05	8.385E-04
SLC15A2	‒1.078	0.474	2.390E-05	9.377E-04
GCSAM	‒1.075	0.475	8.230E-09	1.050E-06
TRGC2	‒1.074	0.475	3.750E-10	7.020E-08
FAM110A	‒1.074	0.475	7.300E-10	1.210E-07
LACTB	‒1.072	0.476	2.431E-04	5.617E-03
GPATCH11	‒1.070	0.476	8.420E-07	5.800E-05
WARS1	‒1.069	0.477	1.080E-11	3.090E-09
DYNLL1	‒1.068	0.477	2.960E-06	1.686E-04
CHRNB1	‒1.068	0.477	2.403E-04	5.577E-03
FCRL3	‒1.067	0.477	2.550E-07	2.150E-05
ATP6V1D	‒1.066	0.478	1.420E-06	8.930E-05
CETN3	‒1.064	0.478	3.247E-04	6.821E-03
MRPL18	‒1.061	0.479	2.270E-07	2.000E-05
TMEM223	‒1.059	0.480	7.130E-06	3.462E-04
PYGO2	‒1.059	0.480	1.300E-08	1.500E-06
KYNU	‒1.057	0.481	3.775E-03	4.063E-02
PLSCR1	‒1.055	0.481	4.601E-03	4.632E-02
C5AR1	‒1.053	0.482	2.546E-03	3.105E-02
LST1	‒1.053	0.482	3.431E-04	7.127E-03
RNF144B	‒1.051	0.483	1.890E-05	7.709E-04
FAM220A	‒1.045	0.485	2.789E-04	6.162E-03
RIN1	‒1.043	0.485	1.947E-03	2.540E-02
CRTAM	‒1.041	0.486	1.554E-03	2.168E-02
EVI2B	‒1.040	0.486	3.140E-05	1.148E-03
LILRB1	‒1.039	0.487	1.270E-05	5.627E-04
RAB10	‒1.038	0.487	5.030E-07	3.740E-05
IL3RA	‒1.037	0.487	1.840E-03	2.437E-02
FCGR2B	‒1.035	0.488	4.392E-04	8.539E-03
DCLRE1B	‒1.033	0.489	1.202E-03	1.800E-02
SPIB	‒1.033	0.489	3.184E-03	3.606E-02
ZNF429	‒1.033	0.489	2.480E-04	5.700E-03
OPA3	‒1.031	0.489	7.460E-05	2.215E-03
GPR171	‒1.029	0.490	2.780E-06	1.602E-04
CDC42EP3	‒1.029	0.490	4.700E-03	4.702E-02
SIGLEC10	‒1.029	0.490	2.730E-10	5.540E-08
TIGD7	‒1.026	0.491	2.080E-05	8.376E-04
RNF122	‒1.024	0.492	6.710E-07	4.760E-05
SLC7A7	‒1.024	0.492	2.823E-03	3.322E-02
KIAA0930	‒1.023	0.492	1.210E-12	4.200E-10
ERLIN1	‒1.021	0.493	4.880E-06	2.555E-04
RUNDC1	‒1.021	0.493	1.790E-05	7.398E-04
ZNF616	‒1.019	0.493	8.790E-05	2.521E-03
LINC02397	‒1.019	0.494	2.840E-06	1.630E-04
FASTKD1	‒1.019	0.494	1.100E-07	1.060E-05
MED11	‒1.019	0.494	5.590E-07	4.090E-05
VPREB3	‒1.017	0.494	1.564E-03	2.180E-02
WSB1	‒1.016	0.494	1.170E-07	1.120E-05
SMIM14	‒1.015	0.495	1.700E-06	1.049E-04
LOC101929698	‒1.012	0.496	2.827E-04	6.213E-03
SAMD4A	‒1.011	0.496	6.553E-04	1.142E-02
KEAP1	‒1.009	0.497	5.880E-05	1.873E-03
MRPL35	‒1.007	0.498	6.320E-05	1.958E-03
DHRS4-AS1	‒1.006	0.498	1.310E-05	5.726E-04
NAGA	‒1.006	0.498	7.165E-04	1.217E-02
TMEM69	‒1.004	0.499	8.350E-06	3.911E-04
MED20	‒1.002	0.499	9.210E-06	4.249E-04
ZNF747-DT	‒1.000	0.500	2.951E-03	3.438E-02
NAPSB	‒0.996	0.501	6.440E-05	1.983E-03
FAM200A	‒0.996	0.501	9.017E-04	1.444E-02
SLFN11	‒0.994	0.502	8.150E-11	1.860E-08
SAC3D1	‒0.993	0.502	8.717E-04	1.405E-02
GBGT1	‒0.993	0.502	2.652E-04	5.947E-03
HERPUD2-AS1	‒0.992	0.503	1.313E-04	3.467E-03
DAPP1	‒0.991	0.503	6.790E-11	1.620E-08
TRDC	‒0.991	0.503	5.847E-04	1.052E-02
THNSL1	‒0.988	0.504	2.648E-03	3.192E-02
IFI16	‒0.986	0.505	9.310E-06	4.287E-04
POU2F2-AS1	‒0.985	0.505	4.342E-03	4.469E-02
LILRA5	‒0.984	0.506	4.738E-03	4.716E-02
LILRB2	‒0.977	0.508	3.090E-03	3.548E-02
LAT2	‒0.974	0.509	5.718E-04	1.033E-02
ALDH6A1	‒0.974	0.509	3.542E-03	3.898E-02
SLC43A3	‒0.974	0.509	6.830E-05	2.074E-03
AIF1	‒0.971	0.510	2.695E-03	3.232E-02
YWHAH	‒0.968	0.511	6.491E-04	1.134E-02
MPZL1	‒0.967	0.512	5.960E-05	1.888E-03
HCP5	‒0.965	0.512	1.300E-07	1.230E-05
PRCP	‒0.964	0.513	4.170E-05	1.428E-03
CD300LB	‒0.963	0.513	2.151E-03	2.744E-02
C6orf47	‒0.962	0.513	1.250E-05	5.549E-04
LPAR5	‒0.961	0.514	7.540E-05	2.228E-03
GLIPR1	‒0.959	0.514	1.412E-04	3.652E-03
TTC9C	‒0.958	0.515	2.160E-05	8.654E-04
ARV1	‒0.958	0.515	2.602E-04	5.899E-03
SELPLG	‒0.958	0.515	3.480E-06	1.960E-04
LINC00847	‒0.956	0.516	2.380E-05	9.366E-04
QRSL1	‒0.955	0.516	1.520E-10	3.370E-08
NCF1	‒0.953	0.516	2.739E-04	6.060E-03
RRAS	‒0.946	0.519	2.787E-03	3.295E-02
TIGD2	‒0.945	0.519	4.460E-04	8.591E-03
CYSLTR1	‒0.943	0.520	1.079E-03	1.656E-02
TNFRSF1B	‒0.941	0.521	5.580E-05	1.799E-03
IGLV2-14	‒0.939	0.521	6.289E-04	1.109E-02
MCM8	‒0.938	0.522	6.061E-04	1.079E-02
WEE1	‒0.938	0.522	4.960E-05	1.641E-03
BORCS6	‒0.938	0.522	3.530E-05	1.258E-03
GNB4	‒0.937	0.522	1.619E-03	2.231E-02
HNRNPF	‒0.936	0.523	2.560E-08	2.770E-06
PWWP2B	‒0.936	0.523	3.854E-03	4.133E-02
BTK	‒0.935	0.523	8.560E-05	2.467E-03
CD1C	‒0.934	0.523	6.590E-05	2.021E-03
NCOA4	‒0.933	0.524	8.071E-04	1.328E-02
POLH	‒0.932	0.524	2.100E-04	5.021E-03
SKAP2	‒0.932	0.524	1.219E-03	1.814E-02
ENSG00000246596	‒0.932	0.524	1.175E-03	1.768E-02
RXRA	‒0.931	0.524	2.741E-03	3.266E-02
ZDHHC7	‒0.929	0.525	3.696E-04	7.515E-03
LAX1	‒0.929	0.525	2.646E-04	5.947E-03
SDHAF1	‒0.927	0.526	1.095E-03	1.674E-02
IFITM2	‒0.927	0.526	3.236E-03	3.655E-02
SLC40A1	‒0.926	0.526	2.075E-03	2.663E-02
AK6	‒0.925	0.527	9.934E-04	1.556E-02
PREPL	‒0.925	0.527	1.990E-05	8.112E-04
MON1A	‒0.924	0.527	1.628E-03	2.235E-02
NA	‒0.924	0.527	1.399E-03	2.011E-02
UEVLD	‒0.924	0.527	1.906E-04	4.658E-03
IGHV1-2	‒0.924	0.527	2.565E-03	3.122E-02
SPTLC2	‒0.923	0.528	1.290E-06	8.310E-05
NCF1B	‒0.921	0.528	2.070E-06	1.247E-04
GGPS1	‒0.920	0.528	1.520E-06	9.540E-05
RBBP8	‒0.920	0.529	4.720E-05	1.581E-03
ADRB2	‒0.917	0.530	4.135E-03	4.321E-02
RHEX	‒0.917	0.530	2.283E-03	2.857E-02
FAM98B	‒0.913	0.531	6.730E-04	1.163E-02
SIT1	‒0.910	0.532	1.312E-03	1.916E-02
CHST15	‒0.910	0.532	1.979E-04	4.799E-03
C17orf80	‒0.910	0.532	1.520E-07	1.390E-05
FGR	‒0.909	0.533	3.613E-04	7.403E-03
C5orf51	‒0.906	0.534	1.616E-03	2.229E-02
KCTD15	‒0.905	0.534	2.207E-03	2.792E-02
ANXA4	‒0.905	0.534	6.878E-04	1.179E-02
HK3	‒0.904	0.534	1.316E-04	3.469E-03
CPNE5	‒0.904	0.534	4.818E-03	4.776E-02
RWDD2B	‒0.903	0.535	2.958E-03	3.441E-02
APOBEC3F	‒0.902	0.535	1.834E-03	2.433E-02
KMO	‒0.902	0.535	2.494E-04	5.708E-03
GOLIM4	‒0.901	0.536	2.739E-03	3.266E-02
CAMK1	‒0.899	0.536	5.070E-03	4.963E-02
TOR1B	‒0.898	0.537	1.202E-04	3.246E-03
MFAP1	‒0.898	0.537	1.393E-03	2.004E-02
TMEM106A	‒0.897	0.537	4.068E-04	8.080E-03
PLAGL2	‒0.897	0.537	3.079E-04	6.603E-03
RHOC	‒0.895	0.538	4.344E-04	8.465E-03
PFKFB4	‒0.894	0.538	2.956E-03	3.441E-02
KCTD11	‒0.891	0.539	6.009E-04	1.072E-02
ARCN1	‒0.891	0.539	2.290E-07	2.010E-05
HAVCR2	‒0.891	0.539	4.945E-03	4.875E-02
PIK3AP1	‒0.890	0.539	1.296E-04	3.434E-03
EIF2S1	‒0.890	0.540	3.690E-07	2.940E-05
NRM	‒0.889	0.540	4.771E-03	4.743E-02
B3GALT4	‒0.887	0.541	7.940E-05	2.334E-03
DAPK1	‒0.887	0.541	6.595E-04	1.146E-02
MYD88	‒0.887	0.541	1.820E-05	7.493E-04
CARD16	‒0.886	0.541	4.070E-05	1.408E-03
COA3	‒0.885	0.542	1.571E-03	2.189E-02
MYCBP	‒0.885	0.542	4.448E-04	8.582E-03
PABIR1	‒0.884	0.542	2.450E-05	9.530E-04
TMEM63C	‒0.884	0.542	3.373E-03	3.762E-02
TMEM250	‒0.883	0.542	3.470E-05	1.243E-03
IGLV1-51	‒0.882	0.543	1.730E-03	2.330E-02
TRIM21	‒0.882	0.543	1.918E-03	2.514E-02
DCLRE1A	‒0.880	0.543	2.415E-03	2.979E-02
PLD4	‒0.879	0.544	5.697E-04	1.033E-02
GPN3	‒0.878	0.544	5.717E-04	1.033E-02
HCCS	‒0.877	0.544	8.760E-05	2.517E-03
FIGNL1	‒0.877	0.545	6.853E-04	1.179E-02
ANKEF1	‒0.875	0.545	4.877E-03	4.824E-02
ATP23	‒0.874	0.546	1.076E-03	1.653E-02
ZFP3	‒0.873	0.546	1.860E-04	4.570E-03
AHCYL1	‒0.872	0.546	7.540E-05	2.228E-03
CYB561A3	‒0.870	0.547	3.738E-04	7.576E-03
NDUFAF1	‒0.868	0.548	7.415E-04	1.248E-02
SP110	‒0.865	0.549	1.025E-03	1.591E-02
DCAF12	‒0.865	0.549	3.621E-04	7.403E-03
TOR1A	‒0.864	0.549	5.340E-05	1.737E-03
ZNF816	‒0.862	0.550	2.478E-03	3.036E-02
CTSC	‒0.861	0.550	4.344E-04	8.465E-03
IL7R	‒0.861	0.551	1.050E-05	4.793E-04
SCRN1	‒0.860	0.551	8.573E-04	1.387E-02
SNX11	‒0.860	0.551	3.940E-05	1.374E-03
DUSP7	‒0.859	0.551	7.930E-04	1.311E-02
TMEM186	‒0.858	0.552	1.522E-03	2.131E-02
PARP14	‒0.857	0.552	4.924E-03	4.857E-02
GVINP1	‒0.857	0.552	6.777E-04	1.169E-02
TXNDC9	‒0.854	0.553	3.630E-03	3.971E-02
ALAS1	‒0.853	0.554	1.150E-06	7.630E-05
CDK14	‒0.850	0.555	2.801E-03	3.307E-02
ZNF737	‒0.849	0.555	1.275E-04	3.399E-03
VASP	‒0.848	0.555	6.150E-05	1.925E-03
TLR6	‒0.847	0.556	4.776E-03	4.745E-02
IDH1	‒0.847	0.556	5.150E-04	9.514E-03
CAMKK2	‒0.846	0.556	3.040E-05	1.121E-03
GTF2E1	‒0.846	0.556	3.155E-04	6.722E-03
STT3A	‒0.846	0.556	7.780E-07	5.470E-05
FBXO45	‒0.845	0.557	4.381E-03	4.500E-02
DPYSL2	‒0.845	0.557	9.826E-04	1.544E-02
SLC25A19	‒0.844	0.557	2.963E-04	6.401E-03
MTF1	‒0.844	0.557	3.516E-04	7.278E-03
DYNC1I2	‒0.843	0.557	3.290E-06	1.857E-04
MGME1	‒0.841	0.558	3.640E-06	2.023E-04
CD300A	‒0.840	0.559	4.670E-03	4.684E-02
LIG4	‒0.838	0.559	1.942E-03	2.536E-02
SNAPC3	‒0.837	0.560	1.417E-04	3.661E-03
NMT1	‒0.836	0.560	2.370E-05	9.334E-04
ZW10	‒0.836	0.560	8.179E-04	1.341E-02
CD244	‒0.835	0.560	2.957E-04	6.397E-03
ZC3H10	‒0.835	0.561	2.703E-03	3.238E-02
ZNF226	‒0.835	0.561	7.200E-06	3.487E-04
CHSY1	‒0.834	0.561	2.531E-04	5.770E-03
GBP4	‒0.833	0.561	2.213E-03	2.796E-02
CHCHD4	‒0.832	0.562	2.198E-03	2.784E-02
SH2B2	‒0.832	0.562	4.452E-03	4.550E-02
WDR5B	‒0.828	0.563	3.222E-04	6.787E-03
SLC25A24	‒0.828	0.563	2.719E-03	3.246E-02
TRIM68	‒0.828	0.563	1.426E-03	2.036E-02
PLPBP	‒0.825	0.565	4.750E-05	1.584E-03
GVQW3	‒0.824	0.565	1.770E-03	2.368E-02
HSPA8	‒0.822	0.565	2.737E-04	6.060E-03
CRYZ	‒0.818	0.567	2.996E-04	6.450E-03
DNAJA1	‒0.818	0.567	3.882E-04	7.840E-03
FASTKD3	‒0.816	0.568	8.419E-04	1.367E-02
ZKSCAN3	‒0.814	0.569	2.612E-03	3.163E-02
MTPN	‒0.812	0.570	2.568E-04	5.831E-03
CHMP5	‒0.812	0.570	3.901E-03	4.162E-02
CTR9	‒0.811	0.570	6.160E-05	1.925E-03
SNAPIN	‒0.811	0.570	1.112E-04	3.059E-03
SLAMF6	‒0.807	0.572	8.430E-05	2.444E-03
NMNAT1	‒0.806	0.572	4.680E-03	4.691E-02
WASF1	‒0.805	0.572	3.541E-03	3.898E-02
ARMT1	‒0.804	0.573	4.017E-03	4.246E-02
CHURC1	‒0.800	0.574	3.104E-03	3.559E-02
RBM6	‒0.800	0.574	3.925E-04	7.917E-03
DCAF7	‒0.799	0.575	5.390E-07	3.970E-05
PRDX3	‒0.799	0.575	2.480E-04	5.700E-03
HSCB	‒0.798	0.575	3.370E-03	3.761E-02
RGS19	‒0.795	0.576	1.740E-05	7.220E-04
JPT2	‒0.795	0.577	6.867E-04	1.179E-02
PLXNB2	‒0.794	0.577	1.454E-03	2.061E-02
SLC35A5	‒0.793	0.577	2.649E-04	5.947E-03
CD72	‒0.791	0.578	4.041E-04	8.043E-03
SPRYD3	‒0.791	0.578	8.190E-05	2.384E-03
DARS2	‒0.790	0.578	3.140E-04	6.699E-03
ZNF200	‒0.788	0.579	2.690E-05	1.015E-03
TMOD3	‒0.787	0.579	1.359E-03	1.970E-02
ARL6IP5	‒0.787	0.580	1.060E-07	1.040E-05
DCTPP1	‒0.786	0.580	2.789E-03	3.295E-02
CALML4	‒0.786	0.580	4.506E-03	4.589E-02
YWHAG	‒0.786	0.580	2.025E-03	2.618E-02
TMBIM1	‒0.782	0.581	2.160E-06	1.273E-04
VKORC1L1	‒0.779	0.583	1.136E-04	3.103E-03
CNP	‒0.778	0.583	3.691E-03	4.011E-02
CAP1	‒0.776	0.584	2.844E-04	6.234E-03
ZNF234	‒0.775	0.584	1.437E-04	3.706E-03
BASP1	‒0.773	0.585	5.920E-05	1.881E-03
TBC1D24	‒0.771	0.586	1.164E-03	1.754E-02
AHSA1	‒0.771	0.586	4.257E-04	8.354E-03
LAMP2	‒0.769	0.587	1.589E-04	4.006E-03
SYK	‒0.769	0.587	2.178E-03	2.772E-02
MRPS18B	‒0.769	0.587	1.626E-03	2.235E-02
TARS1	‒0.769	0.587	4.520E-05	1.530E-03
MCMBP	‒0.768	0.587	6.120E-05	1.921E-03
OSTM1	‒0.768	0.587	4.755E-04	8.963E-03
CD53	‒0.767	0.588	1.440E-05	6.211E-04
EVI2A	‒0.767	0.588	4.626E-04	8.797E-03
B4GALT5	‒0.765	0.588	1.846E-03	2.443E-02
TXLNA	‒0.763	0.589	1.096E-04	3.023E-03
JAK2	‒0.763	0.589	6.770E-05	2.063E-03
TPP1	‒0.763	0.589	3.500E-03	3.861E-02
BPNT1	‒0.762	0.590	3.441E-03	3.811E-02
NDUFA8	‒0.762	0.590	2.972E-03	3.453E-02
HLA-DOA	‒0.762	0.590	1.671E-04	4.181E-03
TAPBPL	‒0.762	0.590	2.182E-03	2.772E-02
GIMAP6	‒0.759	0.591	3.270E-05	1.186E-03
SLC35C1	‒0.759	0.591	2.860E-03	3.360E-02
UBXN2B	‒0.756	0.592	2.081E-04	5.003E-03
ZNF397	‒0.756	0.592	3.820E-07	2.990E-05
CEP89	‒0.756	0.592	3.606E-03	3.958E-02
PARP4	‒0.755	0.593	2.203E-04	5.215E-03
GSTO1	‒0.754	0.593	8.860E-05	2.537E-03
IRF2	‒0.754	0.593	5.590E-06	2.835E-04
VPS41	‒0.753	0.593	1.407E-03	2.013E-02
ZNF213	‒0.753	0.593	3.088E-03	3.548E-02
TRIM27	‒0.753	0.594	1.370E-06	8.750E-05
YIPF4	‒0.752	0.594	5.883E-04	1.055E-02
ACADM	‒0.752	0.594	2.713E-03	3.243E-02
CAPZA2	‒0.751	0.594	6.572E-04	1.144E-02
ZKSCAN4	‒0.749	0.595	1.423E-03	2.033E-02
ZNF780B	‒0.749	0.595	7.360E-05	2.194E-03
TNFAIP1	‒0.748	0.595	2.610E-04	5.901E-03
NFE2L1	‒0.748	0.596	8.379E-04	1.362E-02
HLA-DPA1	‒0.747	0.596	5.163E-04	9.526E-03
GYG1	‒0.746	0.596	1.608E-03	2.225E-02
PURA	‒0.744	0.597	2.421E-04	5.602E-03
LFNG	‒0.743	0.598	1.014E-03	1.581E-02
C11orf68	‒0.742	0.598	7.261E-04	1.231E-02
C2orf42	‒0.740	0.599	2.705E-03	3.238E-02
GID4	‒0.739	0.599	1.028E-04	2.875E-03
CCDC117	‒0.738	0.600	1.763E-03	2.363E-02
TSPAN31	‒0.737	0.600	1.678E-03	2.281E-02
PRUNE1	‒0.735	0.601	4.325E-04	8.457E-03
SWAP70	‒0.734	0.601	2.087E-04	5.011E-03
THRAP3	‒0.731	0.602	8.680E-08	8.770E-06
MOB3C	‒0.730	0.603	3.011E-03	3.486E-02
RAB8A	‒0.730	0.603	1.505E-04	3.858E-03
RNGTT	‒0.729	0.603	4.728E-03	4.716E-02
MRPL15	‒0.726	0.605	4.012E-04	8.025E-03
DHRS7	‒0.724	0.606	3.188E-04	6.754E-03
C1GALT1C1	‒0.723	0.606	3.879E-03	4.146E-02
ZNF146	‒0.723	0.606	2.930E-07	2.440E-05
SDF2	‒0.723	0.606	2.940E-04	6.372E-03
IFNGR2	‒0.722	0.606	4.709E-04	8.916E-03
E2F5	‒0.722	0.606	9.697E-04	1.533E-02
HVCN1	‒0.721	0.606	3.394E-04	7.077E-03
JKAMP	‒0.721	0.607	1.312E-03	1.916E-02
PRKCD	‒0.720	0.607	7.843E-04	1.303E-02
RP2	‒0.719	0.608	3.960E-03	4.203E-02
STIP1	‒0.717	0.608	6.692E-04	1.159E-02
ORMDL2	‒0.717	0.608	1.371E-03	1.977E-02
SHMT1	‒0.716	0.609	1.467E-03	2.074E-02
SLC25A20	‒0.716	0.609	5.278E-04	9.664E-03
HSBP1	‒0.715	0.609	1.230E-04	3.306E-03
CTSH	‒0.714	0.610	1.713E-03	2.314E-02
ARPC5	‒0.713	0.610	1.708E-04	4.253E-03
LARS2	‒0.712	0.611	2.247E-04	5.287E-03
DOK3	‒0.711	0.611	2.647E-03	3.192E-02
BRD8	‒0.710	0.611	4.840E-07	3.630E-05
NEK4	‒0.708	0.612	2.598E-03	3.152E-02
RBM4B	‒0.707	0.612	6.340E-04	1.114E-02
ZBTB7B	‒0.707	0.612	7.401E-04	1.247E-02
SLC35B3	‒0.704	0.614	3.171E-04	6.739E-03
HAT1	‒0.704	0.614	3.129E-03	3.569E-02
GIMAP2	‒0.704	0.614	2.470E-07	2.110E-05
ATP6V0E1	‒0.704	0.614	1.282E-04	3.411E-03
SNX27	‒0.703	0.614	1.127E-03	1.711E-02
PDCD7	‒0.703	0.614	2.816E-04	6.198E-03
ECHDC1	‒0.702	0.615	4.178E-04	8.248E-03
ZNF486	‒0.701	0.615	4.571E-03	4.624E-02
MTREX	‒0.699	0.616	2.644E-04	5.947E-03
SNX19	‒0.697	0.617	2.376E-03	2.940E-02
OMA1	‒0.697	0.617	7.030E-05	2.113E-03
DLAT	‒0.696	0.617	4.279E-03	4.420E-02
RPP25L	‒0.695	0.618	2.389E-03	2.950E-02
VEZT	‒0.693	0.619	5.126E-04	9.478E-03
OGFOD1	‒0.690	0.620	1.115E-03	1.699E-02
TTI2	‒0.690	0.620	4.590E-03	4.630E-02
TBL2	‒0.689	0.620	1.505E-04	3.858E-03
ZNF608	‒0.688	0.621	2.782E-03	3.295E-02
UBE4A	‒0.687	0.621	3.200E-05	1.165E-03
MCEE	‒0.687	0.621	2.028E-03	2.620E-02
DAXX	‒0.686	0.622	3.421E-04	7.124E-03
IFNAR1	‒0.685	0.622	2.224E-04	5.241E-03
PAXIP1-AS2	‒0.683	0.623	1.178E-03	1.771E-02
TOLLIP	‒0.683	0.623	2.260E-05	9.004E-04
BUD13	‒0.683	0.623	4.870E-05	1.615E-03
TIA1	‒0.682	0.623	5.530E-06	2.817E-04
PCMT1	‒0.682	0.623	7.390E-05	2.200E-03
ATP6V0D1	‒0.682	0.624	1.010E-04	2.837E-03
HSD17B4	‒0.680	0.624	1.400E-05	6.077E-04
LAIR1	‒0.680	0.624	4.060E-05	1.405E-03
ITGAX	‒0.679	0.624	9.025E-04	1.444E-02
RITA1	‒0.679	0.625	6.116E-04	1.087E-02
SFT2D1	‒0.679	0.625	9.540E-05	2.694E-03
UTP6	‒0.679	0.625	1.660E-05	6.972E-04
CNIH4	‒0.678	0.625	1.843E-04	4.542E-03
LYN	‒0.677	0.625	4.031E-04	8.043E-03
CTNND1	‒0.675	0.626	1.995E-03	2.590E-02
PSMB8-AS1	‒0.675	0.626	1.674E-03	2.279E-02
REEP5	‒0.675	0.627	1.024E-03	1.591E-02
TLR1	‒0.674	0.627	3.392E-03	3.776E-02
MGAT2	‒0.673	0.627	8.250E-04	1.346E-02
CRKL	‒0.670	0.628	3.963E-04	7.965E-03
SLC49A3	‒0.670	0.629	3.924E-03	4.173E-02
SEC23IP	‒0.668	0.629	1.127E-04	3.089E-03
NADK	‒0.668	0.630	1.154E-03	1.744E-02
HSPH1	‒0.666	0.630	7.160E-05	2.136E-03
GPR65	‒0.665	0.631	1.014E-03	1.581E-02
APEX2	‒0.665	0.631	4.314E-03	4.450E-02
CPLANE1	‒0.664	0.631	3.376E-03	3.762E-02
MAP1S	‒0.663	0.631	1.552E-03	2.168E-02
SNAPC5	‒0.663	0.632	1.127E-03	1.711E-02
HLA-DPB1	‒0.663	0.632	3.619E-04	7.403E-03
APOBEC3D	‒0.661	0.633	6.868E-04	1.179E-02
LYSMD2	‒0.660	0.633	1.488E-03	2.095E-02
FCMR	‒0.660	0.633	5.730E-07	4.150E-05
ZBTB38	‒0.659	0.633	1.204E-04	3.246E-03
GDI2	‒0.659	0.633	6.120E-04	1.087E-02
VTA1	‒0.657	0.634	7.080E-04	1.207E-02
MR1	‒0.654	0.635	7.883E-04	1.306E-02
ICAM2	‒0.654	0.635	3.458E-03	3.826E-02
PHF11	‒0.653	0.636	1.591E-03	2.213E-02
ACTR2	‒0.652	0.636	5.789E-04	1.044E-02
CRLS1	‒0.650	0.637	4.520E-04	8.652E-03
GM2A	‒0.649	0.638	9.820E-04	1.544E-02
SLC2A6	‒0.648	0.638	1.200E-06	7.840E-05
MARCHF7	‒0.648	0.638	2.294E-03	2.863E-02
ALOX5AP	‒0.646	0.639	3.902E-03	4.162E-02
COX15	‒0.646	0.639	1.360E-03	1.970E-02
DOCK8	‒0.646	0.639	1.360E-05	5.939E-04
TRIM4	‒0.644	0.640	7.500E-06	3.611E-04
ILK	‒0.644	0.640	1.388E-04	3.601E-03
LCP1	‒0.643	0.640	4.403E-03	4.518E-02
CLTA	‒0.642	0.641	1.195E-04	3.237E-03
IL10RB	‒0.640	0.642	3.723E-03	4.034E-02
TMBIM6	‒0.639	0.642	2.440E-05	9.504E-04
TIMMDC1	‒0.639	0.642	1.759E-04	4.362E-03
HEIH	‒0.638	0.643	3.753E-04	7.598E-03
DENND5B	‒0.638	0.643	6.972E-04	1.191E-02
PPP1R11	‒0.638	0.643	2.980E-05	1.109E-03
UBE2V2	‒0.637	0.643	2.890E-04	6.312E-03
C11orf24	‒0.637	0.643	2.280E-03	2.857E-02
NME6	‒0.636	0.643	3.406E-03	3.785E-02
BIRC2	‒0.636	0.643	6.780E-05	2.063E-03
NIT1	‒0.636	0.644	1.581E-04	3.997E-03
ACTR1A	‒0.635	0.644	1.921E-03	2.517E-02
ZNF260	‒0.635	0.644	2.911E-03	3.415E-02
C21orf91	‒0.634	0.644	2.697E-04	5.994E-03
COPB2	‒0.633	0.645	1.357E-04	3.539E-03
VCL	‒0.632	0.645	4.889E-03	4.832E-02
FRAT1	‒0.630	0.646	4.730E-04	8.934E-03
S100A4	‒0.630	0.646	3.008E-03	3.486E-02
AIMP2	‒0.629	0.647	2.159E-03	2.752E-02
CREBL2	‒0.629	0.647	2.557E-03	3.114E-02
RNF5	‒0.627	0.647	7.217E-04	1.225E-02
AHNAK	‒0.627	0.647	4.531E-03	4.603E-02
AFTPH	‒0.626	0.648	2.755E-03	3.276E-02
KRCC1	‒0.625	0.648	1.733E-04	4.303E-03
BLNK	‒0.625	0.648	4.971E-03	4.889E-02
ETFA	‒0.623	0.649	1.257E-04	3.368E-03
SP1	‒0.622	0.650	1.998E-03	2.592E-02
AMMECR1L	‒0.621	0.650	2.412E-04	5.588E-03
MITD1	‒0.621	0.650	1.673E-03	2.279E-02
MRS2	‒0.620	0.651	1.214E-03	1.810E-02
GRK3	‒0.619	0.651	4.105E-03	4.302E-02
SNX20	‒0.618	0.651	5.810E-05	1.862E-03
PTPN6	‒0.618	0.652	3.015E-04	6.473E-03
ZKSCAN1	‒0.618	0.652	3.728E-04	7.566E-03
NIPA2	‒0.617	0.652	2.034E-03	2.625E-02
POC5	‒0.616	0.652	8.359E-04	1.360E-02
CCT5	‒0.616	0.653	1.356E-04	3.539E-03
ANXA1	‒0.616	0.653	4.253E-03	4.404E-02
FGFR1OP2	‒0.614	0.653	3.125E-04	6.675E-03
MED12	‒0.614	0.653	3.740E-05	1.314E-03
ZNF672	‒0.613	0.654	1.240E-03	1.833E-02
RPE	‒0.611	0.655	6.204E-04	1.096E-02
COPS4	‒0.610	0.655	7.730E-04	1.289E-02
FXR2	‒0.610	0.655	2.358E-04	5.502E-03
COP1	‒0.610	0.655	1.446E-03	2.053E-02
DENND2D	‒0.609	0.656	1.316E-03	1.921E-02
FUCA1	‒0.609	0.656	9.178E-04	1.465E-02
KRBOX4	‒0.607	0.656	1.301E-03	1.907E-02
MAT2B	‒0.603	0.658	1.130E-05	5.131E-04
NUP62	‒0.603	0.659	7.100E-05	2.122E-03
NA	‒0.602	0.659	3.171E-03	3.599E-02
MTHFD2	‒0.602	0.659	4.403E-04	8.540E-03
ZNF721	‒0.601	0.659	5.302E-04	9.696E-03
HERC2P2	‒0.601	0.659	3.240E-06	1.834E-04
MSANTD4	‒0.600	0.660	4.577E-03	4.625E-02
DNAJC7	‒0.600	0.660	3.606E-04	7.403E-03
ELF4	‒0.600	0.660	2.594E-03	3.152E-02
GLT8D1	‒0.599	0.660	6.020E-05	1.898E-03
SPIDR	‒0.599	0.660	3.138E-03	3.577E-02
NLRX1	‒0.599	0.660	4.019E-03	4.246E-02
ACTR3	‒0.598	0.661	1.266E-04	3.380E-03
ZNF175	‒0.597	0.661	4.158E-03	4.334E-02
NDUFB3	‒0.596	0.662	7.103E-04	1.209E-02
SH2D3C	‒0.596	0.662	3.037E-03	3.504E-02
ZNF197	‒0.596	0.662	4.523E-04	8.652E-03
UMPS	‒0.595	0.662	2.930E-03	3.423E-02
SEPHS2	‒0.594	0.662	3.244E-03	3.660E-02
ZNF586	‒0.594	0.662	1.264E-04	3.380E-03
LEO1	‒0.594	0.663	3.921E-03	4.173E-02
RAC2	‒0.593	0.663	3.166E-03	3.596E-02
SLC16A3	‒0.593	0.663	2.532E-04	5.770E-03
PPP1R8	‒0.592	0.663	3.268E-03	3.675E-02
RBL2	‒0.592	0.664	3.084E-04	6.605E-03
ABRACL	‒0.591	0.664	2.282E-03	2.857E-02
ARFIP1	‒0.590	0.664	4.555E-03	4.613E-02
KLHL9	‒0.590	0.664	8.259E-04	1.346E-02
TBC1D1	‒0.589	0.665	1.603E-03	2.224E-02
AGTRAP	‒0.588	0.665	1.624E-03	2.235E-02
MICB	‒0.588	0.665	4.557E-03	4.613E-02
ASH2L	‒0.587	0.666	3.380E-05	1.215E-03
RTCB	‒0.586	0.666	1.095E-03	1.674E-02
MRM3	0.587	1.503	2.585E-03	3.143E-02
HNRNPA1	0.588	1.503	2.640E-05	1.007E-03
REXO1	0.589	1.504	4.524E-03	4.599E-02
TRA2B	0.589	1.505	2.448E-04	5.648E-03
EME2	0.590	1.505	3.269E-03	3.675E-02
DDX51	0.592	1.507	9.971E-04	1.558E-02
CHD1	0.592	1.508	1.627E-03	2.235E-02
MED9	0.592	1.508	4.072E-03	4.282E-02
ASNS	0.593	1.508	2.197E-04	5.207E-03
ZNF831	0.596	1.511	3.658E-04	7.469E-03
CLDND1	0.599	1.514	3.464E-04	7.188E-03
FBXO32	0.601	1.516	2.468E-03	3.026E-02
SREBF1	0.603	1.519	2.245E-03	2.829E-02
GMFB	0.604	1.519	1.149E-03	1.739E-02
ATN1	0.605	1.521	1.502E-03	2.112E-02
DNAJB14	0.606	1.522	2.373E-03	2.940E-02
KANSL2	0.606	1.522	4.408E-04	8.540E-03
MLLT3	0.609	1.525	1.763E-04	4.365E-03
KLF11	0.609	1.525	4.225E-03	4.389E-02
FOXP1	0.610	1.526	5.710E-05	1.833E-03
MZT2B	0.611	1.527	4.147E-03	4.330E-02
NOP53	0.612	1.528	3.736E-03	4.040E-02
POU2F1	0.612	1.528	1.041E-04	2.900E-03
RFX3	0.612	1.529	7.849E-04	1.303E-02
KRR1	0.617	1.534	4.080E-06	2.210E-04
SSBP1	0.617	1.534	6.140E-06	3.038E-04
MCRIP2	0.619	1.536	1.407E-03	2.013E-02
LRP5L	0.620	1.537	3.344E-03	3.739E-02
LEMD3	0.621	1.537	4.250E-06	2.286E-04
MEF2D	0.621	1.538	9.140E-07	6.240E-05
ENSG00000261490	0.621	1.538	3.129E-03	3.569E-02
PAG1	0.622	1.539	7.514E-04	1.261E-02
CCDC57	0.623	1.540	4.723E-04	8.933E-03
PRDM2	0.623	1.540	2.620E-05	1.002E-03
SFXN1	0.624	1.541	1.611E-03	2.228E-02
FRS2	0.625	1.542	2.355E-03	2.924E-02
SGMS1	0.625	1.542	2.255E-03	2.833E-02
H1-10	0.626	1.544	3.870E-06	2.106E-04
INTS1	0.626	1.544	1.236E-03	1.831E-02
TRERF1	0.631	1.549	1.605E-03	2.225E-02
PPP1R15A	0.632	1.549	1.870E-05	7.664E-04
RHNO1	0.635	1.553	3.557E-03	3.912E-02
ANAPC15	0.635	1.553	1.214E-04	3.267E-03
SLC5A6	0.636	1.554	4.500E-06	2.390E-04
COX4I1	0.637	1.555	8.740E-08	8.770E-06
MRPL44	0.637	1.555	3.803E-04	7.690E-03
FBXL3	0.638	1.556	4.040E-05	1.404E-03
LCOR	0.639	1.557	5.050E-06	2.628E-04
TGIF1	0.641	1.560	3.228E-03	3.649E-02
FKBP11	0.642	1.560	1.951E-03	2.542E-02
KDM7A	0.642	1.560	2.144E-04	5.111E-03
SNHG16	0.643	1.562	2.941E-04	6.372E-03
SNRK	0.644	1.562	5.018E-04	9.348E-03
SLC25A36	0.644	1.563	4.191E-04	8.263E-03
SUPV3L1	0.644	1.563	5.874E-04	1.055E-02
PNMA1	0.644	1.563	4.122E-03	4.314E-02
PAFAH1B2	0.644	1.563	1.967E-04	4.783E-03
ATG2A	0.645	1.564	7.069E-04	1.207E-02
ELF2	0.646	1.565	2.100E-06	1.258E-04
FAM229A	0.646	1.565	4.983E-03	4.899E-02
ATF4	0.647	1.566	3.570E-05	1.269E-03
FAM102A	0.648	1.567	2.165E-03	2.757E-02
EAF1	0.649	1.569	1.721E-03	2.321E-02
TMEM120B	0.650	1.570	3.680E-03	4.004E-02
ARID2	0.652	1.572	3.384E-04	7.066E-03
TRIM52-AS1	0.654	1.573	2.059E-03	2.645E-02
RPS14	0.654	1.574	2.686E-04	5.994E-03
NFKBIE	0.655	1.574	5.094E-03	4.981E-02
MEGF6	0.655	1.574	1.771E-03	2.368E-02
SNRPA1	0.658	1.578	1.883E-04	4.620E-03
LMTK2	0.658	1.578	2.750E-05	1.027E-03
NSMCE3	0.659	1.579	2.660E-05	1.008E-03
EPB41L4A-AS1	0.660	1.580	2.530E-05	9.788E-04
POU6F1	0.661	1.581	1.190E-03	1.785E-02
ENSG00000257354	0.662	1.582	3.623E-03	3.971E-02
ZNF408	0.662	1.583	2.807E-03	3.310E-02
CHD7	0.662	1.583	1.470E-04	3.785E-03
ZNF432	0.663	1.583	1.236E-03	1.831E-02
B3GNTL1	0.663	1.583	7.535E-04	1.263E-02
NFKBIZ	0.665	1.585	3.061E-03	3.526E-02
NFKBIB	0.666	1.587	6.410E-04	1.123E-02
BICDL1	0.666	1.587	4.001E-04	8.013E-03
PIK3R1	0.668	1.589	1.102E-03	1.682E-02
KAT6B	0.668	1.589	2.580E-05	9.886E-04
IGF1R	0.669	1.589	1.102E-03	1.682E-02
BTG2	0.669	1.590	2.494E-04	5.708E-03
LEPROTL1	0.670	1.591	1.651E-03	2.256E-02
NECAP1	0.670	1.591	1.750E-06	1.078E-04
AP1G2-AS1	0.672	1.593	4.120E-05	1.420E-03
SVIP	0.674	1.595	9.720E-04	1.535E-02
ISCA1	0.675	1.596	1.640E-03	2.245E-02
PNRC1	0.675	1.597	7.980E-06	3.809E-04
PIDD1	0.678	1.600	8.583E-04	1.387E-02
VAMP2	0.679	1.601	1.820E-08	2.020E-06
ADAMTS10	0.680	1.602	4.540E-03	4.607E-02
TCF7	0.682	1.604	1.200E-05	5.353E-04
EXD3	0.683	1.605	1.214E-03	1.810E-02
TIAM1	0.683	1.605	3.855E-03	4.133E-02
LINC-PINT	0.686	1.609	2.743E-03	3.266E-02
ATXN1L	0.689	1.613	8.960E-05	2.560E-03
MBIP	0.691	1.615	2.098E-03	2.688E-02
NOL4L	0.692	1.615	4.613E-04	8.783E-03
RPL36A	0.692	1.616	3.688E-03	4.011E-02
VCPKMT	0.693	1.616	6.204E-04	1.096E-02
CBX6	0.693	1.617	2.331E-04	5.460E-03
MRPL41	0.695	1.618	3.350E-03	3.744E-02
ENSG00000280135	0.695	1.618	2.653E-03	3.196E-02
ADNP2	0.695	1.619	1.100E-05	4.997E-04
ZNF800	0.701	1.626	3.030E-05	1.118E-03
EPHB4	0.704	1.629	2.807E-03	3.310E-02
EIF5	0.705	1.630	1.960E-06	1.186E-04
PGM2L1	0.705	1.630	3.167E-04	6.738E-03
DYNLT1	0.706	1.631	3.925E-03	4.173E-02
CNNM2	0.706	1.631	6.423E-04	1.124E-02
PPIL4	0.707	1.632	2.334E-03	2.902E-02
TRMO	0.707	1.633	1.910E-03	2.506E-02
HIVEP1	0.707	1.633	1.326E-04	3.484E-03
BEX4	0.708	1.633	2.802E-04	6.175E-03
MRPL9	0.708	1.634	1.324E-04	3.484E-03
TLE2	0.710	1.636	3.869E-03	4.140E-02
EIF1B	0.712	1.638	8.150E-05	2.379E-03
SNHG1	0.712	1.638	1.213E-03	1.810E-02
ZNF814	0.714	1.640	2.058E-04	4.963E-03
ENSG00000230551	0.715	1.642	4.259E-03	4.407E-02
GOLT1B	0.716	1.642	1.880E-03	2.473E-02
MFGE8	0.717	1.644	1.714E-03	2.314E-02
RICTOR	0.718	1.645	1.176E-04	3.195E-03
UTP15	0.719	1.646	1.959E-04	4.771E-03
SIVA1	0.720	1.647	1.090E-04	3.014E-03
SORBS3	0.723	1.650	9.648E-04	1.527E-02
EMD	0.724	1.652	2.687E-04	5.994E-03
TRABD	0.727	1.655	6.177E-04	1.094E-02
EIF2AK3	0.727	1.655	2.914E-04	6.346E-03
ZNF134	0.727	1.655	4.069E-04	8.080E-03
PIK3IP1	0.728	1.656	2.966E-04	6.401E-03
NCBP2AS2	0.728	1.656	5.660E-05	1.821E-03
DDX39A	0.729	1.658	1.020E-04	2.857E-03
PHF1	0.731	1.660	2.120E-05	8.495E-04
ARRDC2	0.732	1.661	1.644E-03	2.249E-02
GABARAPL1	0.733	1.663	1.934E-03	2.528E-02
SRSF10	0.737	1.666	5.980E-06	2.990E-04
CHRM3-AS2	0.737	1.666	1.470E-03	2.076E-02
ZNF815P	0.739	1.669	4.427E-04	8.558E-03
MGST3	0.742	1.672	1.709E-03	2.314E-02
AP3M2	0.742	1.672	4.388E-03	4.505E-02
JOSD1	0.743	1.673	1.210E-06	7.880E-05
ODC1	0.743	1.674	2.740E-05	1.027E-03
KPNA5	0.749	1.680	4.024E-04	8.039E-03
GOLGA7B	0.753	1.685	1.060E-03	1.632E-02
MAP3K1	0.755	1.688	3.930E-04	7.918E-03
AK5	0.756	1.689	2.327E-04	5.460E-03
C5orf24	0.756	1.689	6.860E-07	4.840E-05
KLHL24	0.758	1.691	2.270E-05	9.013E-04
TAF3	0.759	1.692	1.870E-05	7.668E-04
FAM160B1	0.760	1.693	1.333E-04	3.498E-03
BICRA	0.760	1.693	2.379E-04	5.529E-03
AKT1S1	0.761	1.695	8.010E-05	2.345E-03
RLIM	0.763	1.697	1.340E-06	8.610E-05
MAPKAPK5-AS1	0.768	1.703	2.970E-07	2.470E-05
NA	0.772	1.707	4.109E-03	4.303E-02
KLHL15	0.772	1.707	3.060E-05	1.127E-03
TIPARP	0.776	1.713	1.574E-04	3.991E-03
FAM177A1	0.776	1.713	2.670E-04	5.980E-03
CIDECP1	0.778	1.715	4.942E-04	9.244E-03
MTHFD2L	0.778	1.715	2.120E-03	2.710E-02
NPIPB5	0.780	1.717	3.472E-03	3.836E-02
LMO7	0.781	1.718	5.950E-06	2.985E-04
NOXA1	0.781	1.718	2.253E-03	2.832E-02
TMEM201	0.782	1.719	6.646E-04	1.153E-02
PPP1R32	0.782	1.720	2.050E-03	2.637E-02
IL1B	0.783	1.720	1.594E-03	2.215E-02
MYL5	0.783	1.720	2.121E-03	2.710E-02
ENSG00000264112	0.787	1.726	2.120E-06	1.266E-04
ENSG00000279838	0.789	1.728	4.723E-03	4.716E-02
ZNF805	0.789	1.728	2.189E-04	5.196E-03
CLK1	0.790	1.729	4.560E-10	8.260E-08
PTS	0.791	1.730	3.444E-03	3.813E-02
RNF125	0.791	1.731	3.585E-04	7.393E-03
PSTK	0.792	1.732	3.158E-03	3.592E-02
FOXK1	0.795	1.735	8.360E-06	3.911E-04
SOX12	0.796	1.736	1.260E-06	8.130E-05
ZNF446	0.796	1.736	2.381E-03	2.943E-02
MBNL2	0.796	1.737	1.530E-05	6.520E-04
SNHG20	0.796	1.737	6.018E-04	1.072E-02
RAMAC	0.798	1.738	2.212E-03	2.796E-02
NA	0.798	1.739	1.333E-03	1.940E-02
NFKBID	0.799	1.740	1.740E-05	7.220E-04
HBP1	0.800	1.741	7.925E-04	1.311E-02
CHIC1	0.805	1.747	8.110E-05	2.370E-03
ISCU	0.805	1.747	4.390E-06	2.343E-04
SGTB	0.807	1.750	7.781E-04	1.296E-02
TRAF4	0.809	1.752	3.730E-05	1.313E-03
ZFP36	0.811	1.754	1.494E-04	3.841E-03
EPHA4	0.812	1.755	1.045E-04	2.908E-03
CDK11A	0.812	1.756	1.310E-05	5.747E-04
ANO9	0.813	1.757	2.292E-03	2.863E-02
TYW5	0.815	1.760	4.949E-04	9.244E-03
RETREG1	0.819	1.764	5.862E-04	1.054E-02
CD28	0.822	1.768	6.437E-04	1.125E-02
KDM6B	0.826	1.772	4.410E-07	3.380E-05
ARL5B	0.826	1.773	3.616E-04	7.403E-03
SLC2A4RG	0.826	1.773	5.712E-04	1.033E-02
PET100	0.831	1.779	6.930E-05	2.095E-03
ZFYVE28	0.835	1.784	1.633E-03	2.237E-02
ZNF587B	0.837	1.787	2.430E-05	9.504E-04
PLCL1	0.847	1.799	1.886E-03	2.478E-02
SMURF1	0.850	1.802	5.870E-05	1.873E-03
DDX24	0.850	1.802	2.100E-07	1.860E-05
STARD10	0.853	1.806	2.916E-03	3.416E-02
POLR1C	0.853	1.806	8.020E-06	3.816E-04
TIGD1	0.855	1.809	2.612E-03	3.163E-02
EIF1	0.855	1.809	4.610E-06	2.439E-04
HMGB2	0.856	1.810	7.320E-06	3.536E-04
ENSG00000272529	0.856	1.810	5.188E-04	9.551E-03
H3-3B	0.856	1.811	7.380E-09	9.680E-07
ENSG00000235859	0.857	1.812	1.711E-03	2.314E-02
CEP85L	0.858	1.812	2.426E-03	2.982E-02
RBM7	0.859	1.814	5.269E-04	9.661E-03
LRRC8B	0.862	1.817	6.560E-06	3.205E-04
RASGEF1B	0.862	1.818	1.402E-04	3.632E-03
CNBD2	0.863	1.819	6.823E-04	1.176E-02
OTUD1	0.863	1.819	6.160E-06	3.038E-04
BCL10	0.863	1.819	1.669E-04	4.181E-03
P2RY11	0.864	1.820	1.428E-03	2.037E-02
ZNF844	0.869	1.826	7.990E-08	8.120E-06
NGDN	0.871	1.829	1.550E-08	1.760E-06
ENO2	0.874	1.833	2.610E-04	5.901E-03
ENSG00000271964	0.876	1.836	1.054E-03	1.625E-02
AMMECR1	0.877	1.837	3.840E-06	2.096E-04
YPEL5	0.880	1.840	3.000E-07	2.470E-05
MGAT4A	0.881	1.841	1.650E-05	6.972E-04
HSF2	0.881	1.842	7.080E-05	2.121E-03
ZXDB	0.882	1.843	6.000E-05	1.895E-03
ZEB1	0.884	1.846	2.760E-06	1.599E-04
HAGHL	0.886	1.847	2.784E-03	3.295E-02
AEN	0.886	1.847	4.430E-07	3.390E-05
MYLIP	0.886	1.849	3.070E-07	2.510E-05
IER2	0.888	1.851	9.330E-08	9.310E-06
ENSG00000261526	0.889	1.852	1.907E-04	4.658E-03
GZF1	0.890	1.853	9.380E-05	2.668E-03
PMAIP1	0.890	1.854	4.041E-03	4.256E-02
RHPN1	0.894	1.859	2.180E-03	2.772E-02
MAST4	0.894	1.859	4.650E-04	8.823E-03
ZFAND2A	0.899	1.865	4.370E-06	2.341E-04
ZNF628	0.900	1.866	2.684E-03	3.222E-02
ASMTL-AS1	0.902	1.869	8.460E-06	3.950E-04
ZBTB20	0.907	1.875	1.534E-03	2.146E-02
CYP4V2	0.908	1.877	1.304E-04	3.449E-03
SETD1B	0.912	1.881	3.430E-10	6.580E-08
NXT1	0.912	1.881	3.860E-05	1.348E-03
IL6ST	0.912	1.882	5.010E-05	1.654E-03
ENSG00000269688	0.912	1.882	8.641E-04	1.395E-02
CTSK	0.915	1.885	5.841E-04	1.052E-02
GLUD1P3	0.915	1.886	4.271E-03	4.417E-02
SCNN1D	0.916	1.887	9.470E-05	2.687E-03
ENSG00000232545	0.917	1.888	5.076E-03	4.966E-02
ZXDA	0.917	1.888	1.152E-04	3.141E-03
KLHL11	0.919	1.891	1.652E-03	2.256E-02
SNIP1	0.920	1.893	3.196E-04	6.754E-03
EDAR	0.922	1.895	1.294E-03	1.899E-02
ABCD2	0.926	1.900	1.365E-04	3.547E-03
ZNF256	0.927	1.901	2.885E-04	6.312E-03
ANTXRLP1	0.927	1.902	1.811E-03	2.410E-02
WDR74	0.929	1.904	9.700E-05	2.734E-03
GPRASP1	0.930	1.905	4.769E-04	8.968E-03
LINC00852	0.930	1.905	6.850E-05	2.074E-03
FASTKD5	0.931	1.907	6.630E-07	4.720E-05
MOAP1	0.933	1.909	4.620E-09	6.270E-07
TADA1	0.936	1.913	3.640E-05	1.289E-03
PPIF	0.936	1.913	3.520E-06	1.973E-04
MATR3	0.938	1.916	2.239E-03	2.826E-02
PDE4B	0.940	1.918	2.000E-05	8.122E-04
NLRP6	0.940	1.919	5.945E-04	1.063E-02
RAB33B-AS1	0.945	1.925	3.465E-03	3.830E-02
DBF4	0.946	1.927	2.925E-04	6.362E-03
IRF2BP2	0.947	1.928	1.140E-09	1.810E-07
CCNT1	0.951	1.933	5.740E-10	9.970E-08
SNPH	0.951	1.933	4.021E-03	4.246E-02
YRDC	0.953	1.936	5.730E-06	2.887E-04
FEM1C	0.954	1.937	2.130E-06	1.266E-04
LINC00641	0.959	1.945	2.500E-05	9.710E-04
CCNL1	0.961	1.947	1.930E-09	2.870E-07
ZNF101	0.961	1.947	1.640E-05	6.936E-04
MIRLET7A1HG	0.963	1.949	2.058E-04	4.963E-03
CDR2	0.963	1.950	1.849E-04	4.551E-03
ZNF639	0.964	1.951	8.050E-09	1.040E-06
DOC2GP	0.965	1.952	1.507E-03	2.115E-02
DCTN6	0.967	1.955	1.254E-03	1.847E-02
DNAJB9	0.968	1.957	3.380E-05	1.215E-03
CREBRF	0.973	1.963	6.470E-09	8.560E-07
TBKBP1	0.978	1.970	6.293E-04	1.109E-02
CCDC85C	0.980	1.972	2.530E-03	3.092E-02
MAFK	0.984	1.979	1.150E-06	7.630E-05
SLC22A23	0.985	1.979	1.410E-06	8.930E-05
DUSP5	0.993	1.990	6.290E-05	1.950E-03
SNHG8	1.001	2.002	6.127E-04	1.087E-02
MIR23AHG	1.002	2.003	1.224E-03	1.820E-02
ENSG00000259623	1.006	2.008	1.670E-05	6.989E-04
BCL11B	1.008	2.011	3.290E-08	3.460E-06
LTBP3	1.010	2.013	2.205E-03	2.792E-02
MTFP1	1.012	2.017	3.627E-03	3.971E-02
NDUFAF5	1.014	2.019	1.061E-03	1.633E-02
RORA	1.023	2.033	2.218E-04	5.237E-03
TWF1	1.027	2.037	1.270E-08	1.500E-06
SYCP2	1.029	2.040	1.876E-03	2.471E-02
ENSG00000257497	1.029	2.041	1.512E-03	2.118E-02
RNF139	1.029	2.041	3.700E-05	1.309E-03
NR4A1	1.030	2.042	1.180E-05	5.299E-04
DHRS3	1.035	2.049	2.316E-03	2.886E-02
ENSG00000274460	1.037	2.052	2.306E-03	2.876E-02
NDST2	1.038	2.054	3.160E-03	3.592E-02
SHLD3	1.040	2.057	6.730E-05	2.057E-03
SPEG	1.041	2.058	2.326E-03	2.894E-02
ENSG00000255182	1.045	2.063	1.194E-04	3.237E-03
SNHG15	1.048	2.068	7.240E-11	1.700E-08
MIR22HG	1.052	2.074	2.105E-04	5.024E-03
ACTA2-AS1	1.054	2.076	3.290E-03	3.689E-02
ENSG00000223511	1.063	2.089	4.530E-05	1.530E-03
TSPYL2	1.064	2.090	2.260E-09	3.250E-07
SEC14L2	1.069	2.098	1.593E-04	4.010E-03
TUBB2A	1.071	2.102	5.035E-03	4.938E-02
NAF1	1.072	2.103	5.360E-06	2.746E-04
NCR3LG1	1.074	2.105	2.084E-03	2.673E-02
ENSG00000265625	1.079	2.113	4.492E-03	4.577E-02
PGGHG	1.083	2.119	5.427E-04	9.893E-03
WHAMM	1.083	2.119	8.080E-06	3.835E-04
PRR7	1.084	2.120	1.139E-03	1.725E-02
IKZF5	1.084	2.120	5.300E-13	2.050E-10
ENSG00000260404	1.085	2.122	3.780E-06	2.072E-04
CD69	1.085	2.122	9.960E-09	1.230E-06
ITPRIP	1.088	2.126	3.590E-08	3.720E-06
IER3	1.090	2.129	3.788E-03	4.074E-02
C6orf226	1.090	2.129	7.808E-04	1.300E-02
FAM86B3P	1.095	2.135	1.933E-03	2.527E-02
ADGRB1	1.101	2.145	2.540E-03	3.100E-02
NA	1.104	2.149	3.240E-04	6.815E-03
RBM34	1.105	2.150	3.665E-03	3.999E-02
AFAP1	1.105	2.151	3.408E-03	3.785E-02
LINC01089	1.111	2.159	5.620E-06	2.838E-04
ENSG00000269951	1.120	2.174	4.229E-03	4.389E-02
EML5	1.123	2.177	4.451E-03	4.550E-02
PASK	1.127	2.183	1.010E-05	4.609E-04
ARRDC3	1.133	2.193	4.308E-04	8.445E-03
TOE1	1.133	2.194	1.010E-11	2.960E-09
RBM38	1.137	2.199	6.240E-07	4.480E-05
PIGA	1.144	2.210	1.900E-06	1.160E-04
ENSG00000258944	1.146	2.213	2.697E-04	5.994E-03
TRBV30	1.147	2.214	1.401E-03	2.012E-02
YOD1	1.155	2.227	7.340E-11	1.700E-08
ENSG00000255026	1.160	2.235	7.886E-04	1.306E-02
OSER1	1.162	2.238	6.670E-18	5.550E-15
IGLV6-57	1.163	2.239	1.796E-03	2.396E-02
CCDC59	1.164	2.240	2.610E-08	2.800E-06
CHASERR	1.169	2.249	5.680E-12	1.750E-09
LINC00653	1.169	2.249	5.070E-06	2.633E-04
SIAH1	1.173	2.254	3.080E-07	2.510E-05
BEX2	1.179	2.264	7.620E-09	9.910E-07
CLN8-AS1	1.180	2.266	2.719E-03	3.246E-02
ENSG00000273253	1.183	2.270	4.109E-04	8.147E-03
LZTS3	1.183	2.271	3.970E-05	1.381E-03
DDX47	1.184	2.272	1.040E-09	1.690E-07
TENT5C	1.186	2.275	2.070E-07	1.850E-05
EIF4A1	1.186	2.275	5.590E-10	9.810E-08
CACNA1H	1.187	2.276	5.840E-05	1.867E-03
CLP1	1.190	2.282	3.870E-07	3.010E-05
DUSP10	1.192	2.285	6.390E-06	3.131E-04
CDKN1B	1.196	2.291	2.230E-08	2.450E-06
ZBTB21	1.204	2.303	5.340E-09	7.110E-07
DUS3L	1.206	2.307	1.670E-07	1.520E-05
NAP1L5	1.211	2.315	1.250E-04	3.353E-03
SLC7A5	1.219	2.328	1.430E-07	1.330E-05
SIK1B	1.222	2.333	3.780E-06	2.072E-04
ERFL	1.223	2.334	4.521E-04	8.652E-03
SNORD4A	1.232	2.349	1.780E-03	2.376E-02
CBX4	1.232	2.349	1.650E-12	5.500E-10
JUNB	1.234	2.352	1.660E-06	1.031E-04
C1R	1.239	2.360	2.424E-03	2.982E-02
ADAMTS7P1	1.240	2.362	1.664E-03	2.271E-02
CXCR4	1.249	2.376	2.900E-13	1.240E-10
RBM38-AS1	1.250	2.378	1.902E-03	2.497E-02
CSRNP1	1.250	2.379	3.480E-12	1.090E-09
PTP4A1	1.255	2.387	2.170E-08	2.400E-06
SPATA2	1.257	2.391	1.080E-08	1.330E-06
ZNF551	1.259	2.392	2.350E-10	4.960E-08
ZNF367	1.263	2.399	5.340E-05	1.737E-03
HBEGF	1.268	2.408	7.978E-04	1.317E-02
DNAJB1	1.270	2.411	9.110E-19	8.930E-16
ENSG00000280138	1.273	2.417	5.040E-19	5.250E-16
ARRDC4	1.278	2.425	1.640E-06	1.019E-04
PELI1	1.282	2.431	5.520E-10	9.790E-08
CTSF	1.286	2.438	2.097E-04	5.021E-03
RNF103	1.294	2.452	1.670E-08	1.870E-06
BNC2	1.298	2.460	4.575E-03	4.625E-02
VEGFA	1.299	2.461	1.303E-03	1.907E-02
POLR1F	1.305	2.471	8.340E-14	4.020E-11
SOX4	1.306	2.472	9.710E-09	1.210E-06
HIF1A	1.307	2.475	4.400E-10	8.050E-08
ENSG00000224505	1.308	2.477	1.838E-03	2.436E-02
JUND	1.313	2.484	9.570E-09	1.200E-06
FOSB	1.313	2.485	6.710E-11	1.620E-08
MIR590	1.314	2.486	1.505E-03	2.114E-02
NR3C2	1.325	2.505	4.103E-03	4.302E-02
PLK3	1.325	2.505	1.350E-09	2.100E-07
IL12A	1.325	2.506	3.290E-03	3.689E-02
SNHG9	1.329	2.511	8.253E-04	1.346E-02
COL18A1	1.340	2.531	1.042E-03	1.610E-02
ZNF331	1.340	2.531	1.390E-06	8.870E-05
ADAMTS17	1.346	2.542	1.707E-04	4.253E-03
RPS6KL1	1.348	2.546	1.230E-03	1.824E-02
TAS1R3	1.350	2.549	2.310E-06	1.354E-04
IL23A	1.359	2.565	3.190E-05	1.165E-03
ENSG00000233264	1.360	2.567	7.080E-05	2.121E-03
C17orf49	1.364	2.574	8.500E-09	1.080E-06
PTCH1	1.367	2.580	4.969E-03	4.889E-02
ENSG00000256152	1.385	2.612	2.891E-04	6.312E-03
ZNF394	1.392	2.625	4.330E-13	1.760E-10
HDGFL3	1.397	2.634	4.760E-05	1.584E-03
LINC02390	1.404	2.646	1.660E-05	6.972E-04
SBDSP1	1.409	2.655	8.210E-21	1.140E-17
SLC2A3	1.410	2.657	2.500E-07	2.110E-05
PFKFB3	1.412	2.661	3.230E-11	8.030E-09
NTN5	1.413	2.662	1.366E-03	1.974E-02
NSG1	1.421	2.678	5.188E-04	9.551E-03
SBDS	1.425	2.685	6.640E-13	2.510E-10
SOCS3	1.428	2.691	4.960E-06	2.588E-04
DDIT3	1.435	2.703	3.840E-16	2.560E-13
ZNF14	1.457	2.744	7.420E-10	1.210E-07
FBXO33	1.457	2.744	5.270E-20	6.270E-17
OTUD7A	1.460	2.750	1.040E-06	6.980E-05
DFFBP1	1.464	2.758	2.560E-05	9.866E-04
SDE2	1.464	2.759	1.150E-17	8.730E-15
PDE4D	1.468	2.766	4.040E-09	5.600E-07
CTLA4	1.481	2.791	9.951E-04	1.557E-02
RN7SL2	1.481	2.792	1.260E-05	5.585E-04
EIF2AK3-DT	1.482	2.793	1.190E-08	1.430E-06
FOSL2	1.487	2.804	4.440E-17	3.220E-14
SNORA75	1.491	2.812	2.518E-03	3.082E-02
CITED4	1.494	2.816	2.000E-05	8.122E-04
NDUFV2	1.496	2.820	8.150E-12	2.420E-09
PIM3	1.500	2.829	9.130E-29	5.070E-25
IGLV4-60	1.507	2.843	3.758E-03	4.050E-02
GADD45A	1.519	2.866	2.780E-10	5.590E-08
ENSG00000229299	1.519	2.867	6.000E-06	2.991E-04
B3GNT7	1.524	2.876	7.980E-07	5.560E-05
EGR1	1.538	2.905	9.542E-04	1.514E-02
ENSG00000279520	1.540	2.907	2.340E-09	3.340E-07
RAB3A	1.541	2.910	1.948E-03	2.540E-02
LINC02033	1.542	2.912	4.793E-03	4.757E-02
ENSG00000275056	1.546	2.921	1.135E-03	1.721E-02
ENSG00000228201	1.553	2.935	5.026E-04	9.348E-03
RSPH4A	1.560	2.948	4.216E-03	4.382E-02
HAR1A	1.562	2.954	7.110E-04	1.209E-02
NFKBIA	1.571	2.971	1.490E-09	2.300E-07
FGFR1	1.573	2.976	1.040E-06	6.980E-05
WDR86	1.578	2.986	9.610E-06	4.400E-04
SCARF2	1.580	2.989	3.076E-03	3.537E-02
JUN	1.589	3.008	1.260E-21	2.090E-18
CEROX1	1.593	3.016	4.580E-07	3.470E-05
RPL13P12	1.596	3.023	1.579E-04	3.997E-03
ENSG00000260708	1.596	3.023	2.880E-12	9.230E-10
BCL3	1.613	3.060	1.080E-09	1.720E-07
MMP28	1.618	3.071	7.460E-04	1.254E-02
LINC00304	1.630	3.096	3.968E-04	7.966E-03
MYRF	1.634	3.103	8.451E-04	1.370E-02
ENSG00000276517	1.640	3.117	8.122E-04	1.333E-02
TNRC18P1	1.649	3.137	1.341E-04	3.513E-03
FAM183BP	1.650	3.138	1.392E-03	2.004E-02
IRS2	1.653	3.144	1.170E-09	1.840E-07
TRPM5	1.690	3.227	3.522E-03	3.881E-02
ENSG00000276853	1.695	3.238	2.018E-03	2.612E-02
MTSS2	1.697	3.243	9.540E-11	2.150E-08
RBKS	1.719	3.292	1.027E-03	1.591E-02
ENSG00000255847	1.723	3.301	3.640E-13	1.520E-10
ENSG00000273306	1.752	3.367	1.572E-04	3.991E-03
ENC1	1.759	3.384	3.320E-07	2.670E-05
ENSG00000279908	1.775	3.423	2.420E-09	3.410E-07
FAM153A	1.799	3.480	6.660E-05	2.041E-03
ENSG00000232891	1.804	3.492	4.911E-04	9.204E-03
BTG3	1.827	3.547	1.400E-11	3.880E-09
ZBTB10	1.829	3.552	2.450E-26	5.830E-23
KCNK7	1.835	3.568	9.350E-06	4.291E-04
ZC3H12A	1.840	3.580	9.790E-28	4.080E-24
EGR2	1.848	3.599	1.150E-05	5.160E-04
KLF5	1.849	3.601	1.630E-05	6.907E-04
TNFAIP3	1.853	3.612	1.110E-08	1.340E-06
ENSG00000279765	1.859	3.627	7.840E-05	2.309E-03
CCL3L3	1.886	3.696	3.710E-05	1.310E-03
CFL2	1.895	3.719	1.500E-12	5.090E-10
TTC34	1.896	3.721	1.450E-05	6.245E-04
CRABP2	1.915	3.771	1.857E-03	2.455E-02
TP53INP2	1.918	3.778	1.310E-15	7.510E-13
UPK3B	1.928	3.805	1.020E-06	6.930E-05
RGS17P1	1.929	3.808	3.560E-05	1.267E-03
LMTK3	1.930	3.811	3.880E-07	3.010E-05
KRT18	1.933	3.820	6.919E-04	1.184E-02
PSMD10P1	1.980	3.944	2.660E-07	2.230E-05
OSM	1.982	3.952	3.330E-05	1.202E-03
DUSP2	2.021	4.060	3.140E-11	7.920E-09
ENSG00000261159	2.031	4.086	1.614E-03	2.228E-02
NA	2.035	4.099	4.230E-05	1.447E-03
MAFF	2.085	4.242	1.260E-08	1.500E-06
LRRC32	2.090	4.256	7.562E-04	1.266E-02
RHBDL1	2.123	4.357	1.977E-04	4.799E-03
SNAI1	2.148	4.432	9.033E-04	1.444E-02
AREG	2.166	4.489	4.989E-04	9.308E-03
ENSG00000272256	2.171	4.504	1.440E-05	6.211E-04
ENSG00000267655	2.203	4.603	1.972E-03	2.567E-02
FAM153B	2.206	4.614	3.677E-04	7.499E-03
RND1	2.213	4.635	7.490E-04	1.258E-02
RPL23AP81	2.261	4.794	4.746E-04	8.955E-03
FFAR1	2.265	4.806	3.720E-18	3.440E-15
NA	2.267	4.812	2.350E-10	4.960E-08
ENSG00000245869	2.314	4.971	5.380E-06	2.748E-04
DIP2A-IT1	2.339	5.059	1.960E-06	1.186E-04
MATN1	2.339	5.061	3.210E-05	1.169E-03
ENSG00000231305	2.357	5.122	7.635E-04	1.275E-02
ENSG00000256712	2.367	5.157	2.747E-03	3.269E-02
GRM2	2.392	5.248	5.290E-09	7.110E-07
MIR4420	2.399	5.274	2.860E-06	1.639E-04
CXCL8	2.400	5.279	7.380E-10	1.210E-07
ENSG00000207525	2.410	5.316	3.989E-04	7.997E-03
RBBP4P1	2.416	5.336	3.657E-03	3.995E-02
SULT1A2	2.435	5.406	9.660E-07	6.570E-05
TCTE1	2.442	5.433	5.029E-03	4.935E-02
AVPI1	2.443	5.440	8.720E-05	2.508E-03
HLA-U	2.444	5.440	1.685E-03	2.288E-02
ENSG00000275927	2.458	5.494	1.932E-03	2.527E-02
EREG	2.501	5.660	5.270E-05	1.724E-03
LOC105378721	2.524	5.750	6.651E-04	1.153E-02
TNFSF9	2.532	5.785	8.500E-05	2.459E-03
FLT4	2.538	5.807	6.380E-05	1.973E-03
CHMP4BP1	2.538	5.808	6.960E-05	2.100E-03
DUSP4	2.605	6.083	3.700E-06	2.048E-04
ENSG00000274677	2.653	6.288	3.481E-04	7.214E-03
ENSG00000278022	2.676	6.390	4.150E-05	1.425E-03
ENSG00000256913	2.717	6.576	4.902E-04	9.198E-03
NR4A3	2.718	6.580	1.740E-07	1.570E-05
PER1	2.758	6.765	6.470E-21	9.800E-18
TAMALIN	2.791	6.921	3.640E-45	6.070E-41
CD83	2.850	7.210	2.750E-24	5.100E-21
FAM238A	2.902	7.473	2.770E-16	1.920E-13
ADRB1	2.906	7.496	2.030E-05	8.221E-04
DUSP8	2.981	7.895	8.650E-06	4.024E-04
THNSL2	3.049	8.277	3.100E-03	3.557E-02
ENSG00000270681	3.108	8.624	3.192E-04	6.754E-03
ACTN1-AS1	3.126	8.732	4.670E-06	2.452E-04
FOSL1	3.150	8.877	4.726E-03	4.716E-02
TPBGL	3.156	8.914	4.160E-03	4.334E-02
CHRM4	3.189	9.120	2.280E-06	1.340E-04
TMEM119	3.276	9.684	1.179E-03	1.772E-02
NR4A2	3.339	10.116	7.350E-18	5.830E-15
GRK5-IT1	3.339	10.117	1.958E-04	4.771E-03
MMP9	3.528	11.532	8.140E-06	3.841E-04
GEM	3.551	11.718	4.821E-03	4.776E-02
G0S2	3.560	11.793	6.010E-16	3.850E-13
SAXO2	3.803	13.956	6.210E-26	1.290E-22
HAR1B	3.908	15.007	5.480E-05	1.775E-03
ABHD17AP6	3.928	15.221	3.253E-03	3.665E-02
ENSG00000259635	4.089	17.022	4.475E-04	8.610E-03
IL13	4.111	17.283	2.698E-04	5.994E-03
SLED1	4.143	17.665	3.646E-03	3.986E-02
TEX45	5.004	32.082	4.948E-04	9.244E-03
YAP1P1	5.831	56.906	3.275E-04	6.864E-03
CLLU1-AS1	5.963	62.401	9.653E-04	1.527E-02
ENSG00000224029	6.334	80.649	4.380E-05	1.492E-03
RPS4XP22	6.439	86.769	2.270E-03	2.851E-02
LRRC7	6.948	123.489	1.091E-04	3.014E-03
RASD2	6.974	125.745	2.660E-05	1.008E-03
TUBB8B	6.974	125.745	2.660E-05	1.008E-03
MTCO3P12	8.403	338.535	1.370E-05	5.963E-04
ZFP57	8.785	441.159	2.190E-09	3.180E-07

Patients with AD and HCs were stratified by age (0‒6 months and 7‒12 months), and differential expression analysis on RNA-seq data was performed between the four groups: children with AD aged >6 months above versus age-matched HCs, children with AD aged <6 months versus age-matched HCs, patients with AD aged >6 months versus patients with AD aged <6 months, and HCs aged >6 months versus HCs aged <6 months. Meeting criteria were set at FC ≥1.5 and FDR <0.05.

Abbreviations: AD, atopic dermatitis; FC, fold change; FDR, false discovery rate; HC, healthy control; KLK, kallikrein; MMP, matrix metalloproteinase; RNA-seq, RNA sequencing; STAT, signal transducer and activator of transcription; TLR, toll-like receptor.

**Table 4:** Effect of Infant’s Age on Validated DEG in Infants with AD

DEG	Group	LFC	FC	FDR
*IL18RAP*	Patients with AD aged >6 mo versus HCs	‒1.968	0.256	0.003
	Patients with AD aged <6 mo versus HCs	‒1.309	0.404	0.043
*IL1B*	Patients with AD aged <6 mo versus HCs	3.901	14.942	0.000
*TNF*	Patients with AD aged <6 mo versus HCs	3.092	8.526	0.000
*TREM1*	Patients with AD aged <6 mo versus HCs	3.164	8.963	0.000
*EGR3*	Patients with AD aged <6 mo versus HCs	5.076	33.732	0.015

Shown is the effect of infant’s age on the expression of selected genes that were differentially expressed in infants with AD and validated by RT-qPCR. Differential expression analysis between patients with AD and HCs was performed using DESeq2 on RNA-seq data after stratifying the dataset by age (0‒6 months and 7‒12 months).

Abbreviations: AD, atopic dermatitis; AIF, apoptosis-inducing factor*;* DEG, differentially expressed gene; FC, fold change; FDR, false discovery rate; HC, healthy control; LFC, log_2_ fold change; OSM, oncostatin M; RNA-seq, RNA sequencing; XAF1, XIAP-associated factor 1.

### Gene ontology and pathway analysis of DEGs

**Figure 4:**
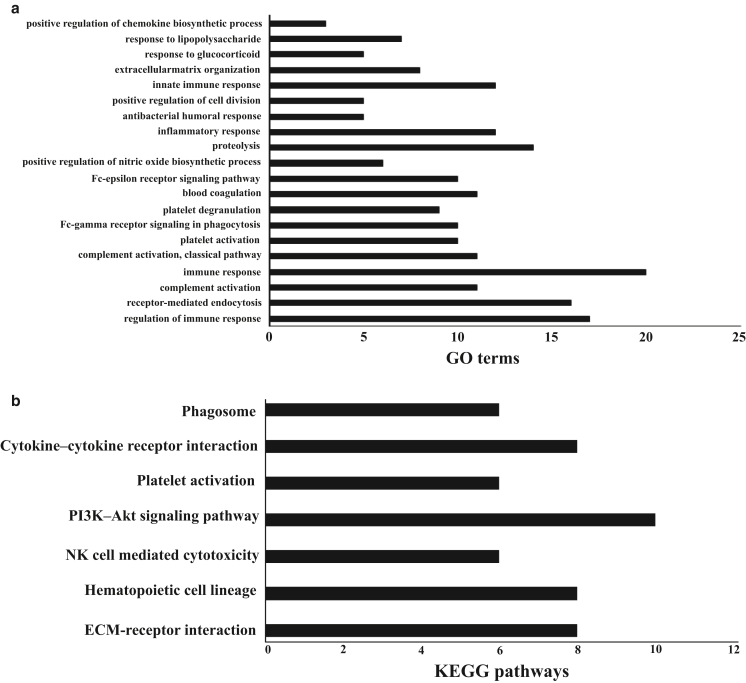
**GO enrichment and pathway analysis of differentially expressed genes.** (**a**) The top 20 enriched GO terms; the x-axis represents gene counts, and the y-axis represents GO terms. (**b**) Selected KEGG pathways; the x-axis represents gene counts, and the y-axis represents KEGG pathway names. Akt, protein kinase B; ECM, extracellular matrix; GO, gene ontology; KEGG, Kyoto Encyclopedia of Genes and Genomes; PI3K, phosphoinositide 3-kinase.

To gain insight into the gene ontology (GO) categories of DEGs between the AD group and control group, all DEGs were uploaded to the DAVID (Database for Annotation, Visualization and Integrated Discovery) database. GO analysis contained three categories: biological process (BP), cellular component (CC), and molecular function (MF). In total, 86 significant GO terms were enriched for DEGs identified in the AD group, of which 57 were within the BP category, 21 were within the CC category, and 8 were within the MF category. The enriched BP categories included immune response, inflammatory response, regulation of immune response, leukocyte migration, positive regulation of NF-kB transcription factor activity, cell adhesion, cell surface receptor signaling pathway, and many others. In the category CC, the DEGs were significantly enriched in the plasma membrane, extracellular region, extracellular exosome, cell surface, and cytoplasm. Furthermore, in the category MF, DEGs were mainly enriched in antigen binding, receptor activity, cytokine activity, enzyme binding, and protein binding. The selected pathways significantly enriched in the AD group included hematopoietic cell lineage, NK cell‒mediated cytotoxicity, phosphoinositide 3-kinase‒protein kinase B signaling pathway, cytokine‒cytokine receptor interaction, extracellular matrix‒receptor interaction, and immunoregulatory interactions between a lymphoid and a nonlymphoid cell (Figure 4a and b). Further details of the results of the GO enrichment and pathway analyses are provided in Table 5.

**Table 5:** GO Enrichment and Pathway Analyses of Differentially Expressed Genes

GO Term	Gene Count	*P*-Value	Genes
Biological process			
Regulation of immune response	17	3.76E-12	*ICAM1*, *IGLV1-44*, *IGKV5-2*, *NCR1*, *IGLV2-11*, *IGLV2-8*, *IGLV3-19*, *IGKV1-5*, *IGLV2-23*, *IGKV4-1*, *KLRF1*,*TREM1*, *KIR2DL3*, *TREML1*, *IGLC2*, *KLRD1*, and *IGKV3-15*
Receptor-mediated endocytosis	16	8.68E-11	*LDLR*, *IGLV1-44*, *IGKV5-2*, *JCHAIN*, *SPARC*, *IGLV2-11*, *IGLV2-8*, *IGLV3-19*, *CTTN*, *IGKV1-5*, *IGLV2-23*, *IGKV4-1*, *IGLC2*, *LRP3*, *CD14*, and *IGKV3-15*
Complement activation	11	4.37E-09	*IGLV2-11*, *IGLV2-8*, *IGLV3-19*, *IGKV1-5*, *IGLV1-44*, *IGLV2-23*, *IGKV5-2*, *CLU*, *IGKV4-1*, *IGLC2*, and *IGKV3-15*
Immune response	20	4.97E-09	*IL18R1*, *TNF*, *IL18RAP*, *CXCL5*, *IGLV1-44*, *IGKV5-2*, *JCHAIN*, *FASLG*, *PF4*, *IGLV2-11*, *IGLV2-8*, *IGLV3-19*, *IGKV1-5*, *PPBP*, *FCAR*, *IGLV2-23*, *IGKV4-1*, *IL1B*, *KIR2DL3*, and *IGKV3-15*
Complement activation and classical pathway	11	1.56E-08	*IGLV2-11*, *IGLV2-8*, *IGLV3-19*, *IGKV1-5*, *IGLV1-44*, *IGLV2-23*, *IGKV5-2*, *CLU*, *IGKV4-1*, *IGLC2*, and *IGKV3-15*
Platelet activation	10	7.55E-07	*VWF*, *GP6*, *F5*, *C6ORF25*, *PF4*, *GP1BA*, *ITGB3*, *TREML1*, *CLEC1B*, and *GP9*
Fc-γ receptor signaling pathway involved in phagocytosis	10	1.74E-06	*IGLV2-11*, *IGLV2-8*, *IGLV3-19*, *IGKV1-5*, *IGLV1-44*, *IGLV2-23*, *IGKV5-2*, *IGKV4-1*, *IGLC2*, and *IGKV3-15*
Platelet degranulation	9	3.36E-06	*VWF*, *APP*, *F5*, *PPBP*, *CLU*, *PF4*, *SPARC*, *ITGB3*, and *ITGA2B*
Blood coagulation	11	5.08E-06	*PRKAR2B*, *VWF*, *GP6*, *F5*, *C6ORF25*, *PDGFC*, *GP1BA*, *PDGFD*, *ITGB3*, *GP9*, and *PLAUR*
FCERI signaling pathway	10	2.71E-05	*IGLV2-11*, *IGLV2-8*, *IGLV3-19*, *IGKV1-5*, *IGLV1-44*, *IGLV2-23*, *IGKV5-2*, *IGKV4-1*, *IGLC2*, and *IGKV3-15*
Positive regulation of nitric oxide biosynthetic process	6	3.54E-05	*ICAM1*, *TNF*, *CLU*, *IL1B*, *PTX3*, and *KLF4*
Proteolysis	14	0.000	*NRIP3*, *IGLV1-44*, *IGKV5-2*, *ANPEP*, *MMP25*, *IGLV2-11*, *IGKV1-5*, *IGLV3-19*, *IGLV2-8*, *F5*, *IGLV2-23*, *IGKV4-1*, *IGLC2*, and *IGKV3-15*
Inflammatory response	12	0.001	*SDC1*, *TNF*, *IL18RAP*, *PPBP*, *CXCL5*, *ANXA1*, *IL1B*, *PF4*, *PTX3*, *NLRP3*, *CD14*, and *MMP25*
Antibacterial humoral response	5	0.001	*APP*, *ADM*, *HIST2H2BE*, *HIST1H2BJ*, and *JCHAIN*
Positive regulation of cell division	5	0.001	*TAL1*, *PPBP*, *IL1B*, *PDGFC*, and *PDGFD*
Innate immune response	12	0.001	*APP*, *CLU*, *ANXA1*, *JCHAIN*, *PADI4*, *TREM1*, *PTX3*, *NLRP3*, *IGLC2*, *TREML1*, *KLRD1*, and *CD14*
ECM organization	8	0.002	*ICAM1*, *VWF*, *APP*, *TNF*, *ITGB5*, *SPARC*, *ITGB3*, and *ITGA2B*
Response to glucocorticoid	5	0.003	*SDC1*, *TNF*, *ADM*, *SPARC*, and *ADAM9*
Response to lipopolysaccharide	7	0.003	*PPBP*, *ADM*, *CXCL5*, *FASLG*, *PF4*, *SPARC*, and *TRIB1*
Positive regulation of chemokine biosynthetic process	3	0.003	*EGR1*, *TNF*, and *IL1B*
Negative regulation of extrinsic apoptotic signaling pathway in absence of ligand	4	0.004	*TNF*, *MCL1*, *IL1B*, and *PF4*
Viral entry into host cell	5	0.005	*ICAM1*, *LDLR*, *ITGB5*, *ANPEP*, and *ITGB3*
Response to yeast	3	0.006	*APP*, *ADM*, and *PTX3*
Defense response to Gram-positive bacterium	5	0.007	*APP*, *TNF*, *ADM*, *HIST2H2BE*, and *HIST1H2BJ*
Negative regulation of gene expression	6	0.007	*TNF*, *LDLR*, *GAS2L1*, *ZNF503*, *MYADM*, and *KLF4*
Cell adhesion mediated by integrin	3	0.007	*ICAM1*, *ITGB3*, and *ADAM9*
Positive regulation of membrane protein ectodomain proteolysis	3	0.007	*TNF*, *IL1B*, and *ADAM9*
Positive regulation of IFN-γ production	4	0.008	*IL18R1*, *TNF*, *IL1B*, and *CD14*
Cell surface receptor signaling pathway	8	0.01	*IL18RAP*, *ANXA1*, *GP1BA*, *TSPAN9*, *KLRF1*, *KLRD1*, *CD14*, and *CLEC1B*
Blood coagulation, intrinsic pathway	3	0.011	*VWF*, *GP1BA*, and *GP9*
Positive regulation of leukocyte chemotaxis	3	0.011	*PPBP*, *CXCL5*, and *PF4*
Integrin-mediated signaling pathway	5	0.011	*C6ORF25*, *ITGB5*, *ITGB3*, *ADAM9*, and *ITGA2B*
Platelet formation	3	0.012	*TAL1*, *C6ORF25*, and *CLEC1B*
Positive regulation of transcription from RNA polymerase II promoter	17	0.012	*EGR1*, *NAMPT*, *TNF*, *EGR2*, *ABLIM3*, *PF4*, *MYBL1*, *NLRP3*, *AHR*, *TAL1*, *APP*, *SPX*, *BHLHA15*, *IL1B*, *MAML3*, *FOSL1*, and *KLF4*
Cellular response to hydrogen peroxide	4	0.014	*IL18RAP*, *ANXA1*, *PDGFD*, and *KLF4*
Positive regulation of NF-kB import into the nucleus	3	0.014	*IL18R1*, *TNF*, and *IL1B*
Positive regulation of MAPK activity	4	0.015	*TNF*, *PDE5A*, *PDGFC*, and *PDGFD*
Positive regulation of smooth muscle cell proliferation	4	0.016	*NAMPT*, *TNF*, *PDGFD*, and *ALOX12*
Cellular response to lipopolysaccharide	5	0.017	*ICAM1*, *TNF*, *NLRP3*, *CD14*, and *ADAM9*
Regulation of gastric acid secretion	2	0.017	*SGK1* and *KCNQ1*
Cell adhesion	10	0.02	*ICAM1*, *VWF*, *APP*, *SCN1B*, *ITGB5*, *GP1BA*, *ITGB3*, *ADAM9*, *GP9*, and *ITGA2B*
Leukocyte migration	5	0.022	*ICAM1*, *GP6*, *ESAM*, *TREM1*, and *ITGB3*
Regulation of cell proliferation	6	0.024	*TAL1*, *SGK1*, *TNF*, *PPBP*, *ANXA1*, and *PF4*
Establishment or maintenance of microtubule cytoskeleton polarity	2	0.026	*KIF2C* and *LMNA*
positive regulation of calcidiol 1-monooxygenase activity	2	0.026	*TNF* and *IL1B*
Positive regulation of phagocytosis	3	0.026	*TNF*, *IL1B*, and *PTX3*
Positive regulation of gene expression	7	0.028	*TNF*, *LDLR*, *ID1*, *IL1B*, *PF4*, *KLF4*, and *ALOX12*
Positive regulation of NF-kB transcription factor activity	5	0.03	*ICAM1*, *TNF*, *CLU*, *IL1B*, and *NLRP3*
Lipopolysaccharide-mediated signaling pathway	3	0.032	*TNF*, *IL1B*, and *CD14*
Sequestering of triglyceride	2	0.035	*TNF* and *IL1B*
IL-1 beta production	2	0.043	*IL1B* and *NLRP3*
Positive regulation of fever generation	2	0.043	*TNF* and *IL1B*
Cell matrix adhesion	4	0.044	*ITGB5*, *ITGB3*, *ADAM9*, and *ITGA2B*
TGFβ receptor signaling pathway	4	0.047	*ID1*, *CLDN5*, *ITGB5*, and *ADAM9*
Positive regulation of cysteine-type endopeptidase activity involved in apoptotic process	3	0.048	*TNF*, *NLRP3*, and *ALOX12*
Positive regulation of apoptotic process	7	0.049	*TNF*, *ADM*, *CLU*, *FASLG*, *FOSL1*, *MELK*, and *DUSP6*
Platelet aggregation	3	0.049	*GP1BA*, *ITGB3*, and *ITGA2B*
Cellular component			
Plasma membrane	68	4.45E-09	*SEPT5*, *IGLV1-44*, *LDLR*, *C6ORF25*, *CLDN5*, *FASLG*, *IGKV1-12*, *TSPAN9*, *MMP25*, *GLDC*, *GP9*, *ATP2B1*, *PRKAR2B*, *CTTN*, *APP*, *GP6*, *HOMER3*, *IGLV2-23*, *ZNF185*, *DLG3*, *PDGFC*, *ESAM*, *FAM129B*, *KLRD1*, *KCNQ1*, *ICAM1*, *SGK1*, *IL18RAP*, *SLCO4A1*, *WLS*, *NCR1*, *MYADM*, *PLAUR*, *IGLV2-11*, *IGKV1-5*, *SDC1*, *F5*, *COLQ*, *IGKV4-1*, *TREM1*, *KIR2DL3*, *EMP1*, *MELK*, *CLEC1B*, *ITGA2B*, *TNF*, *SCN1B*, *CALD1*, *IGKV5-2*, *ITGB5*, *GNG11*, *ITGB3*, *C2ORF88*, *IGLV2-8*, *IGLV3-19*, *GP1BA*, *KLRF1*, *IGKV3-15*, *IL18R1*, *ANXA1*, *SPARC*, *RAPH1*, *AQP10*, *FCAR*, *CPNE2*, *IGLC2*, *TREML1*, and *CD14*
Extracellular region	34	1.16E-06	*LTBP1*, *SCN1B*, *TNF*, *IGLV1-44*, *CXCL5*, *IGKV5-2*, *CLU*, *JCHAIN*, *FASLG*, *PF4*, *IGKV1-12*, *IGLV2-8*, *APP*, *IGLV3-19*, *IGLV2-23*, *IL1B*, *PDGFC*, *PDGFD*, *PTX3*, *IGKV3-15*, *ANXA1*, *SPARC*, *NLRP3*, *IGLV2-11*, *VWF*, *IGKV1-5*, *F5*, *ADM*, *FCAR*, *PPBP*, *IGKV4-1*, *TREM1*, *IGLC2*, and *CD14*
Extracellular space	30	2.25E-06	*NAMPT*, *TNF*, *CXCL5*, *FAM20C*, *CLU*, *JCHAIN*, *FASLG*, *PF4*, *ANPEP*, *APP*, *SPX*, *HIST1H2BJ*, *IL1B*, *DLG3*, *PDGFC*, *PDGFD*, *PTX3*, *ADAM9*, *ICAM1*, *ANXA1*, *SPARC*, *F5*, *PPBP*, *ADM*, *COLQ*, *HIST2H2BE*, *FRMD4B*, *IGLC2*, *CD14*, and *CMTM5*
Platelet alpha granule lumen	7	6.55E-06	*VWF*, *APP*, *F5*, *PPBP*, *CLU*, *PF4*, and *SPARC*
Cell surface	17	1.34E-05	*ICAM1*, *TNF*, *LDLR*, *CLU*, *ANXA1*, *ITGB5*, *SPARC*, and *ITGB3*, *APP*, *SDC1*, *GP6*, *GP1BA*, *PDGFC*, *TREML1*, *INTU*, *ITGA2B*, and *ADAM9*
Extracellular exosome	44	4.28E-05	*HIST1H2AC*, *NAMPT*, *CLU*, *FAM20C*, *CLDN5*, *JCHAIN*, *FASLG*, *ITGB5*, *ANPEP*, *ITGB3*, *ATP2B1*, *PRKAR2B*, *CTTN*, *APP*, *IGLV3-19*, *GP6*, *PGRMC1*, *IL1B*, *PDGFC*, *ESAM*, *GP1BA*, *FAM129B*, *PDGFD*, *TUBA1A*, *TUBB1*, *ADAM9*, *ICAM1*, *ANXA1*, *WLS*, *MYADM*, *PLAUR*, *IGLV2-11*, *PDZK1IP1*, *VWF*, *IGKV1-5*, *SDC1*, *HIST2H2BE*, *SH3BGRL2*, *CPNE2*, *IGLC2*, *CD14*, *XYLB*, *ALOX12*, and *ITGA2B*
Membrane raft	9	3.55E-04	*ATP2B1*, *PRKAR2B*, *ICAM1*, *APP*, *TNF*, *SDPR*, *KCNQ1*, *MYADM*, and *CD14*
External side of the plasma membrane	9	4.43E-04	*ICAM1*, *SDC1*, *TNF*, *LDLR*, *FASLG*, *ANPEP*, *IGLC2*, *KLRD1*, and *ITGA2B*
Focal adhesion	11	0.002	*ICAM1*, *SDC1*, *CTTN*, *ZNF185*, *ANXA1*, *ITGB5*, *TSPAN9*, *ITGB3*, *ADAM9*, *PLAUR*, and *ITGA2B*
Blood microparticle	7	0.002	*IGKV1-5*, *CLU*, *JCHAIN*, *IGKV4-1*, *IGLC2*, *IGKV3-15*, and *ITGA2B*
Platelet alpha granule membrane	3	0.005	*SPARC*, *ITGB3*, and *ITGA2B*
Platelet alpha granule	3	0.006	*VWF*, *SPARC*, and *TREML1*
Integral component of the plasma membrane	22	0.008	*ICAM1*, *TNF*, *LDLR*, *SLCO4A1*, *FASLG*, *ANPEP*, *TSPAN9*, *ITGB3*, *NCR1*, *AQP10*, *PLAUR*, *GP9*, *ATP2B1*, *APP*, *SDC1*, *GP6*, *FCAR*, *GP1BA*, *KLRF1*, *KIR2DL3*, *CLEC1B*, and *ITGA2B*
Phagocytic cup	3	0.011	*TNF*, *PEAR1*, and *ANXA1*
Clathrin-coated pit	4	0.011	*APP*, *CTTN*, *LDLR*, and *LRP3*
Anchored component of the external side of the plasma membrane	3	0.014	*GGTA1P*, *GP1BA*, and *CD14*
Basolateral plasma membrane	6	0.018	*ATP2B1*, *LDLR*, *ANXA1*, *DLG3*, *KCNQ1*, and *ADAM9*
Integrin complex	3	0.021	*ITGB5*, *ITGB3*, and *ITGA2B*
Receptor complex	5	0.022	*APP*, *LDLR*, *ITGB5*, *ITGB3*, and *KLRD1*
Dendritic shaft	3	0.029	*PRKAR2B*, *APP*, and *DLG3*
Cytoplasm	55	0.043	*NAMPT*, *MCM10*, *ISG20*, *PRKAR2B*, *CTTN*, *APP*, *HOMER3*, *SDPR*, *ZNF185*, *HIST1H2BJ*, *DLG3*, *PIWIL2*, *PDGFC*, *FAM129B*, *TUBB1*, *KCNQ1*, *EGR1*, *SGK1*, *EGR2*, *PADI4*, *UBE2C*, *NLRP3*, *AHR*, *SH2D2A*, *SDC1*, *ADM*, *HIST2H2BE*, *FRMD4B*, *PPP1R15A*, *INTU*, *XYLB*, *ALOX12*, *MCL1*, *ABLIM3*, *CLU*, *TRIB1*, *SPATS2*, *NCAPG*, *RNF165*, *STRIP2*, *SKA3*, *GP1BA*, *HRASLS2*, *ANXA1*, *LMNA*, *CDC20*, *SPARC*, *RAPH1*, *SH3BGRL2*, *GAS2L1*, *CPNE2*, *RFX2*, *TREML1*, *KLF4*, and *DUSP6*
Molecular function			
Antigen binding	12	1.30E-09	*IGLV2-11*, *IGLV2-8*, *IGLV3-19*, *IGKV1-5*, *IGLV1-44*, *IGLV2-23*, *IGKV5-2*, *JCHAIN*, *IGKV4-1*, *KIR2DL3*, *IGLC2*, and *IGKV3-15*
Receptor activity	11	1.82E-05	*ICAM1*, *IL18R1*, *GP6*, *IL18RAP*, *LDLR*, *ITGB5*, *ANPEP*, *TREM1*, *ITGB3*, *KIR2DL3*, and *PLAUR*
Serine-type endopeptidase activity	11	7.17E-05	*IGLV2-11*, *IGLV2-8*, *IGLV3-19*, *IGKV1-5*, *F5*, *IGLV1-44*, *IGLV2-23*, *IGKV5-2*, *IGKV4-1*, *IGLC2*, and *IGKV3-15*
Virus receptor activity	5	0.003	*ICAM1*, *LDLR*, *ITGB5*, *ANPEP*, and *ITGB3*
Platelet-derived GF receptor binding	3	0.007	*PDGFC*, *PDGFD*, and *ITGB3*
Collagen binding	4	0.015	*VWF*, *GP6*, *SPARC*, and *ADAM9*
ECM binding	3	0.021	*SPARC*, *ITGB3*, and *ITGA2B*
IgA binding	2	0.026	*FCAR* and *JCHAIN*
Pathway			
KEGG pathway			
ECM‒receptor interaction	8	4.38E-05	*VWF*, *SDC1*, *GP6*, *ITGB5*, *GP1BA*, *ITGB3*, *GP9*, and *ITGA2B*
Hematopoietic cell lineage	8	4.38E-05	*TNF*, *IL1B*, *ANPEP*, *GP1BA*, *ITGB3*, *CD14*, *GP9*, and *ITGA2B*
African trypanosomiasis	4	0.006	*ICAM1*, *TNF*, *IL1B*, and *FASLG*
Pertussis	5	0.009	*TNF*, *CXCL5*, *IL1B*, *NLRP3*, and *CD14*
Hypertrophic cardiomyopathy	5	0.011	*TNF*, *LMNA*, *ITGB5*, *ITGB3*, and *ITGA2B*
NK cell‒mediated cytotoxicity	6	0.011	*ICAM1*, *TNF*, *FASLG*, *KIR2DL3*, *NCR1*, and *KLRD1*
PI3K‒Akt signaling pathway	10	0.013	*VWF*, *SGK1*, *MCL1*, *ITGB5*, *FASLG*, *GNG11*, *PDGFC*, *PDGFD*, *ITGB3*, and *ITGA2B*
Dilated cardiomyopathy	5	0.014	*TNF*, *LMNA*, *ITGB5*, *ITGB3*, and *ITGA2B*
Platelet activation	6	0.014	*VWF*, *GP6*, *GP1BA*, *ITGB3*, *GP9*, and *ITGA2B*
Malaria	4	0.017	*ICAM1*, *SDC1*, *TNF*, and *IL1B*
Cytokine‒cytokine receptor interaction	8	0.017	*IL18R1*, *TNF*, *IL18RAP*, *PPBP*, *CXCL5*, *IL1B*, *FASLG*, and *PF4*
Pathogenic *E. coli* infection	4	0.018	*CTTN*, *TUBB1*, *TUBA1A*, and *CD14*
Proteoglycans in cancer	7	0.023	*SDC1*, *CTTN*, *TNF*, *ITGB5*, *FASLG*, *ITGB3*, and *PLAUR*
Phagosome	6	0.025	*FCAR*, *ITGB5*, *ITGB3*, *TUBB1*, *TUBA1A*, and *CD14*
Inflammatory bowel disease	4	0.033	*IL18R1*, *TNF*, *IL18RAP*, and *IL1B*
Arrhythmogenic right ventricular cardiomyopathy	4	0.037	*LMNA*, *ITGB5*, *ITGB3*, and *ITGA2B*
Reactome pathway			
Immunoregulatory interactions between a Lymphoid and a non-Lymphoid cell	17	2.07E-10	*ICAM1*, *IGLV1-44*, *IGKV5-2*, *NCR1*, *IGLV2-11*, *IGLV2-8*, *IGLV3-19*, *IGKV1-5*, *IGLV2-23*, *IGKV4-1*, *KLRF1*, *TREM1*, *KIR2DL3*, *TREML1*, *IGLC2*, *KLRD1*, and *IGKV3-15*
Scavenging of heme from plasma	11	4.03E-09	*IGLV2-11*, *IGLV2-8*, *IGLV3-19*, *IGKV1-5*, *IGLV1-44*, *IGLV2-23*, *IGKV5-2*, *JCHAIN*, *IGKV4-1*, *IGLC2*, and *IGKV3-15*
CD22-mediated B-cell receptor regulation	10	2.14E-08	*IGLV2-11*, *IGLV2-8*, *IGLV3-19*, *IGKV1-5*, *IGLV1-44*, *IGLV2-23*, *IGKV5-2*, *IGKV4-1*, *IGLC2*, and *IGKV3-15*
FCERI signaling	10	2.14E-08	*IGLV2-11*, *IGLV2-8*, *IGLV3-19*, *IGKV1-5*, *IGLV1-44*, *IGLV2-23*, *IGKV5-2*, *IGKV4-1*, *IGLC2*, and *IGKV3-15*
Classical antibody-mediated complement activation	10	3.31E-08	*IGLV2-11*, *IGLV2-8*, *IGLV3-19*, *IGKV1-5*, *IGLV1-44*, *IGLV2-23*, *IGKV5-2*, *IGKV4-1*, *IGLC2*, and *IGKV3-15*
Role of LAT2/NTAL/LAB on calcium mobilization	10	8.38E-08	*IGLV2-11*, *IGLV2-8*, *IGLV3-19*, *IGKV1-5*, *IGLV1-44*, *IGLV2-23*, *IGKV5-2*, *IGKV4-1*, *IGLC2*, and *IGKV3-15*
FCGR activation	10	7.38E-08	*IGLV2-11*, *IGLV2-8*, *IGLV3-19*, *IGKV1-5*, *IGLV1-44*, *IGLV2-23*, *IGKV5-2*, *IGKV4-1*, *IGLC2*, and *IGKV3-15*
Initial triggering of complement	10	1.36E-07	*IGLV2-11*, *IGLV2-8*, *IGLV3-19*, *IGKV1-5*, *IGLV1-44*, *IGLV2-23*, *IGKV5-2*, *IGKV4-1*, *IGLC2*, and *IGKV3-15*
Role of phospholipids in phagocytosis	10	3.31E-07	*IGLV2-11*, *IGLV2-8*, *IGLV3-19*, *IGKV1-5*, *IGLV1-44*, *IGLV2-23*, *IGKV5-2*, *IGKV4-1*, *IGLC2*, and *IGKV3-15*
FCERI-mediated Ca^2+^ mobilization	10	5.49E-07	*IGLV2-11*, *IGLV2-8*, *IGLV3-19*, *IGKV1-5*, *IGLV1-44*, *IGLV2-23*, *IGKV5-2*, *IGKV4-1*, *IGLC2*, and *IGKV3-15*
FCERI-mediated MAPK activation	10	4.97E-07	*IGLV2-11*, *IGLV2-8*, *IGLV3-19*, *IGKV1-5*, *IGLV1-44*, *IGLV2-23*, *IGKV5-2*, *IGKV4-1*, *IGLC2*, and *IGKV3-15*
Antigen activates B-cell receptor leading to the generation of second messengers	10	8.82E-07	*IGLV2-11*, *IGLV2-8*, *IGLV3-19*, *IGKV1-5*, *IGLV1-44*, *IGLV2-23*, *IGKV5-2*, *IGKV4-1*, *IGLC2*, and *IGKV3-15*
Regulation of actin dynamics for phagocytic cup formation	10	6.50E-06	*IGLV2-11*, *IGLV2-8*, *IGLV3-19*, *IGKV1-5*, *IGLV1-44*, *IGLV2-23*, *IGKV5-2*, *IGKV4-1*, *IGLC2*, and *IGKV3-15*
CERI-mediated NF-kB activation	10	2.18E-05	*IGLV2-11*, *IGLV2-8*, *IGLV3-19*, *IGKV1-5*, *IGLV1-44*, *IGLV2-23*, *IGKV5-2*, *IGKV4-1*, *IGLC2*, and *IGKV3-15*
Platelet degranulation	9	1.01E-04	*VWF*, *APP*, *F5*, *PPBP*, *CLU*, *PF4*, *SPARC*, *ITGB3*, and *ITGA2B*
Platelet adhesion to exposed collagen	4	5.69E-04	*VWF*, *GP6*, *GP1BA*, and *GP9*
GP1b-IX-V activation signaling	3	0.005	*VWF*, *GP1BA*, and *GP9*
Mitotic prometaphase	6	0.007	*SPC24*, *KIF2C*, *CDC20*, *AURKB*, *TUBB1*, and *TUBA1A*
ECM proteoglycans	5	0.01	*APP*, *ITGB5*, *SPARC*, *ITGB3*, and *ITGA2B*
GRB2:SOS provides linkage to MAPK signaling for Integrins	3	0.012	*SPC24*, *KIF2C*, *CDC20*, *AURKB*, *TUBB1*, and *TUBA1A*
p130Cas linkage to MAPK signaling for integrins	3	0.012	*VWF*, *ITGB3*, and *ITGA2B*
Resolution of Sister Chromatid Cohesion	6	0.012	*VWF*, *ITGB3*, and *ITGA2B*
Integrin cell surface interactions	5	0.016	*ICAM1*, *VWF*, *ITGB5*, *ITGB3*, and *ITGA2B*
RHO GTPases Activate Formins	6	0.018	*SPC24*, *KIF2C*, *CDC20*, *AURKB*, *UBE2C*, *TUBB1*, and *TUBA1A*
Separation of Sister Chromatids	7	0.018	*SPC24*, *KIF2C*, *CDC20*, *AURKB*, *TUBB1*, and *TUBA1A*
Cell surface interactions at the vascular wall	4	0.023	*GP6*, *ESAM*, *PF4*, and *TREM1*
Intrinsic pathway of fibrin clot formation	3	0.025	*PRKAR2B*, *TUBB1*, *TUBA1A*, and *NTU*
Hedgehog 'off' state	4	0.025	*VWF*, *GP1BA*, and *GP9*
Integrin alpha IIb beta 3 signaling	3	0.027	*VWF*, *ITGB3*, and *ITGA2B*
Syndecan interactions	3	0.037	*SDC1*, *ITGB5*, and *ITGB3*

DEGs with a significant change between children with AD and healthy control children (cutoff FC ≥1.5 and FDR <0.05) were used for GO enrichment and pathway analyses using DAVID database. KEGG and Reactome pathway analyses were used to determine the pathways of DEGs between two groups.

Abbreviations: AD, atopic dermatitis; Akt, protein kinase B; Ca^2+^, calcium ion; DEG, differentially expressed gene; ECM, extracellular matrix; FC, fold change; FCERI, Fc-epsilon receptor; FDR, false discovery rate; GO, gene ontology; KEGG, Kyoto Encyclopedia of Genes and Genomes; MMP, matrix metalloproteinase; PI3K, phosphoinositide 3-kinase.

### Construction of protein‒protein interaction network and module analysis

**Figure 5:**
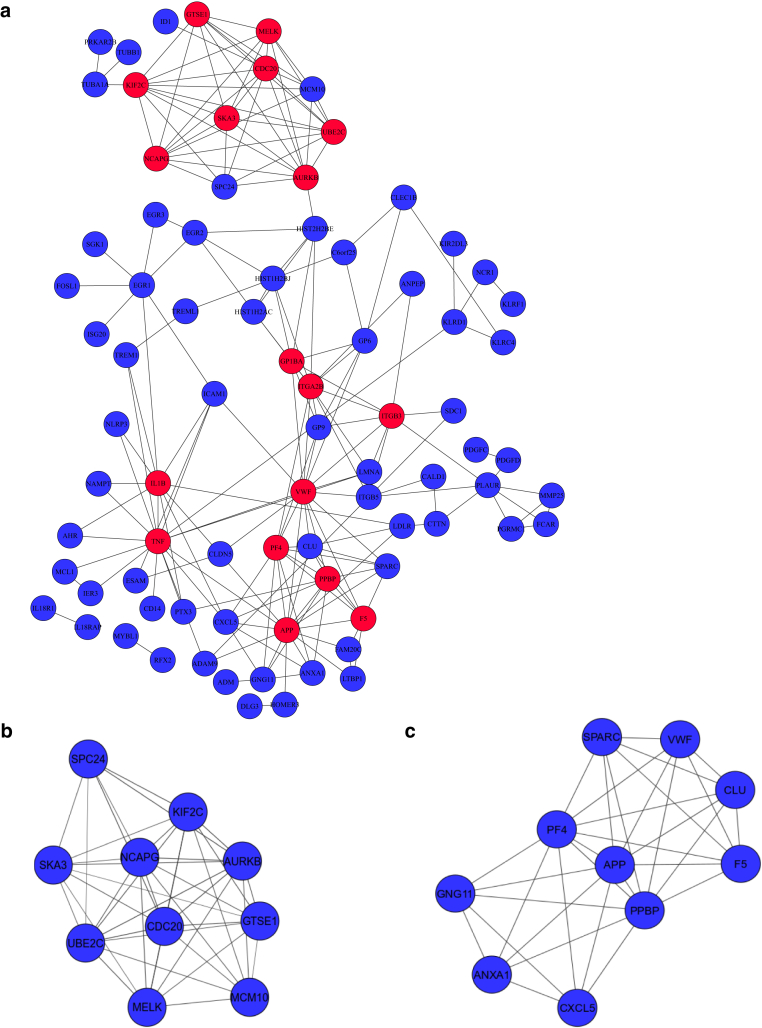
**PPI networks.** (**a**) PPI network with 82 nodes. In the network, nodes represent proteins, and lines (edges) represent the interactions between proteins. Red nodes represent the hub nodes with a large number of neighbors (≥8). (**b**) A first significant module with 12 nodes identified by MCODE. (**c**) A second significant module with 12 nodes identified by MCODE. MCODE, Molecular Complex Detection; PPI, protein‒protein interaction.

To explore interactions among the DEG genes, STRING (Search Tool for the Retrieval of Interacting Genes/Proteins) analysis was applied, and the most important modules were then screened and visualized using Cytoscape software. A protein‒protein interaction (PPI) network containing 82 connected nodes (proteins) and 194 interaction edges (interactions of proteins), where the average degree of connectivity (i.e., the average number of neighbors) was 4.732, is presented in Figure 5a. The hub nodes with the greatest number of neighbors (≥8), such as TNF, IL-1β, Von Willebrand factor, and ITGB3, were identified (labeled in red in Figure 5a) and analyzed by GO enrichment and pathway analyses (Table 6). The Kyoto Encyclopedia of Genes and Genomes pathway analysis revealed that the hub genes were involved in the cytokine‒cytokine receptor interaction, hematopoietic cell lineage, extracellular matrix‒receptor interaction, platelet activation, cell division, and other pathways. In addition, two significant modules with 10 nodes were obtained from the PPI network of DEGs using Molecular Complex Detection (Figure 5b and c). Enrichment analysis suggested that the genes in the first significant module (Figure 5b) were mainly associated with functional terms in the category BP, including cell division, cell proliferation, and mitotic nuclear division. In the category CC, the genes in this module were significantly enriched in cytosol and nucleus, and in the category MF, the genes were mainly enriched in protein and adenosine triphosphate binding. The genes in the second module (Figure 5c) were significantly enriched in inflammatory response, chemokine-mediated signaling, platelet degranulation and activation, immune response, and signal transduction in the category BP. In the category CC, the genes were significantly enriched in the extracellular region, extracellular space, and platelet alpha granule lumen, and in the category MF, the genes were mainly enriched in chemokine activity and CXCR chemokine receptor binding. Furthermore, results from the Kyoto Encyclopedia of Genes and Genomes analysis showed that the genes in this significant module were associated with chemokine signaling pathway and cytokine‒cytokine receptor interaction (Table 7).

**Table 6:** GO Enrichment and Pathway Analyses of Hub Genes

GO Term	Gene Count	*P*-Value
Biological process		
Platelet degranulation	7	5.39E-10
Platelet activation	5	4.64E-06
Extracellular matrix organization	5	3.80E-05
Cell division	5	3.56E-04
Negative regulation of extrinsic apoptotic signaling pathway in absence of ligand	3	6.29E-04
Platelet aggregation	3	7.73E-04
Blood coagulation	4	7.86E-04
Cell adhesion	5	9.88E-04
Mitotic nuclear division	4	0.002
Anaphase-promoting complex-dependent catabolic process	3	0.003
Positive regulation of calcidiol 1-monooxygenase activity	2	0.003
Sequestering of triglyceride	2	0.004
Sister chromatid cohesion	3	0.005
Positive regulation of fever generation	2	0.005
Inflammatory response	4	0.006
Regulation of establishment of endothelial barrier	2	0.007
Positive regulation of protein phosphorylation	3	0.007
Cytokine-mediated signaling pathway	3	0.008
Regulation of chromosome segregation	2	0.008
Negative regulation of lipid storage	2	0.008
Immune response	4	0.008
Positive regulation of ubiquitin-protein ligase activity	2	0.009
Positive regulation of chemokine biosynthetic process	2	0.010
Protein ubiquitination involved in ubiquitin-dependent protein catabolic process	3	0.010
Positive regulation of heterotypic cell-cell adhesion	2	0.011
Regulation of cell proliferation	3	0.015
Regulation of I-kB kinase/NF-kB signaling	2	0.015
Positive regulation of membrane protein ectodomain proteolysis	2	0.015
Positive regulation of VEGF receptor signaling pathway	2	0.016
Negative regulation of lipid catabolic process	2	0.016
Cell‒substrate adhesion	2	0.017
Proteasome-mediated ubiquitin-dependent protein catabolic process	3	0.018
Blood coagulation, intrinsic pathway	2	0.018
Positive regulation of leukocyte chemotaxis	2	0.018
Positive regulation of protein export from the nucleus	2	0.019
Positive regulation of NF-kB import into the nucleus	2	0.021
Regulation of ubiquitin-protein ligase activity involved in mitotic cell cycle	2	0.023
Positive regulation of IL-8 production	2	0.026
Positive regulation of gene expression	3	0.028
Positive regulation of phagocytosis	2	0.029
Lipopolysaccharide-mediated signaling pathway	2	0.032
Protein kinase B signaling	2	0.033
Positive regulation of nitric oxide biosynthetic process	2	0.043
Positive regulation of IL-6 production	2	0.045
Positive regulation of IFN-γ production	2	0.046
Positive regulation of cell division	2	0.047
Cellular component		
Platelet alpha granule lumen	5	1.72E-07
Cell surface	5	0.001
Extracellular region	7	0.002
Kinetochore	3	0.003
Extracellular space	6	0.006
Membrane	7	0.012
Platelet alpha granule membrane	2	0.012
Spindle mid-zone	2	0.018
Anaphase-promoting complex	2	0.021
Cytosol	8	0.023
ER to Golgi transport vesicle	2	0.024
Integrin complex	2	0.025
Integral component of plasma membrane	5	0.038
Spindle microtubule	2	0.040
Cytoplasmic microtubule	2	0.047
Molecular function		
Protein binding	16	0.002
Protease binding	3	0.005
Identical protein binding	5	0.006
Extracellular matrix binding	2	0.026
Enzyme binding	3	0.043
Chemokine activity	2	0.048
KEGG pathway		
Hematopoietic cell lineage	5	1.09E-05
ECM‒receptor interaction	4	3.96E-04
Platelet activation	4	0.001
Cytokine‒cytokine receptor interaction	4	0.008
Hypertrophic cardiomyopathy	3	0.008
Dilated cardiomyopathy	3	0.009
Osteoclast differentiation	3	0.021
Alzheimer's disease	3	0.033
Focal adhesion	3	0.048

Hub genes identified in PPI network were used for GO enrichment and pathway analyses using DAVID database.

Abbreviations: ECM, extracellular matrix; ER, endoplasmic reticulum; GO, gene ontology; KEGG, Kyoto Encyclopedia of Genes and Genomes; PPI, protein‒protein interaction.

**Table 7:** GO Enrichment and Pathway Analyses of Significant Modules

GO Term	Gene Count	*P*-Value
Module 1		
Biological process		
Cell division	6	4.50E-07
Mitotic nuclear division	5	5.52E-06
Sister chromatid cohesion	4	1.83E-05
Anaphase-promoting complex-dependent catabolic process	3	7.70E-04
Cell proliferation	4	7.82E-04
Protein ubiquitination involved in ubiquitin-dependent protein Catabolic process	3	0.003
Regulation of chromosome segregation	2	0.004
Positive regulation of ubiquitin-protein ligase activity	2	0.005
Proteasome-mediated ubiquitin-dependent protein catabolic process	3	0.005
Regulation of ubiquitin-protein ligase activity involved in mitotic cell cycle	2	0.012
Negative regulation of ubiquitin-protein ligase activity involved in mitotic cell cycle	2	0.037
Positive regulation of ubiquitin-protein ligase activity involved in regulation of mitotic cell cycle transition	2	0.040
Cellular component		
Kinetochore	3	6.88E-04
Cytosol	7	0.002
Anaphase-promoting complex	2	0.011
Spindle microtubule	2	0.022
Nucleus	7	0.024
Cytoplasmic microtubule	2	0.025
Nucleoplasm	5	0.036
Condensed chromosome kinetochore	2	0.042
Molecular Function		
Protein binding	10	0.003
ATP binding	4	0.039
KEGG pathway		
Ubiquitin mediated proteolysis	2	0.039
Module 2		
Biological process		
Platelet degranulation	7	3.80E-12
Positive regulation of leukocyte chemotaxis	3	3.89E-05
Response to lipopolysaccharide	4	7.36E-05
Chemokine-mediated signaling pathway	3	6.23E-04
Inflammatory response	4	8.66E-04
Platelet activation	3	0.002
Regulation of cell proliferation	3	0.004
Extracellular matrix organization	3	0.005
Response to lead ion	2	0.007
G-protein coupled receptor signaling pathway	4	0.010
Immune response	3	0.020
Signal transduction	4	0.020
Innate immune response	3	0.021
Response to peptide hormone	2	0.023
Positive regulation of TNF production	2	0.025
Negative regulation of angiogenesis	2	0.033
Cellular component		
Platelet alpha granule lumen	7	4.75E-14
Extracellular region	9	3.03E-08
Extracellular space	8	3.74E-07
cell surface	4	0.002
platelet alpha granule	2	0.007
ER to Golgi transport vesicle	2	0.013
mitochondrial membrane	2	0.045
Molecular function		
chemokine activity	3	2.93E-04
CXCR chemokine receptor binding	2	0.005
collagen binding	2	0.032
chaperone binding	2	0.042
KEGG pathway		
Chemokine signaling pathway	4	3.66E-04
Cytokine‒cytokine receptor interaction	3	0.017

Two significant modules identified in PPI network were used for GO enrichment and pathway analyses using DAVID database.

Abbreviations: ATP, adenosine triphosphate; ER, endoplasmic reticulum; GO, gene ontology; KEGG, Kyoto Encyclopedia of Genes and Genomes; PPI, protein‒protein interaction.

## Discussion

AD is a complex disease associated with immunological and epidermal barrier dysfunctions. Most of our knowledge in the field of AD is based on studies performed in adult patients with AD, although remarkable differences between pediatric and adult AD have been shown recently. Therefore, it is of great importance to identify the molecular basis of pediatric AD and elucidate the biomarkers that could help to identify young patients at risk at an earlier stage of life and to explore new therapies in pediatric AD. Given that more than half of all cases of AD begin during the first year of life, we aimed to discover signature biomarkers of AD in infants. Considering that skin biopsies are very difficult to obtain at such a young age, that AD generates a systemic immunological response, and that blood is a noninvasive source of biological tissue, we analyzed blood profiles of pediatric patients with AD in the first year of life.

Using RNA-seq transcriptome profile of peripheral blood cells obtained from infants with AD or healthy infants, we identified 178 genes differentially expressed in pediatric patients with AD: 115 were upregulated, and 63 were downregulated. To further investigate the functions of the DEGs, GO functional annotation and pathway enrichment analysis were used on the basis of the DAVID database. The GO analysis showed that DEGs were associated with immune responses, inflammatory responses, regulation of immune responses, and platelet activation, which are all known to be AD related. The results of the pathway analysis indicated that the DEGs were enriched in immunoregulatory interactions between a lymphoid and a nonlymphoid cell, hematopoietic cell lineage, phosphoinositide 3-kinase‒protein kinase B signaling pathway, cytokine‒cytokine receptor interaction, NK cell‒mediated cytotoxicity, and platelet activation.

Randomly selected DEGs were further validated in a larger number of samples collected from patients with AD in the first year of life using RT-qPCR. Among highly upregulated genes, we identified *IL1β*, previously shown to be upregulated in the serum of adult AD (Thijs et al., 2018) and stratum corneum of our pediatric AD collection as reported previously (McAleer et al., 2019). It has been shown to be involved in AD development (Bernard et al., 2017). IL-1β is a potent proinflammatory cytokine that can mediate inflammatory responses by supporting T-cell survival, upregulation of the IL-2 receptor on lymphocytes, enhancing antibody production of B cells, and promoting B-cell proliferation and T helper 17 cell differentiation (Lamkanfi et al., 2011). IL-1β activity is regulated at multiple levels, one of which is controlled by inflammasomes (Schroder and Tschopp, 2010). Recent findings suggest that inflammasome-dependent IL-1β activation plays a role in a variety of disorders, including AD. Of note, among upregulated DEGs, we identified *NLRP3*, one of the important inflammasome proteins.

Another interesting upregulated DEG in our pediatric patients was *TREM1*. Recently, it has been reported to be highly expressed in lesional skin and serum of adult AD (Suarez-Farinas et al., 2015). It has also been reported to be expressed in psoriasis and has been suggested to be a therapeutic target to modify the effects of inflammatory myeloid dendritic cells in psoriasis (Hyder et al., 2013). TREM1 (CD354) is a cell surface receptor that is expressed on various types of cells: monocytes, neutrophils NK cells, dendritic cells, and B and T cells and has been implicated in innate and adaptive immune responses. Activation of TREM1 was shown to result in the production of a variety of inflammatory cytokines, including TNF, IL6, MCP1, and IL-1β, and amplification of toll-like receptor‒initiated inflammation (Roe et al., 2014). Of interest, TNF was highly expressed in the blood cells of our pediatric AD collection. It has been shown to be involved in inflammatory processes in AD (Jacobi et al., 2005; Sumimoto et al., 1992). Furthermore, TNF together with the T helper 2 cytokines induced AD-like features in a skin model (Danso et al., 2014). In addition, TNF together with TNF-like weak inducer of apoptosis induced keratinocyte apoptosis in AD skin (Zimmermann et al., 2011).

Another interesting group of genes found to be upregulated in the blood of pediatric patients with AD included early growth response genes (*EGR1*, *EGR2*, and *EGR3*), a family of zinc-finger transcription factors. *EGR1*, an important player in the regulation of cell growth, differentiation, cell survival, and immune responses, has been reported to be upregulated in psoriatic skin lesions (Jeong et al., 2014). EGR2/3 is known to play a crucial role in the regulation of the immune system. They control inflammation, regulate B- and T-cell function in adaptive immune responses, and have been suggested to be involved in preventing the development of autoimmune disease (Morita et al., 2016) and limiting immunopathology during productive adaptive immune responses (Li et al., 2012). Notably, EGR2 is located in a susceptibility locus for AD identified by GWAS in the Japanese population (Hirota et al., 2012).

Among the downregulated genes, we identified *IL18R1* and *IL18RAP*, also previously found to be associated with AD (Hirota et al., 2012). *IL18RAP* enhances the *IL18*-binding activity of the IL18 receptor (*IL18R1*) and plays a role in signaling by IL-18, a pleiotropic immune regulator. IL-18 plays a strong proinflammatory role by inducing IFN-γ. Previous studies have implicated IL-18 in the pathogenesis of AD. It has been shown to contribute to the spontaneous development of AD-like skin lesions in a transgenic mouse model (Konishi et al., 2002). It has been reported to be elevated in skin lesions of adults with AD. In our previous study, we analyzed plasma and stratum corneum biomarkers in this collection of patients and showed that IL-18 was observed in very high levels in the stratum corneum of pediatric patients; however, no difference was observed in IL-18 plasma levels (McAleer et al., 2019). Another study showed that PBMCs from patients with AD have a decreased IL-18 expression and capacity to produce IFN-γ, which is inversely correlated with serum IgE concentrations (Higashi et al., 2001), suggesting an IL-18 role in the skewing of the immune system in patients with AD.

Another gene significantly downregulated in AD infants was *GLDC*. *GLDC*, glycine metabolism and the metabolic enzyme glycine decarboxylase, is a key enzyme of the mitochondrial glycine cleavage system (Hiraga and Kikuchi, 1980). GLDC plays important role in many human cancers (Zhang et al., 2012). It has been shown to be differentially expressed in psoriatic skin (Rittie et al., 2016). Interestingly, GLDC has been reported to be differentially expressed in AD-like‒reconstructed human epidermis (Evrard et al., 2021), suggesting its involvement in AD development.

A PPI network among the screened DEGs was predicted. The PPI analysis allowed us to determine significant modules and hub genes. In the resulting PPI network, 18 hub genes with the highest degree of connectivity were selected, which included *IL1β*, Von Willebrand factor gene *VWF*, *PF4*, *ITGB3*, *ITGA2B*, *APP*, *F5*, *AURKB*, *SKA3*, *MELK*, *CDC20*, *PPBP*, *NCAPG*, *GTSE1*, *KIF2C*, *GP1BA*, *UBE2C*, and *TNF*. Pathway analysis revealed that the hub genes were involved in the cytokine‒cytokine receptor interaction, hematopoietic cell lineage, extracellular matrix‒receptor interaction, cell division, platelet activation, and other pathways. In addition, two significant modules were identified. The genes in the first significant module were mainly associated with cell division, cell proliferation, and mitotic nuclear division. The genes in the second module were significantly enriched in inflammatory response, chemokine-mediated signaling, platelet degranulation and activation, immune response, and signal transduction and were associated with chemokine signaling pathway and cytokine‒cytokine receptor interaction.

We wondered whether the hub genes could be linked to AD or other skin inflammatory diseases. The important role of IL-1β and TNF in AD has been shown earlier in this report. Von Willebrand factor, a key player in hemostasis, has been reported in relation to cutaneous inflammation (Hillgruber et al., 2014). Increased expression of PF4 has been proposed to play an important role in the etiology of AD (Watanabe et al., 1999). Increased ITGB3 expression has been reported in T helper 17‒associated skin inflammatory diseases such as psoriasis (Goedkoop et al., 2004) and psoriatic arthritis (Canete et al., 2004). A significant increase in platelet-leukocyte aggregates expressing ITGA2B was found in the blood of mice with chronic hapten-induced allergic dermatitis (Tamagawa-Mineoka et al., 2007). PPBP has been found to be important for regulating excessive inflammation in psoriasis (Oka et al., 2017). Elevated AURKB expression in lesional psoriatic tissues has been suggested to contribute to the development of psoriasis (Liu et al., 2011). Actively proliferating UBE2C+TOP2A+ type 2/type 22 T cells were expanded in lesional AD skin and were either absent or less abundant in nonlesional and healthy samples (He et al., 2020). These findings suggest that identified hub genes could be considered important candidates for prognostic and therapeutic targets of pediatric AD.

Taken together, this study showed that blood gene expression profile identified distinct key genes and pathways of early-onset pediatric AD. Observed dramatic changes in the PBMC transcriptome were predominantly related to immune responses in AD. New data assessed from this study may help to better understand the processes leading to AD in infants and may serve in the development of novel treatment possibilities. However, to decipher the full mechanism involved in pediatric AD pathogenesis, skin RNA profile should be further investigated in infants with AD. Blood profile along with skin profile in infants with AD could provide us with a larger number of potential biomarkers that may contribute to AD prediction, risk of comorbidity development, and responses to AD treatment in infants.

## Materials and Methods

### Patients

We recruited infants aged <12 months with moderate-to-severe AD who were treatment naive (apart from the use of emollients and 1% hydrocortisone cream or ointment) along with age-matched healthy controls. The study was approved by the Research Ethics Committee of Children's Health Ireland at Crumlin (Dublin, Ireland) and was conducted in compliance with the Helsinki Declaration. Written informed consent was given by parents or legal guardians for all study subjects. The age of onset of AD was recorded. Severity was assessed by the SCORing Atopic Dermatitis index. Clinical and demographic features are summarized in Table 8. Analysis of cytokine and microRNA biomarkers in this collection has previously been reported (McAleer et al., 2019; Nousbeck et al., 2021).

**Table 8:** Clinical and Demographical Characteristics of the Study Participants

Characteristics	Patients with AD	Healthy Controls
Total	27	17
Sex		
Male	18	11
Female	9	6
Age (mo)		
Average	6.9	7.94
Range	3-10	3-12
Age of AD onset (wk)		
Average	9	—
Range	4‒20	—
SCORAD		
Average	49.4	—
Range	23.4‒91.3	—

Abbreviations: AD, atopic dermatitis; SCORAD, SCORing Atopic Dermatitis.

### PBMC preparation and RNA isolation

PBMCs were isolated from whole blood as previously described (Nousbeck et al., 2021) using histopaque double-gradient density centrifugation (Sigma-Aldrich, St. Louis, MO) and cryopreserved for further analysis. Total RNA was isolated from PBMCs according to RNeasy Mini Kit protocol (Qiagen, Hilden, Germany). RNA concentrations, RNA integrity, and quality of RNA were evaluated using Qubit fluorometer (Thermo Fisher Scientific, Waltham, MA) and RNA 6000 Nano Lab Chips on an Agilent 2100 Bioanalyzer (Agilent Technologies, Santa Clara, CA). RNA samples with optimal RNA integrity number values (≥8) were considered to construct libraries for sequencing.

### RNA-seq, data processing, and differential expression analysis

Library preparation (using Illumina TruSeq stranded mRNAseq library kit) and sequencing were conducted by Edinburgh Genomics, The University of Edinburgh (Edinburgh, United Kingdom). The sequencing of the libraries was performed with Illumina NovaSeq 6000 (100 cycles, 50 base pair paired-end sequencing). Sequencing reads showed excellent quality, with the overall Q30 above 94%. After sequencing, reads were trimmed using Cutadapt (Martin, 2011), and clean paired-end reads were mapped to the human reference genome GRCh38 using STAR software (Dobin et al., 2013). The number of reads for each gene was counted using featureCounts (Liao et al., 2014), and the count matrix was used for differential expression analysis. Differential expression was performed using package DESeq2 in R software (version 3.5.2), considering an expression >20 read counts in at least 25% of the samples, a cutoff of at least 1.5-fold change in expression, and a Benjamini‒Hochberg‒corrected false discovery rate < 0.05.

### Real-time RT-qPCR

The DEGs were further verified using RT-qPCR. Briefly, total RNA was reverse transcribed using SensiFAST cDNA synthesis kit (Bioline, London, United Kingdom). cDNA PCR amplification was carried out using the SensiFAST SYBR Hi-ROX Kit (Bioline) on 7900HT Fast Real-Time PCR System with gene-specific intron-crossing oligonucleotide pairs. Primers are available in Table 9. Results were normalized to *GAPDH* mRNA levels. Triplicates of each reaction were performed as the mean ± SD. Relative quantification of target mRNA expression was performed using the 2-ΔΔCT method (Livak and Schmittgen, 2001). The normalized expression data were log_2_ transformed before data analysis.

**Table 9:** Primers Sequences for RT-qPCR

Gene	Forward Primer	Reverse Primer
*IL18RAP*	CCAGGGGTGAATAATTCTGGGT	CATTTGTCTGGGGCTTAACTTCT
*IL1B*	TTCGACACATGGGATAACGAGG	TTTTGCTGTGAGTCCCGGAG
*TNF*	CCTCTCTCTAATCAGCCCTCTG	GAGGACCTGGGAGTAGATGAG
*TREM1*	GAACTCCGAGCTGCAACTAAA	TCTAGCGTGTAGTCACATTTCAC
*EGR3*	CCAACGACATGGGCTCCATT	GGTCTCCAGAGGGGTAATAGG
*GAPDH*	GAGTCAACGGATTTGGTCGT	GACAAGCTTCCCGTTCTCAGCC

### GO enrichment and pathway analysis

DEGs were submitted to Visualisation and Integrated Discovery analysis (DAVID, version 6.8) (Huang da et al., 2009) for GO term enrichment and pathway analysis using default parameters. Kyoto Encyclopedia of Genes and Genomes and Reactome pathway analyses were used to determine the pathways of DEGs between two groups. Any GO terms and pathways with *P* < 0.05 were considered significantly enriched.

### Construction of PPI network and module analysis

Associations between DEGs were investigated using the Search Tool for the Retrieval of Interacting Genes/Proteins (STRING) (Szklarczyk et al., 2017) (STRING10.5, http://string-db.org/), and a confidence score >0.6 was considered to indicate significance. Cytoscape software (version 3.6.1) was then used to visualize the PPI network (Shannon et al., 2003). In the network, nodes represented proteins, and lines (edges) represented the interactions. In addition, the most significant modules were identified with the plug-in Molecular Complex Detection (version 1.5.1) with the following settings: degree cutoff of 2, node score cutoff of 0.2, k-core of 2, and a maximum depth of 100, and they were identified by the following criteria: Molecular Complex Detection score >5 and number of nodes >5. Finally, the hub genes in the PPI network were determined, defined as those with a degree of connectivity ≥8.

### Data availability statement

Datasets related to this article can be found at https://osf.io/hfwyt/?viewonly=ececc20afd5d42e2806b11483edb9d0d, hosted at The Open Science Framework.

## ORCIDs

Janna Nousbeck: http://orcid.org/0000-0001-6918-8855

Alan D. Irvine: http://orcid.org/0000-0002-9048-2044

Maeve A. McAleer: http://orcid.org/0000-0001-9958-6504

## Conflict of Interest

ADI is a consultant/speaker for Sanofi, Regeneron, Lilly, AbbVie, Pfizer, Benevolent AI, Almirall, LEO, and Arena. The remaining authors state no conflict of interest.

## References

[bib1] Bernard M., Carrasco C., Laoubi L., Guiraud B., Rozières A., Goujon C. (2017). IL-1β induces thymic stromal lymphopoietin and an atopic dermatitis-like phenotype in reconstructed healthy human epidermis. J Pathol.

[bib2] Bieber T. (2008). Atopic dermatitis. N Engl J Med.

[bib3] Brunner P.M., Israel A., Leonard A., Pavel A.B., Kim H.J., Zhang N. (2019). Distinct transcriptomic profiles of early-onset atopic dermatitis in blood and skin of pediatric patients. Ann Allergy Asthma Immunol.

[bib4] Brunner P.M., Israel A., Zhang N., Leonard A., Wen H.C., Huynh T. (2018). Early-onset pediatric atopic dermatitis is characterized by TH2/TH17/TH22-centered inflammation and lipid alterations. J Allergy Clin Immunol.

[bib5] Brunner P.M., Suárez-Fariñas M., He H., Malik K., Wen H.C., Gonzalez J. (2017). The atopic dermatitis blood signature is characterized by increases in inflammatory and cardiovascular risk proteins. Sci Rep.

[bib6] Cañete J.D., Pablos J.L., Sanmartí R., Mallofré C., Marsal S., Maymó J. (2004). Antiangiogenic effects of Anti-tumor necrosis factor alpha therapy with infliximab in psoriatic arthritis. Arthritis Rheum.

[bib7] Capone K.A., Dowd S.E., Stamatas G.N., Nikolovski J. (2011). Diversity of the human skin microbiome early in life. J Invest Dermatol.

[bib8] Cole C., Kroboth K., Schurch N.J., Sandilands A., Sherstnev A., O'Regan G.M. (2014). Filaggrin-stratified transcriptomic analysis of pediatric skin identifies mechanistic pathways in patients with atopic dermatitis. J Allergy Clin Immunol.

[bib9] Danso M.O., van Drongelen V., Mulder A., van Esch J., Scott H., van Smeden J. (2014). TNF-α and Th2 cytokines induce atopic dermatitis-like features on epidermal differentiation proteins and stratum corneum lipids in human skin equivalents. J Invest Dermatol.

[bib10] Dobin A., Davis C.A., Schlesinger F., Drenkow J., Zaleski C., Jha S. (2013). STAR: ultrafast universal RNA-seq aligner. Bioinformatics.

[bib11] Esaki H., Brunner P.M., Renert-Yuval Y., Czarnowicki T., Huynh T., Tran G. (2016). Early-onset pediatric atopic dermatitis is TH2 but also TH17 polarized in skin. J Allergy Clin Immunol.

[bib12] Evrard C., Faway E., De Vuyst E., Svensek O., De Glas V., Bergerat D. (2021). Deletion of TNFAIP6 gene in human keratinocytes demonstrates a role for TSG-6 to retain hyaluronan inside epidermis. JID Innov.

[bib13] Ewald D.A., Malajian D., Krueger J.G., Workman C.T., Wang T., Tian S. (2015). Meta-analysis derived atopic dermatitis (MADAD) transcriptome defines a robust AD signature highlighting the involvement of atherosclerosis and lipid metabolism pathways. BMC Med Genomics.

[bib14] Goedkoop A.Y., Kraan M.C., Picavet D.I., de Rie M.A., Teunissen M.B., Bos J.D. (2004). Deactivation of endothelium and reduction in angiogenesis in psoriatic skin and synovium by low dose infliximab therapy in combination with stable methotrexate therapy: a prospective single-centre study. Arthritis Res Ther.

[bib15] He H., Suryawanshi H., Morozov P., Gay-Mimbrera J., Del Duca E., Kim H.J. (2020). Single-cell transcriptome analysis of human skin identifies novel fibroblast subpopulation and enrichment of immune subsets in atopic dermatitis. J Allergy Clin Immunol.

[bib16] Higashi N., Gesser B., Kawana S., Thestrup-Pedersen K. (2001). Expression of IL-18 mRNA and secretion of IL-18 are reduced in monocytes from patients with atopic dermatitis. J Allergy Clin Immunol.

[bib17] Hillgruber C., Steingräber A.K., Pöppelmann B., Denis C.V., Ware J., Vestweber D. (2014). Blocking von Willebrand factor for treatment of cutaneous inflammation. J Invest Dermatol.

[bib18] Hiraga K., Kikuchi G. (1980). The mitochondrial glycine cleavage system. Functional association of glycine decarboxylase and aminomethyl carrier protein. J Biol Chem.

[bib19] Hirota T., Takahashi A., Kubo M., Tsunoda T., Tomita K., Sakashita M. (2012). Genome-wide association study identifies eight new susceptibility loci for atopic dermatitis in the Japanese population. Nat Genet.

[bib20] Huang da W., Sherman B.T., Lempicki R.A. (2009). Systematic and integrative analysis of large gene lists using DAVID bioinformatics resources. Nat Protoc.

[bib21] Hyder L.A., Gonzalez J., Harden J.L., Johnson-Huang L.M., Zaba L.C., Pierson K.C. (2013). TREM-1 as a potential therapeutic target in psoriasis. J Invest Dermatol.

[bib22] Jacobi A., Antoni C., Manger B., Schuler G., Hertl M. (2005). Infliximab in the treatment of moderate to severe atopic dermatitis. J Am Acad Dermatol.

[bib23] Jeong S.H., Kim H.J., Jang Y., Ryu W.I., Lee H., Kim J.H. (2014). Egr-1 is a key regulator of IL-17A-induced psoriasin upregulation in psoriasis. Exp Dermatol.

[bib24] Konishi H., Tsutsui H., Murakami T., Yumikura-Futatsugi S., Yamanaka K., Tanaka M. (2002). IL-18 contributes to the spontaneous development of atopic dermatitis-like inflammatory skin lesion independently of IgE/stat6 under specific pathogen-free conditions. Proc Natl Acad Sci USA.

[bib25] Lamkanfi M., Vande Walle L., Kanneganti T.D. (2011). Deregulated inflammasome signaling in disease. Immunol Rev.

[bib26] Langan S.M., Irvine A.D., Weidinger S. (2020). Atopic dermatitis [published correction appears in Lancet 2020;396:758. Lancet.

[bib27] Li S., Miao T., Sebastian M., Bhullar P., Ghaffari E., Liu M. (2012). The transcription factors Egr2 and Egr3 are essential for the control of inflammation and antigen-induced proliferation of B and T cells. Immunity.

[bib28] Liao Y., Smyth G.K., Shi W. (2014). featureCounts: an efficient general purpose program for assigning sequence reads to genomic features. Bioinformatics.

[bib29] Liu Y., Luo W., Chen S. (2011). Comparison of gene expression profiles reveals aberrant expression of FOXO1, Aurora A/B and EZH2 in lesional psoriatic skins. Mol Biol Rep.

[bib30] Livak K.J., Schmittgen T.D. (2001). Analysis of relative gene expression data using real-time quantitative PCR and the 2(-Delta Delta C(T)) Method. Methods.

[bib31] Martin M. (2011). CUTADAPT removes adapter sequences from high-throughput sequencing reads. EMBnet j.

[bib32] McAleer M.A., Jakasa I., Hurault G., Sarvari P., McLean W.H.I., Tanaka R.J. (2019). Systemic and stratum corneum biomarkers of severity in infant atopic dermatitis include markers of innate and T helper cell-related immunity and angiogenesis. Br J Dermatol.

[bib33] Morita K., Okamura T., Sumitomo S., Iwasaki Y., Fujio K., Yamamoto K. (2016). Emerging roles of Egr2 and Egr3 in the control of systemic autoimmunity. Rheumatology (Oxford).

[bib34] Nakamura Y., Takahashi H., Takaya A., Inoue Y., Katayama Y., Kusuya Y. (2020). Staphylococcus Agr virulence is critical for epidermal colonization and associates with atopic dermatitis development. Sci Transl Med.

[bib35] Niebuhr M., Baumert K., Heratizadeh A., Satzger I., Werfel T. (2014). Impaired NLRP3 inflammasome expression and function in atopic dermatitis due to Th2 milieu. Allergy.

[bib36] Nousbeck J., McAleer M.A., Hurault G., Kenny E., Harte K., Kezic S. (2021). MicroRNA analysis of childhood atopic dermatitis reveals a role for miR-451a. Br J Dermatol.

[bib37] Oka T., Sugaya M., Takahashi N., Takahashi T., Shibata S., Miyagaki T. (2017). CXCL17 attenuates imiquimod-induced psoriasis-like skin inflammation by recruiting myeloid-derived suppressor cells and regulatory T cells. J Immunol.

[bib38] Rittié L., Tejasvi T., Harms P.W., Xing X., Nair R.P., Gudjonsson J.E. (2016). Sebaceous gland atrophy in psoriasis: an explanation for psoriatic alopecia?. J Invest Dermatol.

[bib39] Roe K., Gibot S., Verma S. (2014). Triggering receptor expressed on myeloid cells-1 (TREM-1): a new player in antiviral immunity?. Front Microbiol.

[bib40] Schroder K., Tschopp J. (2010). The inflammasomes. Cell.

[bib41] Shannon P., Markiel A., Ozier O., Baliga N.S., Wang J.T., Ramage D. (2003). Cytoscape: a software environment for integrated models of biomolecular interaction networks. Genome Res.

[bib42] Suárez-Fariñas M., Tintle S.J., Shemer A., Chiricozzi A., Nograles K., Cardinale I. (2011). Nonlesional atopic dermatitis skin is characterized by broad terminal differentiation defects and variable immune abnormalities. J Allergy Clin Immunol.

[bib43] Suárez-Fariñas M., Ungar B., Correa da Rosa J., Ewald D.A., Rozenblit M., Gonzalez J. (2015). RNA sequencing atopic dermatitis transcriptome profiling provides insights into novel disease mechanisms with potential therapeutic implications. J Allergy Clin Immunol.

[bib44] Sumimoto S., Kawai M., Kasajima Y., Hamamoto T. (1992). Increased plasma tumour necrosis factor-alpha concentration in atopic dermatitis. Arch Dis Child.

[bib45] Szklarczyk D., Morris J.H., Cook H., Kuhn M., Wyder S., Simonovic M. (2017). The STRING database in 2017: quality-controlled protein-protein association networks, made broadly accessible. Nucleic Acids Res.

[bib46] Tamagawa-Mineoka R., Katoh N., Ueda E., Takenaka H., Kita M., Kishimoto S. (2007). The role of platelets in leukocyte recruitment in chronic contact hypersensitivity induced by repeated elicitation. Am J Pathol.

[bib47] Thijs J.L., Strickland I., Bruijnzeel-Koomen C.A.F.M., Nierkens S., Giovannone B., Knol E.F. (2018). Serum biomarker profiles suggest that atopic dermatitis is a systemic disease. J Allergy Clin Immunol.

[bib48] Watanabe O., Natori K., Tamari M., Shiomoto Y., Kubo S., Nakamura Y. (1999). Significantly elevated expression of PF4 (platelet factor 4) and eotaxin in the NOA mouse, a model for atopic dermatitis. J Hum Genet.

[bib49] Weidinger S., Beck L.A., Bieber T., Kabashima K., Irvine A.D. (2018). Atopic dermatitis. Nat Rev Dis Primers.

[bib50] Zhang W.C., Shyh-Chang N., Yang H., Rai A., Umashankar S., Ma S. (2012). Glycine decarboxylase activity drives non-small cell lung cancer tumor-initiating cells and tumorigenesis. Cell.

[bib51] Zimmermann M., Koreck A., Meyer N., Basinski T., Meiler F., Simone B. (2011). TNF-like weak inducer of apoptosis (TWEAK) and TNF-α cooperate in the induction of keratinocyte apoptosis. J Allergy Clin Immunol.

